# Planar Chiral Carbazole‐Naphthalene Bisimide Hetero‐Cyclophane for Circularly Polarized Delayed Fluorescence

**DOI:** 10.1002/anie.2300390

**Published:** 2026-07-06

**Authors:** Sanchari Debnath, Swadhin Garain, Yvonne Wagenhäuser, Frank Würthner

**Affiliations:** ^1^ Center For Nanosystems Chemistry (CNC) Universität Würzburg Würzburg Germany; ^2^ Institut Für Organische Chemie Universität Würzburg Würzburg Germany

**Keywords:** delayed fluorescence, hetero‐cyclophane, photochemistry, planar chirality, supramolecular chemistry

## Abstract

Chiral organic chromophores with circularly polarized thermally activated delayed fluorescence (CP–TADF) provide great prospects as circularly polarized luminescence (CPL) emitters owing to the involvement of long‐lived triplet excitons. Here, we construct a tailored planar chiral hetero‐cyclophane (**NBICz–Cy**) composed of 1,2:5,6–naphthalene bisimide (1,2:5,6–NBI) and carbazole (Cz) units, which functions as an efficient solution‐state CP–TADF emitter. Comprehensive photophysical studies reveal pronounced delayed fluorescence in the orange‐red region, with a quantum yield (QY) of 19% in deaerated solution and 12% in an aerated solution‐processable matrix. Single‐crystal XRD analysis unravels a through‐space charge‐transfer (CT) interaction between the NBI and Cz moieties, being responsible for delayed fluorescence. The inherent prochirality of the 1,2:5,6–NBI moiety imparts planar chirality in the cyclophane framework, resulting in CP–TADF in both solution and solid state with |g_lum_| values of ∼ 2.5 – 3.3 × 10^−3^.

## Graphical abstract

Tightly stacked carbazole and 1,2:5,6–naphthalene bisimide units afforded a planar chiral hetero‐cyclophane. The architecturally refined system, with strong charge‐transfer interaction and enhanced structural rigidity, exhibits orange‐red delayed CPL emission in both solution and solid states.

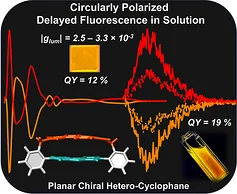

## 1 Introduction

Purely organic circularly polarized luminescence (CPL) emitters have attracted significant attention for next‐generation display technologies, enabling circularly polarized emission without external polarizers [1, 2, 3]. Beyond displays, chiral organic luminogens are being explored for applications in chiral sensing, optical data storage, spintronics, and bioimaging [4, 5, 6]. Traditionally, CPL has been achieved from the singlet excited state using helical or axially chiral organic chromophores [7, 8, 9, 10]. However, purely organic systems often suffer from low luminescence dissymmetry factors (|g_lum_|) due to the inherently weaker magnetic (|*m*|) and stronger electric transition dipole moment (|*μ*|) [1, 4]. To overcome this limitation and enhance |g_lum_| values, researchers have explored supramolecular strategies [11] such as charge‐transfer (CT) interactions [12, 13, 14, 15], and excimer [16, 17, 18] formation. At the same time, the field began to blossom upon introducing thermally activated delayed fluorescence (TADF) [19] emission in CPL materials due to their promising ability to involve the triplet state in CPL luminogens [20, 21, 22]. In this context, incorporation of the [2.2]paracyclophane moiety has been demonstrated as an effective strategy toward circularly polarized thermally activated delayed fluorescence (CP–TADF) materials [23, 24, 25, 26] by merging the planar chiral paracyclophane units with *N*‐heterocyclic donors of established triazine acceptor‐based TADF [27, 28, 29, 30, 31] or multi‐resonance (MR) TADF [32, 33, 34] emitter cores. However, such TADF active CPL emitters typically show poor CPL efficiency in solutions under aerated conditions due to a limited triplet exciton contribution. As a result, delayed CPL emission in the solution state remains unexplored. To harvest the triplet exciton in the solution state, we and others recently demonstrated the concept of a supramolecular nano‐environment where the triplet exciton stabilization in the solution state is possible by minimizing vibrational dissipation and oxygen penetration, which leads to efficient color‐tunable TADF emission from the CT state upon modulation of the guest [35, 36, 37, 38]. Despite the advantages of such non‐covalent host–guest systems, their dynamic nature and relatively low binding affinities limit the development of advanced photofunctional materials. To address this issue, we hypothesize that using preorganized planar chiral supramolecular architectures [39, 40] may allow for the simultaneous realization of delayed fluorescence and chiroptical activity, with improved |g_lum_| values. Our objective is to take advantage of the orchestrating interaction between the two chromophores to form an emissive CT state for delayed fluorescence to happen.

Herein, we propose a new 1,2:5,6–naphthalene bisimide (1,2:5,6–NBI) derived planar chiral hetero‐cyclophane (**NBICz–Cy**) which can combine the benefits of supramolecular engineering with enhanced structural rigidity, paving the way for more robust and efficient CP–TADF materials. As a result, **NBICz–Cy** shows orange‐red delayed fluorescence originating from the emissive CT state with a quantum yield (QY) of ∼19% in carbon tetrachloride (CCl_4_) under deaerated conditions and ∼ 12% in polymethylmethacrylate (PMMA) matrix under air. The chiral nature of the molecule led to mirror‐image circular dichroism (CD) and orange‐red CPL from the enantiomers with |g_lum_| values of ∼2.5 × 10^−3^ in CCl_4_ under deaerated conditions and ∼ 3.3 × 10^−3^ in the solution processable polymer matrix PMMA under ambient conditions.

## 2 Results and Discussion

**SCHEME 1:**
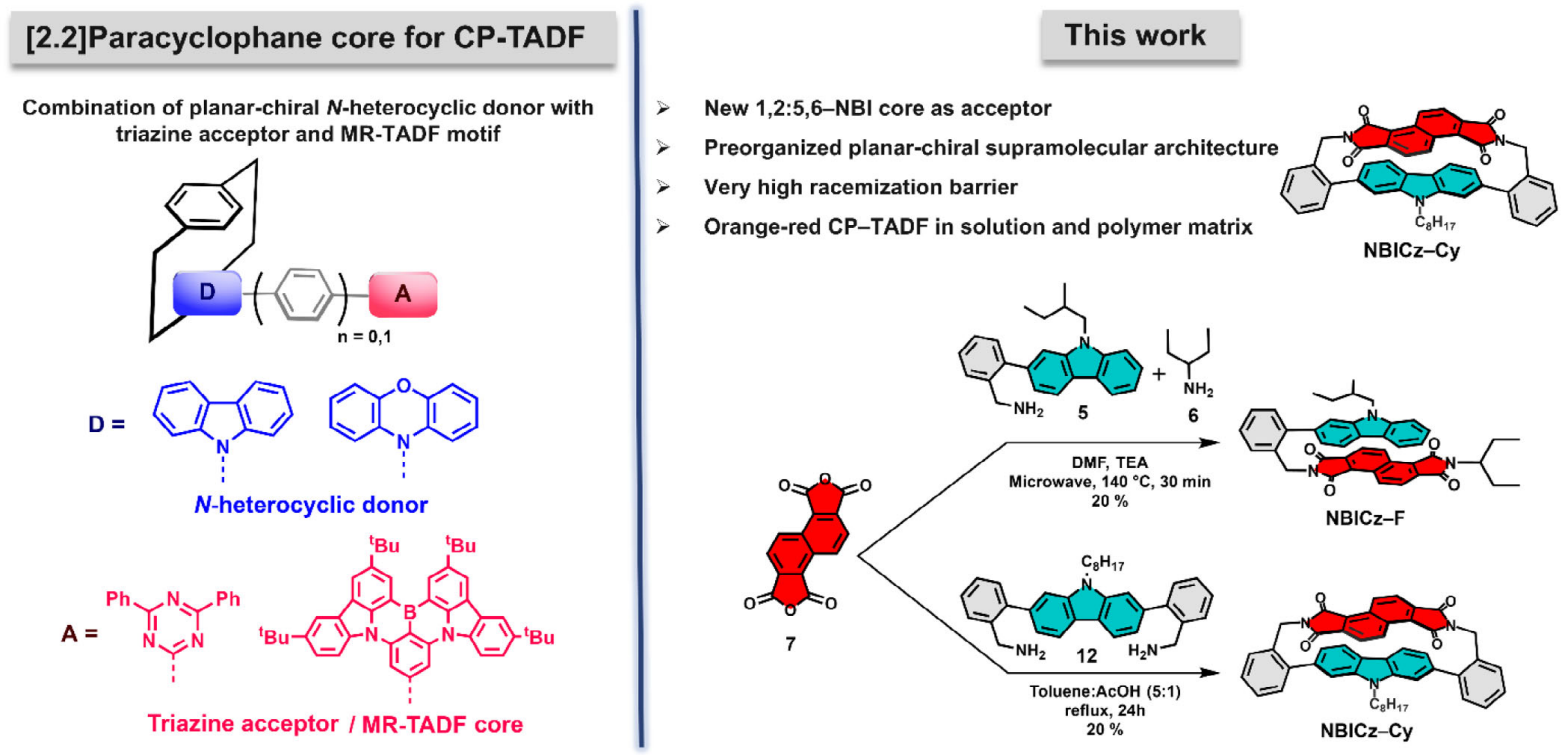
Molecular design principles of [2.2]paracyclophane‐based CP–TADF molecules and our preorganized planar chiral hetero‐cyclophane design and synthesis of flexible **NBICz–F** foldamer and rigid **NBICz–Cy** hetero‐cyclophane.

**FIGURE 1:**
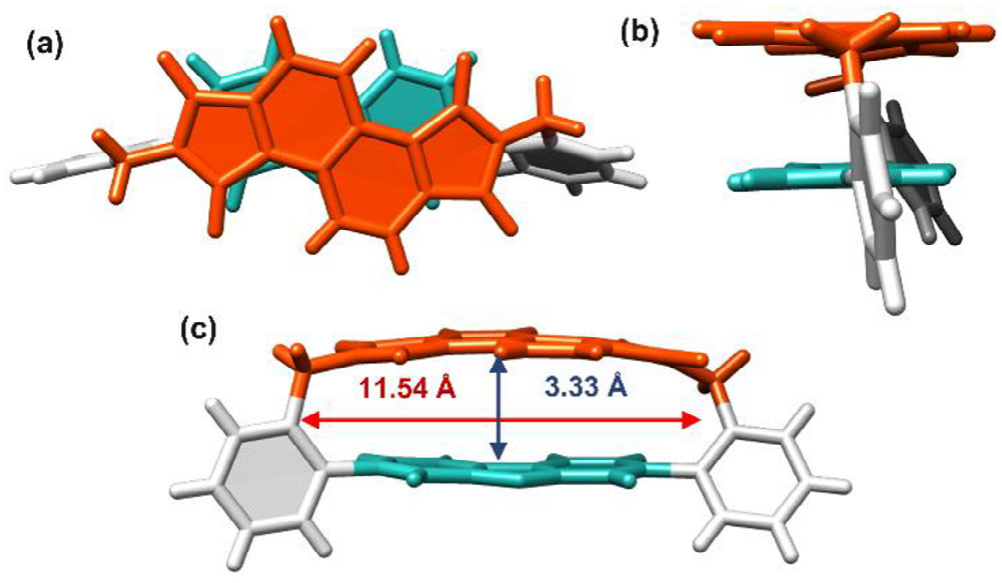
(a) Top, (b) side, and (c) front view of the **NBICz–Cy** in single‒crystals (alkyl chain and solvent molecules are removed for clarity).

We have synthesized 1,2:5,6–NBI acceptor and carbazole donor‐derived hetero‐cyclophane (**NBICz–Cy**) and the less rigidified foldamer (**NBICz–F**) via a multistep synthetic route (Schemes 1, S1, and S2), where for **NBICz–Cy**, the final cyclization reaction was performed by one‐pot imidization of naphtho[1,2‐*c*:5,6‐*c'*]difuran‐1,3,6,8‐tetraone and ((9‐octyl‐9*H*‐carbazole‐2,7‐diyl)bis(2,1 phenylene))dimethanamine in an acetic acid:toluene (1:5) mixture. We have fully characterized **NBICz–F** and **NBICz–Cy** by ^1^H, ^13^C nuclear magnetic resonance (NMR) spectroscopy and mass spectrometry (see Supporting Information). To obtain detailed structural insights of **NBICz–Cy**, we have grown single crystals upon slow vapour diffusion of methanol into a saturated chloroform solution (Figures 1, S1, and S2; Table S1). Single‐crystal X‐ray analysis revealed that **NBICz–Cy**, as a racemic mixture, formed a co‐facially stacked architecture in space group P‐1, with average distances between NBI and Cz planes of 3.33 Å and a longitudinal extension of 11.54 Å, respectively (Figure 1c). The *ortho*–benzyl amine linker helped to achieve a strong π–π interaction between electron‐deficient NBI and electron‐rich Cz units at distances of 2.92–3.38 Å to facilitate through‐space CT interaction, which is also confirmed by theoretical calculations according to Figure S3.

**FIGURE 2:**
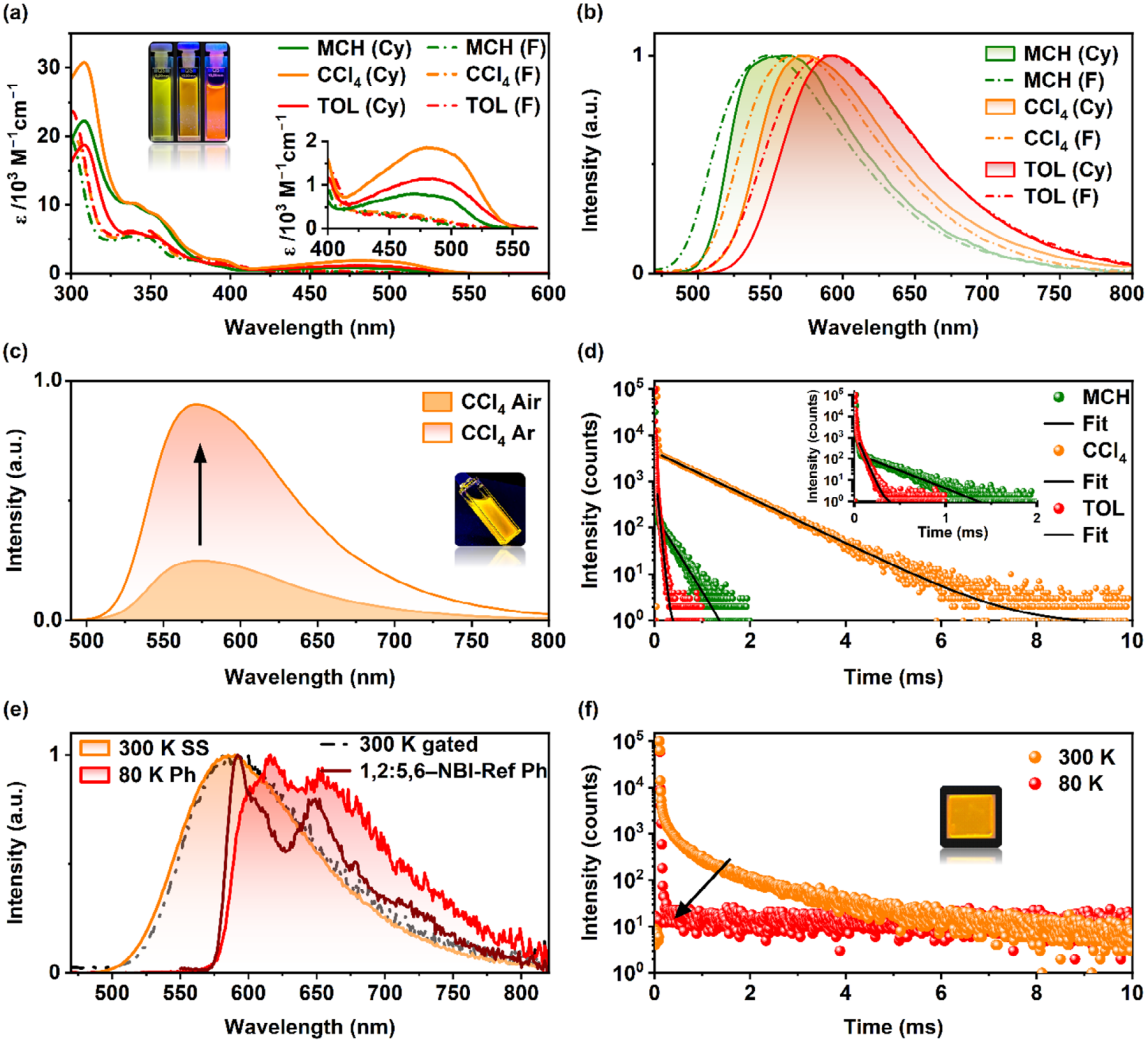
(a) UV–Visible absorption spectra and (b) normalized steady state emission spectra of **NBICz–F** and **NBICz–Cy** in methylcyclohexane (MCH), carbon tetrachloride (CCl_4_), and toluene (TOL) solvents, respectively (the dash‐dot and the solid lines correspond to **NBICz–F** and **NBICz–Cy**, respectively; Inset of (a): cuvettes showing emission of **NBICz–Cy** in MCH, CCl_4_, and TOL solutions from left to right, respectively, under 365 nm UV light excitation). (c) Changes in the emission spectra of **NBICz–Cy** in CCl_4_ solution under air and Ar atmosphere (inset: cuvette showing emission of **NBICz–Cy** in Ar purged CCl_4_ solution with enhancement in intensity). (d) Time‐resolved emission decay of **NBICz–Cy** in different solvents (c ∼ 1 × 10^−5^ M in all the cases, λ_exc._ = 450 nm in all the cases; λ_collected_ = 560, 575, and 595 nm for MCH, CCl_4_ and TOL, respectively. (e) Comparison of steady state (SS), delayed fluorescence (delay time = 50 µs) and phosphorescence (Ph) spectra (delay time = 2 ms) of **NBICz–Cy** thin film (1 wt.% doped in PMMA matrix) with the phosphorescence spectra of the reference compound (1,2:5,6–NBI‐Ref). (f) Temperature‐dependent delayed fluorescence lifetimes of **NBICz–Cy** in PMMA doped film (λ_exc__._ = 450 nm, λ_collected_ = 585 nm, inset: photograph of PMMA‐doped **NBICz–Cy** film under 365 nm UV light excitation).

In order to gain photophysical insights into the **NBICz–F** and **NBICz–Cy**, we first studied the optical properties in dilute solutions of different solvents, including methylcyclohexane (MCH), CCl_4_, and toluene (TOL) (Figures 2 and S4–S11; Tables 1, S2, and S3). We observed an intense π–π^*^ absorption band spanning ∼300 to 350 nm, accompanied by a weak CT band in the ∼ 400–450 nm range for both **NBICz–F** and **NBICz–Cy** chromophores (Figures 2a, S4, and S5). However, the CT band is much weaker in the case of **NBICz–F**, compared to **NBICz–Cy**, which could be attributed to enhanced rigidification in the cyclophane architecture, compared to the flexible foldamer design. The corresponding emission spectra in MCH, CCl_4_, and TOL are centred around ∼550, 570, and 590 nm for **NBICz–F**, and ∼560, 575, and 595 nm for **NBICz–Cy**, respectively (Figures 2b and S7‒S9). The broad emission profiles and solvatochromic shift with increasing solvent polarity further support the CT nature of the emission. From theoretical calculations, it is evident that the highest occupied natural transition orbital (HONTO) is located on the electron‐rich Cz core and the lowest unoccupied natural transition orbital (LUNTO) is located on the electron‐deficient NBI moiety, clearly indicating the CT nature of the transition (Figure S3). In an ambient solution, **NBICz–F** and **NBICz–Cy** display relatively low QYs of 2%–3% and 4%–8%, respectively, which could be attributed to intersystem crossing (ISC) promoted by the CT state (Table 1). To gain deeper insights, we have performed a detailed photophysical investigation under deaerated conditions (Figures 2c, and S7‒S9; Table 1). The QY increased from 3.5% to 4.7% (in CCl_4_), 2.6% to 3.8% (in TOL) for **NBICz–F** and 4.1% to 7.6% (in MCH), 8% to 19% (in CCl_4_), 4.5% to 7.8% (in TOL) for **NBICz–Cy** from aerated to deaerated conditions, respectively (Table 1). The enhancement in emission intensity upon Ar purging hints towards involvement of triplet excitons in the emission process. Further, the presence of delayed emission spectra and high lifetimes (several µs) confirmed the delayed nature of the emission (Figures 2d, S5, S7, and S8; Table 1). It is important to mention here that we did not observe any delayed fluorescence lifetime in MCH for **NBICz–F**. Whilst upon Ar purging, there is a significant increase in prompt fluorescence lifetime, which could be due to a decrease in diffusion‐controlled encounters with molecular oxygen in argon atmosphere (Figures S10 and S11; Tables S2 and S3) [41]. The calculated ISC and reverse intersystem crossing (rISC) rates are ∼10^7^ and 10^3–4^ s^−1^, respectively, which are responsible for delayed fluorescence in the solution state (Table 1). Interestingly, **NBICz–F** and **NBICz–Cy**, upon dispersing in PMMA matrix (1 wt.%), displayed orange‐red delayed fluorescence with QYs of 4% and 12%, respectively, under ambient conditions (Figures 2e and S12; Tables 1 and S4). Moreover, **NBICz–F** and **NBICz–Cy** in amorphous neat film also displayed orange‐red delayed fluorescence under ambient conditions with QYs of 5% and 10%, respectively, beneficial for circularly polarized organic light‐emitting diode (CP‐OLEDs) technology (Figures S13 and S14; Tables 1 and S5). Despite their similar photophysical properties in different solvents and in the solid state, **NBICz–Cy** displays consistently better photophysical performance compared to **NBICz–F** due to enhanced structural rigidity in the cyclophane structure.

**TABLE 1:** Summary of photophysical properties of **NBICz–F** and **NBICz–Cy** at room temperature (293 K).

System	Solvent/ Matrix	λ_emission_ (nm)	τ_PF_ (ns)	τ_DF_ (µs)	Φ_PF_ (%)	Φ_DF_ (%)	Φ_Total_ (%)	Φ_ISC_ (%)	k_F_ (s^−1^)	k_ISC_ (s^−1^)	k_rISC_ (s^−1^)
**NBICz–F**	CCl_4_	570	22.3	810	3.5	1.2	4.7	25	1.5 × 10^6^	1.1 × 10^7^	1.7 × 10^3^
	TOL	590	22.8	1005	2.6	1.2	3.8	32	1.1 × 10^6^	1.4 × 10^7^	1.4 × 10^3^
	PMMA	565	23.0	808	—	—	4.0	—	—	—	—
**NBICz–Cy**	MCH	560	23.0	264	4.1	3.5	7.6	46	1.8 × 10^6^	2.0 × 10^7^	7.0 × 10^3^
	CCl_4_	575	22.5	881	8.0	11.0	19.0	42	3.5 × 10^6^	1.8 × 10^7^	3.7 × 10^3^
	TOL	595	20.2	44	4.5	3.3	7.8	42	2.2 × 10^6^	2.1 × 10^7^	3.9 × 10^4^
	PMMA	585	21.0	962	—	—	12.0	—	—	—	—

τ_PF_ = prompt fluorescence lifetime, τ_DF_ = delayed fluorescence lifetime, Φ_PF_ = prompt fluorescence quantum yield, Φ_DF_ = delayed fluorescence quantum yield, Φ_ISC_ = ISC quantum yield, k_F_ = fluorescence rate, k_ISC_ = ISC rate, k_rISC_ = rISC rate. Φ_ISC,_ k_ISC and_ k_rISC_ were calculated from prompt and delayed fluorescence quantum yield and lifetime according to the following equations: Φ_ISC_ = Φ_DF_/(Φ_DF_ + Φ_PF_), k_ISC_ (s^−1^) = Φ_ISC_/τ_PF_, and k_rISC_ (s^−1^) = (Φ_DF_/Φ_PF_)/(Φ_ISC_×τ_DF_).

To elucidate the mechanism of delayed fluorescence, we performed temperature‐dependent measurements of **NBICz–Cy** in a dispersed PMMA matrix at a very low molecular concentration (1 wt.%), which revealed a significant decrease in the delayed fluorescence lifetime with decreasing temperature from 300 to 80 K, indicating its thermally activated origin. (Figure 2f and S15–S17; Table S6). The phosphorescence spectrum of **NBICz–Cy** exhibits a vibronic feature and displays a prominent resemblance to the phosphorescence spectra of the acceptor core (^3^LE of 1,2:5,6–NBI‐Ref), indicating its locally excited (LE) nature (Figure 2e). Furthermore, theoretical calculations demonstrate the LE nature of the lowest energy triplet excited state (T_1_) for the **NBICz–Cy**, which corroborates our experimental observation (Figure S3). The experimentally calculated singlet and triplet energies of **NBICz–Cy** are ∼2.37 and ∼2.14 eV, respectively, thereby having a ΔE_ST_ of ∼0.23 eV, which is sufficient for efficient rISC process (Figure S18).

**FIGURE 3:**
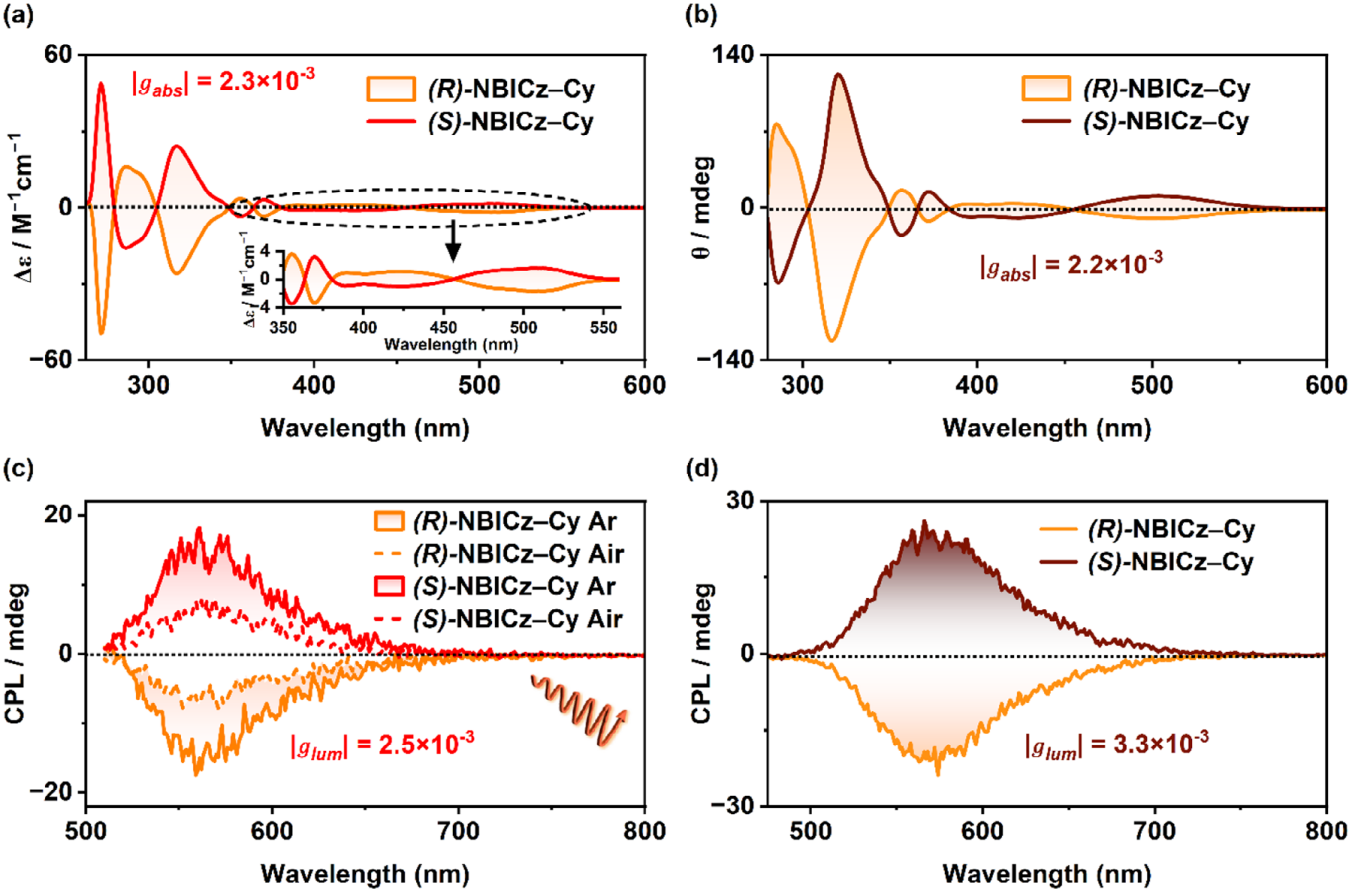
(a) Mirror‐image electronic circular dichroism (ECD) and (c) circularly polarized luminescence (CPL) spectra of **NBICz–Cy** enantiomers in CCl_4_ solution at 293 K under aerated and deaerated conditions (Ar was bubbled into the solution before the measurement, c ∼ 1 × 10^−4^ M for both cases, λ_exc._ = 370 nm). (b) Mirror‐image CD and (d) CPL spectra of 1 wt. % **NBICz–Cy** enantiomers doped in PMMA matrix at 293 K, λ_exc__._ = 370 nm).

In order to elucidate the inherent chirality of the cyclophane framework and gain deeper insight into its distinctive chiroptical behavior, we successfully resolved the two enantiomers of **NBICz–Cy** using recycling semipreparative chiral HPLC (Figure S19). Subsequently, we explored the chiroptical properties in both the ground and excited states in solution as well as in the solid state (Figures 3 and S20–S36). The inherent asymmetry of the 1,2:5,6–NBI moiety renders the resulting hetero‐cyclophane framework devoid of any symmetry elements, lacking both mirror planes and an inversion center. In addition, a short intramolecular π–π distance in combination with the covalent linkage in the cyclophane architecture restricts the rotation of both the 1,2:5,6–NBI and Cz moiety, resulting in a very high racemization barrier (Figures S28–S31). The enantiomers of the **NBICz–Cy** exhibited mirror‐image CD signal with prominent Cotton effects (with maxima at 500, 420, 370, 355, and 315 nm), demonstrating pronounced chiroptical activity in the ground state (Figures 3a and S23–S26). Notably, the first Cotton effect appeared in the red‐shifted absorption band, arising from through‐space CT interaction, with an absorption dissymmetry factor (|g_abs_|) of 0.5 × 10^−3^ in CCl_4_. The maximum |g_abs_| values are 2.3 × 10^−3^ and 2.5 × 10^−3^, corresponding to the π–π^*^ absorption maxima in dilute CCl_4_ and toluene solutions, respectively (Figure S26). The experimentally observed CD spectra were in excellent agreement with simulated spectra, which helped us to assign the absolute configurations of the enantiomers (Figure S22). The excited state chiroptical properties showed mirror‐image CPL spectra originating from the emissive CT state, with maxima at 560 and 583 nm in CCl_4_ and TOL solutions, respectively, and a |g_lum_| of 2.5 × 10^−^^3^ under ambient conditions (Figures 3c, S24, S25, and S27). Remarkably, the CPL emission intensity increased significantly under an inert atmosphere, attributed to the contribution of triplet excitons to the overall CPL emission. Furthermore, we have observed similar chiroptical properties upon dispersion of the **NBICz–Cy** enantiomers into a PMMA matrix, both in ground and excited states (Figures 3b,d, and S32–S36). The PMMA‐doped films showed mirror‐image CD signal, with |g_abs_| values of –2.6 × 10^−^^3^ and +2.2 × 10^−^^3^ for the (R)‐ and (S)‐isomers, respectively (Figure 3b). Corresponding films emit orange‐red CPL with a maximum at ∼570 nm and a |g_lum_| value of 3.3 × 10^−3^ (Figures 3d and S33–S35). This observation represents the visualization of CPL enhancement mediated by triplet excitons from solution to the solid state. The notable CPL activity of **NBICz–Cy**, both in solution and in the PMMA matrix, can be rationalized by the involvement of a CT state characterized by a relatively large |*m*| arising from unpaired electron density, together with a small |*μ*|, consistent with the weak CT absorption band observed experimentally (Figure S3 and Table S7).

## 3 Conclusion

In conclusion, we introduced a supramolecularly engineered planar chiral **NBICz–Cy** hetero‐cyclophane as a dual solution and solid‐state CP–TADF emitter. Single‐crystal XRD analysis unveiled a tightly π‐stacked arrangement between the 1,2:5,6–NBI and Cz units, forming efficient through‐space CT interactions that establish an emissive CT state, resulting in delayed fluorescence in solution, as confirmed by comprehensive photophysical investigations in both solution and solid states. Moreover, the overall rigid cyclophane architecture, lacking any internal symmetry elements, imparts planar chirality and allows for the isolation of enantiopure **NBICz–Cy** through chiral resolution. As a result, we realized ground‐ and excited‐state chiroptical properties evident from the mirror‐image CD and CPL spectra in solution and solid state with a |g_lum_| of ∼ 2.5 – 3.3 × 10^−^^3^. Notably, argon purging led to a significant enhancement in CPL intensity, suggesting a contribution from triplet excitons to the excited state chiroptical process. We envision that this planar chiral hetero‐cyclophane framework offers a versatile blueprint for developing a wide range of chiral cyclophane architectures, paving the way for advanced CP–OLED applications in the near future.

## Conflicts of Interest

The authors declare no conflicts of interest.

## Supporting information




**Supporting File 1**: anie73509‐sup‐0001‐SuppMat.pdf.

## Data Availability

Original data supporting this work is available free of charge at the Zenodo data repository under https://doi.org/10.5281/zenodo.18662208. Accession Codes: CCDC 2531458 contains the supplementary crystallographic data for this paper. These data can be obtained free of charge via www.ccdc.cam.ac.uk/data_request/cif, or by emailing data_request@ccdc.cam.ac.uk, or by contacting The Cambridge Crystallographic Data Centre, 12 Union Road, Cambridge CB2 1EZ, UK; fax: +44 1223 336033.
