# Supplementary material for: Planar Chiral Carbazole‐Naphthalene Bisimide Hetero‐Cyclophane for Circularly Polarized Delayed Fluorescence
Source: Angew Chem Int Ed Engl. 2026 Jul 6;65(35):e2300390. doi: 10.1002/anie.2300390 (PMC13502543; doi:10.1002/anie.2300390)
Supplement: Supplementary file 1 [file ANIE-65-e2300390-s001.pdf]

Supporting Information

Planar Chiral Carbazole-Naphthalene Bisimide Hetero-cyclophane for Circularly Polarized Delayed Fluorescence

Sanchari Debnath,[a]+ Swadhin Garain,[b]+ Yvonne Wagenhäuser,[b] and Frank Würthner*[a] [b]

[a] Dr. Sanchari Debnath and Prof. Dr. Frank Würthner
Center for Nanosystems Chemistry (CNC)
Universität Würzburg
97074 Würzburg, Germany
Email: frank.wuerthner@uni-wuerzburg.de

[b] Dr. Swadhin Garain, Dr. Yvonne Wagenhäuser and Prof. Dr. Frank Würthner
Institut für Organische Chemie
Universität Würzburg
97074 Würzburg, Germany

+ Dr. Sanchari Debnath and Dr. Swadhin Garain contributed equally to this work.

Table of contents

1. General Methods and Materials....................3
2. Synthetic Schemes and Procedures....................5
3. Crystallographic Analysis....................11
4. Theoretical Calculations....................13
5. Optical Properties....................14
6. Chiroptical Properties....................21
7. NMR Spectroscopy....................29
8. Mass Spectrometry....................39
9. References....................42

1. General Methods and Materials:

Chemicals: All chemicals and solvents were purchased from commercial suppliers and were used without any further purification.

Purification: Column chromatography was performed with commercial glass columns using silica gel (particle size 0.040–0.063 mm) with distilled solvents as eluents. The resolution of enantiomers for chiral **NBICz–Cy** was carried out with recycling semi-preparative HPLC on JAI LC-9105 using a Trentec Reprosil-100 Chiral-NR 8μm-column (using a solvent mixture of DCM: Hex = 75:25 as eluent). Gel permeation chromatography (GPC) was performed on a Shimadzu Recycling GPC-System (LC-20AD Prominence Pump; SPDMA20A Prominence Diode Array Detector) equipped with three preparative columns in a series (Japan Analytical Industries Co., Ltd.; JAIGEL-1 H, JAIGEL-2H and JAIGEL-2.5 H) using chloroform (HPLC grade, stabilized with 0.1 % EtOH) as eluent and 6 mL/min flow rate.

Microwave reactions: Microwave reactions were carried out in a Discover 2.0 microwave from *CEM* GmbH. The starting materials were placed in a special 10 mL pressurised flask using a stirring bar.

NMR spectroscopy: NMR spectra were recorded on a Bruker Avance III HD 400 MHz spectrometer. All spectra are referenced according to the residual peaks of deuterated solvent used for measuring the spectra, and chemical shifts are recorded in parts per million downfield from tetramethylsilane. The coupling constants (*J*) are listed in Hertz (Hz). Short notations used are s for singlet, d for doublet, t for triplet, q for quintet, and m for multiplet.

Mass spectrometry: MALDI-TOF mass spectra were measured on a Bruker Daltonics ultrafleXtreme mass spectrometer using trans-2-[3-(4-*tert*-butylphenyl)-2-methyl-2-propenylidene]malononitrile (DCTB) as matrix. ESI-TOF mass spectra were recorded on a Bruker Daltonics microTOF focus spectrometer.

Melting point: Melting points were determined using an Olympus BX41 polarization microscope connected to a TP84 Linkam scientific temperature regulator.

UV-vis absorption spectroscopy: UV-Visible absorption spectra were recorded on JASCO V-670 and V770 spectrometers with a PAC-743R Peltier for temperature control.

Fluorescence spectroscopy: Fluorescence spectra were recorded on an Edinburgh Instruments FLS980 fluorescence spectrometer and corrected against the photomultiplier sensitivity and the lamp intensity. Solution state emission spectra were recorded in a 10 mm path length cuvette. Fluorescence spectra of the films were recorded in front-face geometry to avoid self-absorption. All the spectroscopic studies were performed using spectroscopic-grade solvents. Prompt fluorescence lifetimes were measured on FLS980 using a 405 nm EPL picosecond pulsed laser diode. The instrument response function (IRF) was collected by using a scatterer (Ludox AS40 colloidal silica, Sigma-Aldrich). Delayed fluorescence and phosphorescence lifetime, gated emission, and time-resolved excitation and emission were measured on a FLS980 spectrometer, Edinburgh Instruments, equipped with a micro flash-lamp (μF_2) set-up. Quantum yields were determined relative to common fluorescence standards for optically dilute solutions (A < 0.05).[S1]

Circular dichroism (CD) spectroscopy and circularly polarized luminescence (CPL) spectroscopy: CD and CPL spectra were recorded with a customized JASCO CPL-300/J-1500 hybrid spectrometer. CD spectra included in the supporting information were measured with a Jasco J-810 spectropolarimeter equipped with a JASCO CDF-426S Peltier temperature controller.

Single crystal X-ray analysis: The single crystals of the **NBICz–Cy** hetero-cyclophane were grown by slow vapour diffusion of methanol into the chloroform solution. Single-crystal X-ray diffraction was performed at the P11 beamline at DESY synchrotron in Hamburg. The diffraction data were collected by a single 360° ϕ scan at 100 K. The diffraction data were indexed, integrated, and scaled by the XDS program package.[S2] The structures were solved using SHELXT,[S3] expanded with Fourier techniques, and refined using the SHELXL software package.[S4] Hydrogen atoms were assigned at idealized positions and included in the structure factor calculation. All non-hydrogen atoms were refined anisotropically. Due to the single-axis rotation at the crystallographic end station only

a low completeness is obtained for triclinic structures. Therefore, the datasets of the two crystals were merged with XPREP.[S5] The geometry of the three-fold disordered octyl substituent was constraint with DFIX and DANG. Additionally, SADI, CHIV, SAME, SIMU and RIGU commands were applied. The structure contains disordered solvent molecules (chloroform) around an inversion center. The geometry of the solvent molecules was constraint to the idealized one, and the ADPs were restraint by SIMU and RIGU.

Theoretical calculations: S_0 geometries were optimized using CAM-B3LYP-D3/def2-SVP and CD spectra from TD (S_1) calculations, including 15 singlet excited states. CD spectrum was simulated with a HWHM peak of 0.1 eV using GaussView. CAM-B3LYP-D3/def2-SVP optimized natural transition orbitals (NTOs) of vertical excitations from TD (S_1) and TDA (T_1 and T_2) calculations using Gaussian16.

2. Synthetic Schemes and Procedures:

Compound **1** (2-bromo-9*H*-carbazole), compound **7** (naphtho[1,2-*c*:5,6-*c*′]difuran-1,3,6,8-tetraone), compound **8** (2,7-dibromo-9*H*-carbazole), and *N*-Boc-2-Brombenzylamine were purchased from commercial suppliers and used without any further purification.

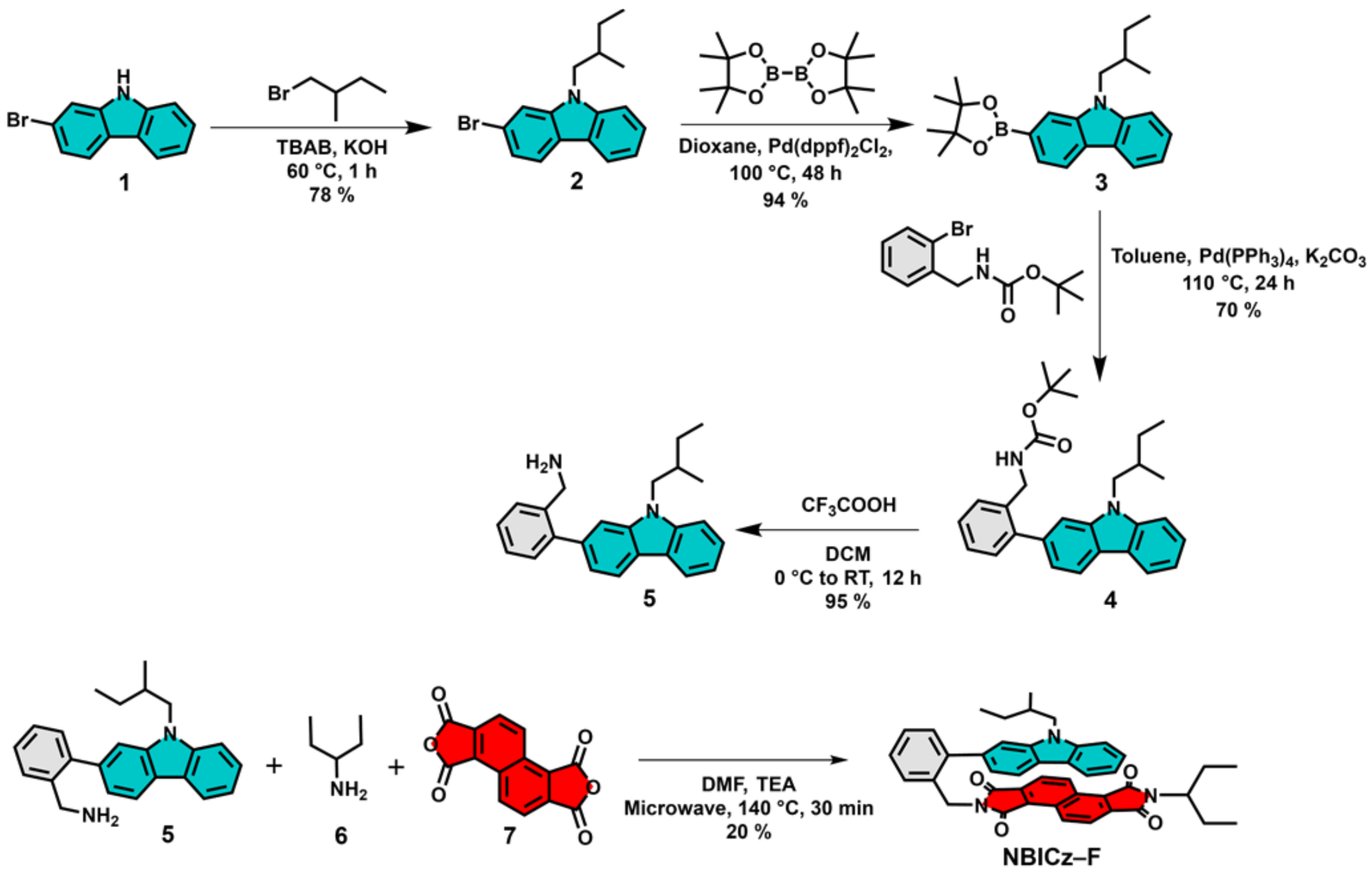

Scheme S1. Reaction pathway for the synthesis of **NBICz–F**.

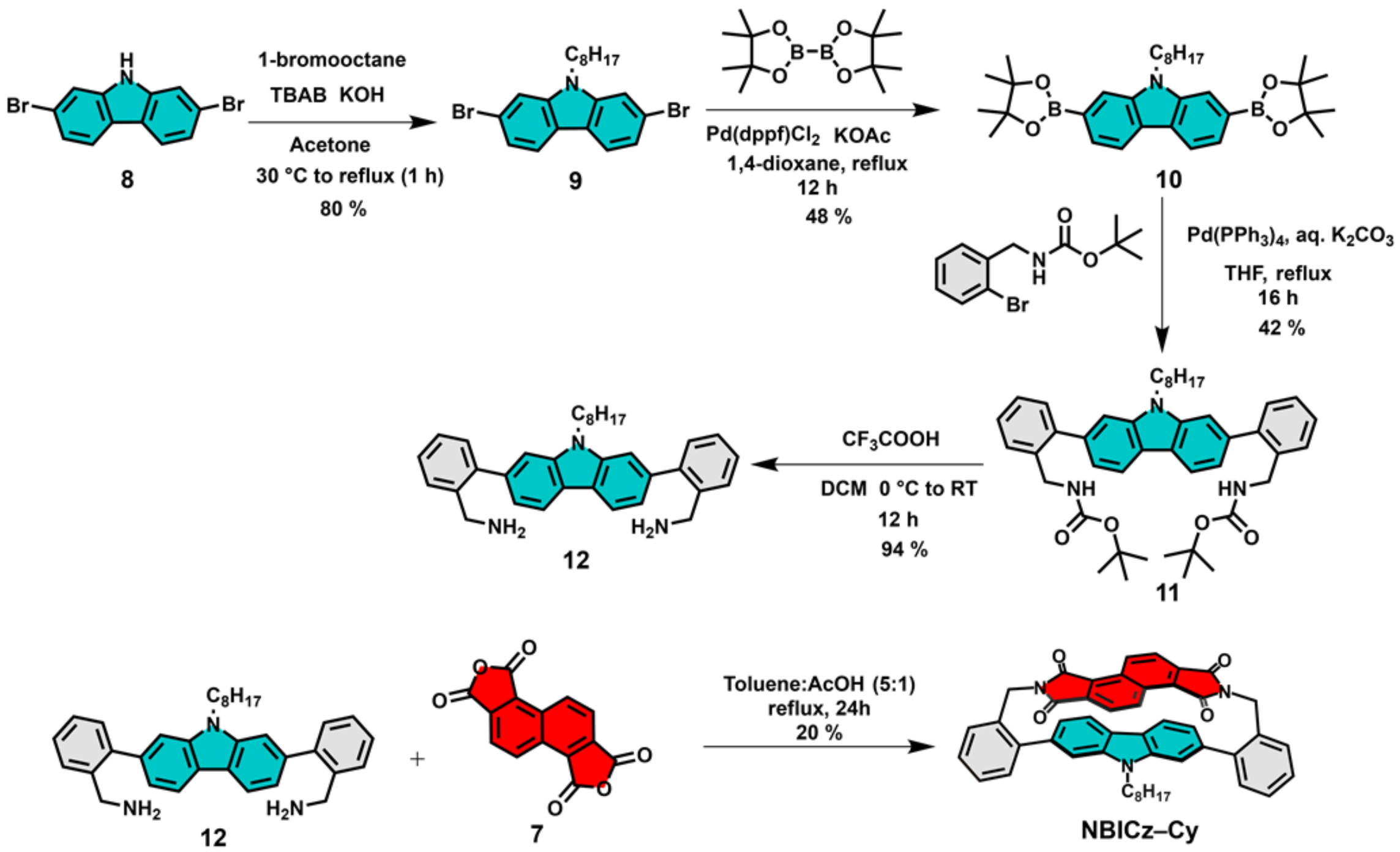

Scheme S2. Reaction pathway for the synthesis of **NBICz–Cy** hetero-cyclophane.

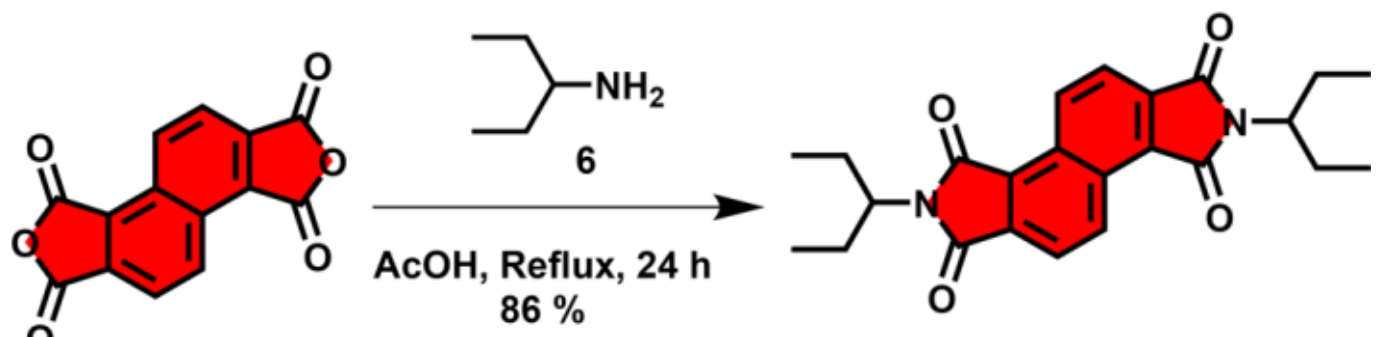

7 1,2:5,6–NBI-Ref

Scheme S3. Reaction pathway for the synthesis of **1,2:5,6–NBI-Ref**.

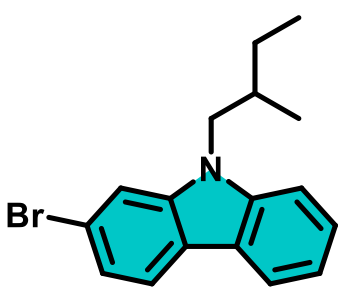

Compound 2: 2-Bromocarbazole (compound **1**) (2 g, 8.13 mmol), *tetra*-butyl ammonium bromide (262 mg, 812.7 µmol), and potassium hydroxide (1.82 g, 32.51 mmol) were taken in a single-necked 100 mL round-bottom flask and dissolved in acetone. The mixture was stirred for 1 hour at 30 °C. Then (S)-1-bromo-2-methylbutane (1.35 g, 12.19 mmol) was added dropwise to the solution. The reaction mixture was refluxed for 2 hours, and the solvent was removed under reduced pressure. The product was dissolved in DCM and decanted, and the remaining solid was disposed of. Then, column chromatography was performed using 25 % DCM in *n*-hexane to get compound **2** as a transparent liquid.

Yield = 2 g (6.32 mmol, 78 %) of a transparent liquid.

^{1}H NMR (400 MHz, CD_2Cl_2, 295 K): *δ* (ppm) = 8.06 (dt, *J* = 8 Hz, 1H, ArH), 7.96 (d, *J* = 8.28 Hz, 1H, ArH), 7.57 (d, *J* = 1.6 Hz, 1H, ArH), 7.50-7.46 (m, 1H, ArH), 7.43-7.41 (m, 1H, ArH), 7.32 (dd, *J* = 8, 1.2 Hz, 1H, ArH), 7.24 (td, *J* = 7, 1.2 Hz, 1H, ArH), 4.18-4.00 (m, 2H, CH_2), 2.19-2.07 (m, 1H, CH), 1.51-1.21 (m, 2H, CH), 0.96-0.90 (m, 6H, 2CH_3).

^{13}C NMR (101 MHz, CD_2Cl_2, 295 K): *δ* (ppm) = 142.3, 141.6, 126.6, 122.6, 122.3,122.2, 121.9, 120.7, 119.8, 119.6, 112.7, 109.9, 50.1, 35.7, 28.0, 17.6, 11.8.

HRMS (APCI, positive, $CHCl_3$:MeCN = 1:1): *m/z* calculated for $C_{17}H_{18}BrN^+$ $[M]^+$: 315.06226, found: 315.06291.

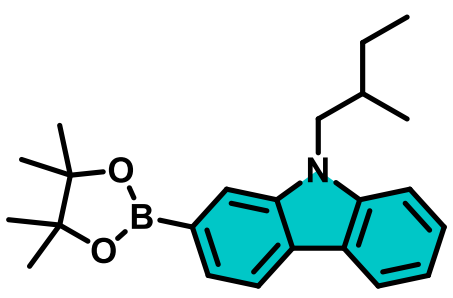

Compound 3: In a two-neck round-bottom flask, compound **2** (1 g, 3.16 mmol), bis(pinacolato)diborane (1,20 g, 4,74 mmol), potassium acetate (KOAc) (931.0 mg, 9.50 mmol) and [1,1′-bis(diphenylphosphino)ferrocene] dichloropalladium (II) (Pd(dppf)Cl_2) (23.65 mg, 31.62 µmol), were dissolved in 1,4-dioxane (30 mL) under nitrogen atmosphere and purged for 20 minutes. The reaction mixture was heated under reflux for 24 hours under a nitrogen atmosphere. After cooling, the solvent was evaporated under reduced pressure. The crude product was dissolved in DCM and extracted with distilled water and brine solution. Compound **3** was then purified by column chromatography (silica gel) using a 1:1 mixture of DCM: cyclohexane as the eluent.

Yield = 1.08 g (2.97 mmol, 94 %) of a white solid.

^{1}H NMR (400 MHz, CD_2Cl_2, 295 K): *δ* (ppm) = 8.12-8.08 (m, 2H, ArH), 7.85 (s, 1H, ArH), 7.60 (dd, *J* = 1 Hz, 8 Hz, 1H, ArH), 7.50-7.42 (m, 2H, ArH) 7.24 -7.19 (m ,1H,ArH), 4.24-4.13 (m, 2H, CH_2), 2.23-2.14 (m, 1H, CH), 1.50-1.40 (m, 1H, CH), 1.37(s, 12H, CH_3) 1.33-1.22 (m, 2H, CH_2), 0.96-0.91 (m, 6H, 2CH_3).

^{13}C NMR (101 MHz, CD_2Cl_2, 295 K): *δ* (ppm) = 141.9, 141.0, 126.7, 125.6, 125.3,122.9, 121.1, 119.9, 119.2, 115.9, 109.9, 85.3, 49.9, 35.7, 27.9, 25.3, 25.3, 17.6, 11.8.

HRMS (ESI, negative, $CHCl_3$:MeCN = 1:1): *m/z* calculated for $C_{29}H_{34}ClN_2O_2^-$ $[M+Cl]^-$: 477.23143, found 477.23003.

Melting point: 179-181 °C.

Compound 4: In a 250 mL Schlenk tube, compound **3** (845 mg, 2.95 mmol) was taken, and 30 mL of toluene was

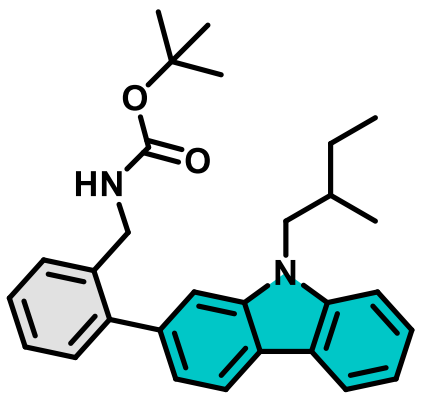

added. The mixture was then degassed with nitrogen for 10 minutes. Tetrakis(triphenylphosphine)palladium(0) ($Pd(PPh_3)_4$) (15.5 mg, 13.42 µmol) and 9-(2-methylbutyl)-9*H*-carbazole-2-boronic acid pinacol ester (1.04 g, 2.86 mmol) were added to the mixture. After 15 minutes of nitrogen purging, K_2CO_3 (1.12 g, 8.05 mmol) (dissolved in a degassed water and ethanol solution) was added and refluxed for 24 hours, then the solvent was evaporated under reduced pressure. The residue was redissolved in DCM (60 mL) and washed consecutively with a brine solution. The crude product was purified using column chromatography, eluted with 50 % DCM in hexane to yield compound **4**.

Yield = 835 mg (1.89 mmol; 70 %) of highly viscous, brown-coloured, transparent liquid.

^{1}H NMR (400 MHz, CD_2Cl_2, 295 K): *δ* (ppm) = 8.14-8.11 (m, 2H, ArH), 7.50-7.35 (m, 7H, ArH), 7.26-7.22 (m, 1H, ArH), 7.16 (dd, *J* = 6.4 Hz, 1.3 Hz, 1H, ArH), 4.75 (bs, 1H, NH), 4.29 (d, *J* = 6 Hz, 1H, CH) 4.25-4.13 (m, 1H, CH_2), 2.22-2.10 (m, 1H, CH_2), 1.52-1.42 (m, 1H, CH_2), 1.37 (s, 7H,CH_3), 1.30-1.25 (m,1H,CH), 0.95-0.90 (m, 6H, 2CH_3).

^{13}C NMR (101 MHz, CD_2Cl_2, 295 K): *δ* (ppm) = 156.2, 143.0, 141.8, 141.4, 138.9, 137.4, 131.0, 128.6, 128.0, 127.6, 126.2, 123.0, 1220.2, 120.7, 120.5, 120.4, 119.4, 110.3,109.8, 50.0, 43.1, 35.8, 28.6, 28.0, 17.7, 11.8.

HRMS (ESI, positive, $CHCl_3$:MeCN = 1:1): *m/z* calculated for $C_{29}H_{34}N_2NaO_2^+$ $[M+Na]^+$: 465.25125, found: 465.25426.

Compound 5: Compound **4** was dissolved in 20 mL of DCM in a 100 mL round-bottom flask and then placed in an

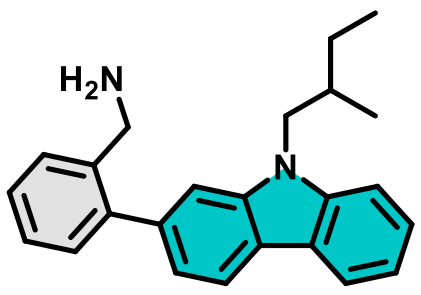

ice bath at 0 ˚C. Then, 6 mL of trifluoroacetic acid was added dropwise to the solution. After 15 minutes, the ice bath was removed, and the reaction was allowed to stir at room temperature for 12 hours. After 12 hours, thin-layer chromatography (TLC) was performed to verify the completion of the reaction. Solvent was then removed under reduced pressure to obtain a light brown liquid. Then the reaction mixture was poured into an aqueous saturated $NaHCO_3$ solution to quench the reaction. The crude material was extracted with DCM, and the organic phase was collected and dried over anhydrous $MgSO_4$. The solvent was then removed under reduced pressure, giving compound **5** as a colourless liquid, which was used without any further purification.

Yield = 1.47 g (5.14 mmol; 95 %) of a light brown liquid.

HRMS (ESI, positive, $CHCl_3$:MeCN = 1:1): *m/z* calculated for $C_{24}H_{27}N_2H^+$ $[M+H]^+$: 343.21688, found: 343.21777.

NBICz–F: In a pressure-tight 10-mL microwave vial, compound **5** (40 mg, 117 µmol) and compound **7** (naphtho[1,2-

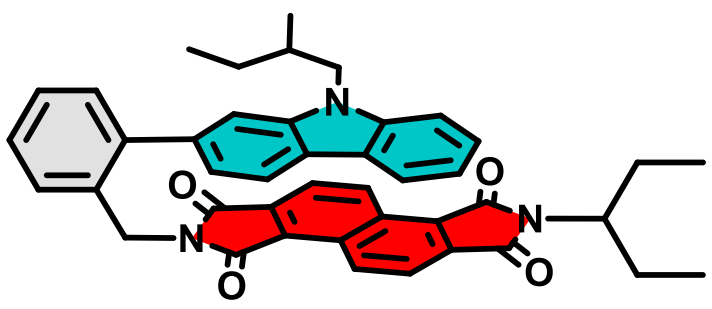

c:5,6-*c'*]difuran-1,3,6,8-tetraone) (31:30 mg, 117 µmol) were suspended in 5 mL of *N,N*-Dimethylformamide. To this suspension, 0.1 mL of dry triethylamine was added, and the mixture was sonicated until homogeneous. The reaction mixture was heated for 15 min at 140 °C under microwave irradiation.[S6] After cooling, pentan-3-amine (compound **6**, 10.2 mg, 117 µmol) and an additional 0.1 mL of dry triethyl amine were added. The reaction mixture was heated for an additional 15 min at 140 °C under microwave irradiation. After completion of the reaction, the solvent was removed under reduced pressure to obtain the crude product, which was purified by column chromatography using 10% ethyl acetate in *n*-hexane as eluent to obtain compound **NBICz–F** as an orange-yellow solid. **NBICz–F** was further purified by gel permeation chromatography (GPC) at a flow rate of 6 mL/min using chloroform as eluent.

Yield = 15 mg (23 µmol, 20 %) of orange-yellow solid.

^{1}H NMR (400 MHz, CD_2Cl_2, 295 K): *δ* (ppm) = 9.12 (dd, *J* = 8.4, 0.9 Hz, 1H, ArH), 8.93 (dd, *J* = 8.5, 0.9 Hz, 1H, ArH), 7.87 (d, *J* = 8.5, 1H, ArH), 7.84 (dd, *J* = 8, 0.5 Hz, 1H, ArH), 7.83-7.78 (m, 2H, ArH), 7.41-7.32 (m, 5H, ArH), 7.41-7.32 (m, 5H, ArH), 7.28 (dd, *J* = 0.8, 0.5 Hz, 1H, ArH), 7.24-7.23 (m, 1H, ArH), 7.1-7.0 (m, 2H, ArH), 5.02 (d, *J* = 1.6, 2H, CH), 4.1-3.9 (m, 3H, CH), 2.1-1.8 (m, 5H, CH), 1.49-1.38 (m, 1H, CH), 1.28-1.17 (m, 1H, CH), 0.95-0.85 (m, 12H, CH).

^{13}C NMR (101 MHz, CD_2Cl_2, 295 K): *δ* (ppm) = 133.0, 131.8, 131.6, 131.5, 130.9, 130.7, 128.6, 128.1, 127.8, 127.7, 127.3, 126.0, 122.6, 122.0, 121.7, 121.6, 120.5, 120.4, 119.2, 110.1, 109.5, 56.3, 49.8, 41.1, 35.7, 28.0, 26.0, 17.6, 11.8, 11.6.

HRMS (MALDI-TOF, negative mode, DCTB in chloroform): *(m/z)* $[M]^{-\cdot}$, calculated for $C_{43}H_{39}N_3O_4$: 661.29406, found: 661.40157.

Melting point: 116-119 °C.

Compound 9: 2,7-dibromo *9H*-carbazole (compound **8**) (5 g, 15.4 mmol), *tetra*-butyl ammonium bromide (495.9 mg, 1.54 mmol), and potassium hydroxide (3.45 g, 61.54 mmol) were added in a single-necked 100 mL round-bottom flask and dissolved in acetone (10 mL). The solution was stirred for 40 minutes at 30 °C. Then 1-bromooctane (5.94 g, 30.77 mmol) was added dropwise to the solution. Further, the reaction mixture was refluxed for 1 hour. Then the solvent was removed under reduced pressure to obtain the crude product, which was purified by column chromatography (silica gel) using hexane as eluent to obtain compound **9** as a white solid.

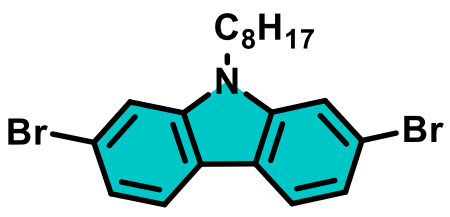

Yield = 5.4 g (12.30 mmol, 84 %) of white solid.

^{1}H NMR (400 MHz, $CDCl_3$, 295 K): *δ* (ppm) = 7.89 (d, *J* = 8 Hz, 2H, ArH), 7.53 (d, 2H, ArH), 7.34 (dd, *J* = 8 Hz, 2H, ArH), 4.19 (t, *J* = 8 Hz, 2H, CH_2), 1.84 (qui, *J* = 7 Hz, 2H, CH_2), 1.38-1.25 (m, 10 H, CH_2), 0.87 (t, *J* = 6 Hz, 3H, CH_3).

Melting point: 73-76 °C.

^{13}C NMR (101 MHz, $CDCl_3$, 295 K): *δ* (ppm) = 141.4, 122.5, 121.5, 121.3, 119.7, 112.0, 43.4, 31.8, 29.3, 29.2, 28.8, 27.2, 22.6, 14.1.

HRMS (APCI, positive, $CHCl_3$:MeCN = 1:1): *m/z* calculated for $C_{20}H_{23}Br_2N^+$ $[M]^+$: 435.01972, found: 435.01917.

Compound 10: In a round-bottom flask, compound **9** (1.5 g, 3.43 mmol), bis(pinacolato)diborane (1.92 g, 7.55 mmol), KOAc (1.68 g, 17.15 mmol) and Pd(dppf)Cl_2 (140 mg, 171.54 µmol) were dissolved in 1,4-dioxane (50 mL) under nitrogen atmosphere. The reaction mixture was kept in reflux conditions for 24 h. Upon completion of the reaction, the reaction mixture was cooled to ambient temperature. The solvent was evaporated under reduced pressure and dissolved in dichloromethane. The organic mixture was washed with distilled water and brine solution three times. The organic fraction was evaporated under reduced pressure to obtain the crude product, which was further purified by column chromatography using DCM as eluent. The pure compound **10** was obtained as an off-white solid.

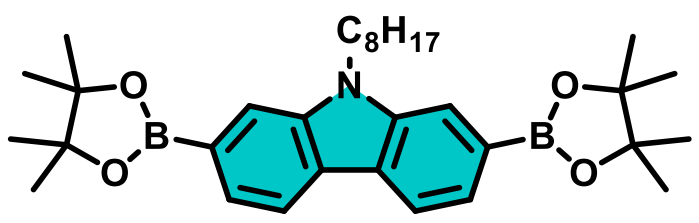

Yield = 880 mg (1.65 mmol, 48 %) of off-white solid.

^{1}H NMR (400 MHz, $CDCl_3$, 295 K): *δ* (ppm) = 8.12 (d, *J* = 8 Hz, 2H, ArH), 7.88 (s, 2H, ArH), 7.68 (d, *J* = 8 Hz, 2H, ArH), 4.38 (t, *J* = 8 Hz, 2H, CH_2), 1.89 (qui, *J* = 7 Hz, 2H, CH_2), 1.40 (s, 24H, CH_2), 1.37-1.24 (m, 10H, CH_2), 0.87 (t, *J* = 8 Hz, 3H, CH_3).

^{13}C NMR (101 MHz, $CDCl_3$, 295 K): *δ* (ppm) = 140.4, 125.1, 124.8, 120.0, 115.3, 83.8, 42.9, 31.8, 29.4, 29.2, 29.18, 27.1, 24.9, 22.65, 14.1.

HRMS (ESI, positive, $CHCl_3$:MeCN = 1:1): *m/z* calculated for $C_{32}H_{47}B_2NNaO_4^+$ $[M+Na]^+$: 554.35889, found: 554.35834.

Melting point: 193-195 °C.

Compound 11: In a 250 mL two-neck round-bottom flask, *N*-Boc-2-Brombenzylamine (500 mg, 1.75 mmol) was added, followed by 100 mL dry THF and by degassing with nitrogen for 10 minutes. $Pd(PPh_3)_4$ (80.7 mg, 69.8 µmol) and compound **10** (371.3 mg, 698.8 µmol) were added to the mixture. After 15 minutes of nitrogen purging, K_2CO_3 (386.3 mg, 2.8 mmol) was added and kept in reflux condition for 24 hours. Upon completion of the reaction, the solvent was evaporated under reduced pressure and dissolved in DCM. This was followed by extraction with brine solution. Then the organic fraction was evaporated under reduced pressure to obtain the crude product. This was further purified by column chromatography using ethyl acetate: hexane (20:80) as eluent to obtain compound **11** as a white-coloured solid. Compound **11** was further purified by gel permeation chromatography (GPC) at a flow rate of 6 mL/min using chloroform as eluent.

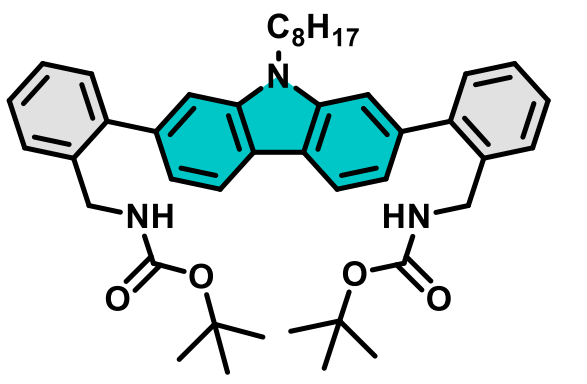

Yield = 202 mg (292.77 µmol, 42 %) of white solid.

^{1}H NMR (400 MHz, CD_2Cl_2, 295 K): *δ* (ppm) = 8.15 (d, *J* = 8 Hz, 2H, ArH), 7.49-7.46 (m, 2H, ArH), 7.42-7.34 (m, 10H, ArH), 7.19 (dd, *J* = 8 Hz, 2H, ArH), 4.79-4.76 (t, *J* = 6 Hz, 2H, CH_2), 4.34-4.30 (m, 4H, CH_2), 1.87 (qui, *J* = 8 Hz, 2H, CH_2), 1.38 (s, 18H, CH_3), 1.30-1.20 (m, 10 H, CH_2), 0.82 (t, *J* = 8 Hz, 3H, CH_3).

^{13}C NMR (101 MHz, CD_2Cl_2, 295 K): *δ* (ppm) = 155.7, 142.4, 140.9, 138.5, 136.9, 130.4, 128.0, 127.5, 127.0, 121.6, 120.30, 120.0, 109.5, 79.0, 43.2, 42.5, 31.8, 29.4, 29.2, 29.0, 28.1, 27.4, 22.6, 13.8.

HRMS (ESI, negative, $CHCl_3$:MeCN = 1:1): *m/z* calculated for $C_{44}H_{55}ClN_3O_4^-$ $[M+Cl]^-$: 724.38811, found: 724.38548.

Melting point: 130-132 °C.

Compound 12: Compound **11** (180 mg, 260.89 µmol) was added to a 100 mL round-bottom flask and dissolved in 20 mL DCM, and placed in an ice bath at 0 °C. Then 6 mL CF_3COOH was added dropwise to the solution. After 15 minutes, the ice bath was removed, and the reaction was allowed to stir at RT for 12 hours. Upon completion of the reaction, the reaction mixture was poured into aqueous saturated $NaHCO_3$ to quench the reaction. The crude material was extracted with DCM, and the organic phase was collected and dried with anhydrous Na_2SO_4. Removal of the solvent under reduced pressure gave compound **12** as an off-white viscous solid (120 mg, 245.04 µmol, 94 %), which was used as such without any further purification.

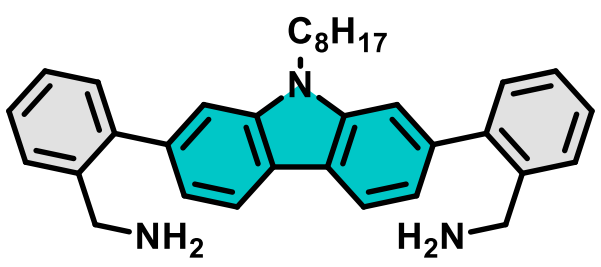

HRMS (ESI, positive, $CHCl_3$:MeCN = 1:1): *m/z* calculated for $C_{34}H_{40}N_3^+$ $[M+H]^+$: 490.32222, found: 490.32167.

NBICz–Cy: In an oven-dried 1000 mL round-bottom flask, compound **12** (30 mg, 61.26 µmol), compound **7** (naphtho[1,2-*c*:5,6-*c'*]difuran-1,3,6,8-tetraone) (16.43 mg, 61.26 µmol) were suspended in 600 mL toluene: acetic acid (5:1) mixture and kept in reflux condition for 24 hours. After completion of the reaction, the solvent was removed under reduced pressure to obtain the crude product, which was purified by column chromatography using DCM as eluent to obtain **NBICz–Cy** as an orange-yellow solid. **NBICz–Cy** was further purified by gel permeation chromatography (GPC) at a flow rate of 6 mL/min using chloroform as eluent.

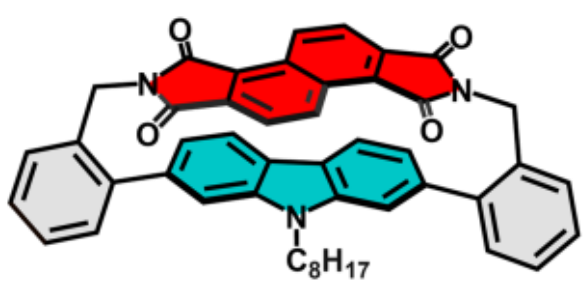

Yield = 8.9 mg (12 µmol, 20 %) of orange-yellow solid.

^{1}H NMR (400 MHz, CD_2Cl_2, 295 K): *δ* (ppm) = 8.81 (dd, *J* = 16, 8 Hz, 2H, ArH), 7.79 (dd, *J* = 24, 8 Hz, 2H, ArH), 7.54-7.35 (m, 6H, ArH), 7.29-7.26 (m, 2H, ArH), 7.14 (dd, *J* = 8 Hz, 1H, ArH), 7.06 (d, J = 12 Hz, 1H, ArH), 6.91-6.87 (m, 2H, ArH), 6.72 (dd, *J* = 8 Hz, 1H, ArH), 6.62 (s, 1H, ArH), 5.74 (d, *J* = 16H, 1H, CH), 5.25 (d, *J* = 4 Hz, 2H, CH_2), 4.57 (d, *J* = 16H, 1H, CH), 3.95 (qui, *J* = 7 Hz, 1H, CH), 3.72 (qui, *J* = 8 Hz, 1H, CH), 1.60 (qui, *J* = 6 Hz, 2H, CH_2), 1.24-1.19 (m, 10H, CH_2), 0.83 (t, *J* = 8Hz, 3H, CH_3).

^{13}C NMR (101 MHz, CD_2Cl_2, 295 K): *δ* (ppm) = 168.5, 168.4, 167.6, 167.3, 140.8, 139.7, 139.0, 138.6, 138.5, 133.4, 133.1, 132.7, 132.3, 132.1, 132.0, 131.2, 131.0, 129.9, 129.8, 129.1, 127.6, 127.6, 127.5, 127.3, 126.4, 126.0, 125.3, 121.5, 120.8, 120.4, 120.1, 119.9, 119.8, 119.7, 119.1, 109.2, 107.2, 43.0, 42.7, 40.1, 31.7, 29.2, 29.2, 29.0, 27.0, 22.6, 13.8.

HRMS (MALDI-TOF, positive mode, DCTB in chloroform): *(m/z)* $[M]^{+\cdot}$, calculated for $C_{48}H_{39}N_3O_4$: 721.29406, found: 721.34465.

Melting point: 308-310 °C (for the racemic compound); 308-310 °C (for *(R)*-**NBICz–Cy**); 307-309 °C (for *(S)*-**NBICz–Cy**).

Synthesis of 1,2:5,6–NBI-Ref: In a single-necked 100 mL round-bottom flask, pentan-3-amine (compound **6**, 195.02 mg, 2.24 mmol, 3.00 eq.) and naphtho[1,2-*c*:5,6-*c'*]difuran-1,3,6,8-tetraone (compound **7**) (200 mg, 745.77 µmol, 1.00 eq.) were suspended in 30 mL acetic acid and heated under reflux for 24 hours. After cooling to room temperature, the reaction mixture was poured into water, which led to precipitation. After filtration, the solid was then purified by column chromatography (silica gel) using dichloromethane as an eluent.

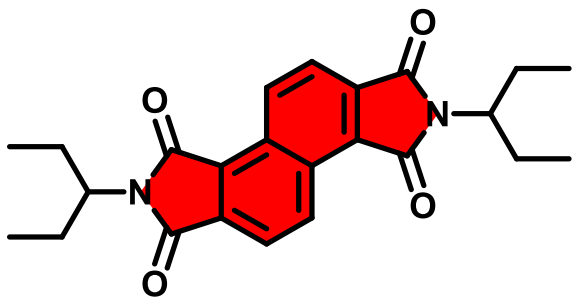

Yield = 261 mg (642.09 µmol, 86 %) of white solid.

^{1}H NMR (400 MHz, CD_2Cl_2, 295 K): *δ* (ppm) = 9.35 (d, *J* = 8 Hz, 2H, ArH), 8.07 (d, *J* = 8 Hz, 2H, ArH), 4.11-4.04 (m, 2H, CH), 2.14-2.02 (m, 4H, CH_2), 1.88-1.77 (m, 4H, CH_2), 0.90 (t, *J* = 8Hz, 3H, CH_3).

^{13}C NMR (101 MHz, CD_2Cl_2, 295 K): *δ* (ppm) = 169.86, 168.93, 133.17, 131.99, 131.20, 127.69, 121.90, 56.26, 25.79, 11.40.

HRMS (MALDI-TOF, negative mode, DCTB in chloroform): *(m/z)* $[M]^{-\cdot}$, calculated for $C_{48}H_{39}N_3O_4$: 406.18926, found: 406.23233.

Melting point: 205-207 °C.

3. Crystallographic Analysis

The single crystals of **NBICz–Cy** were grown by slow vapour diffusion of methanol into the chloroform solution.

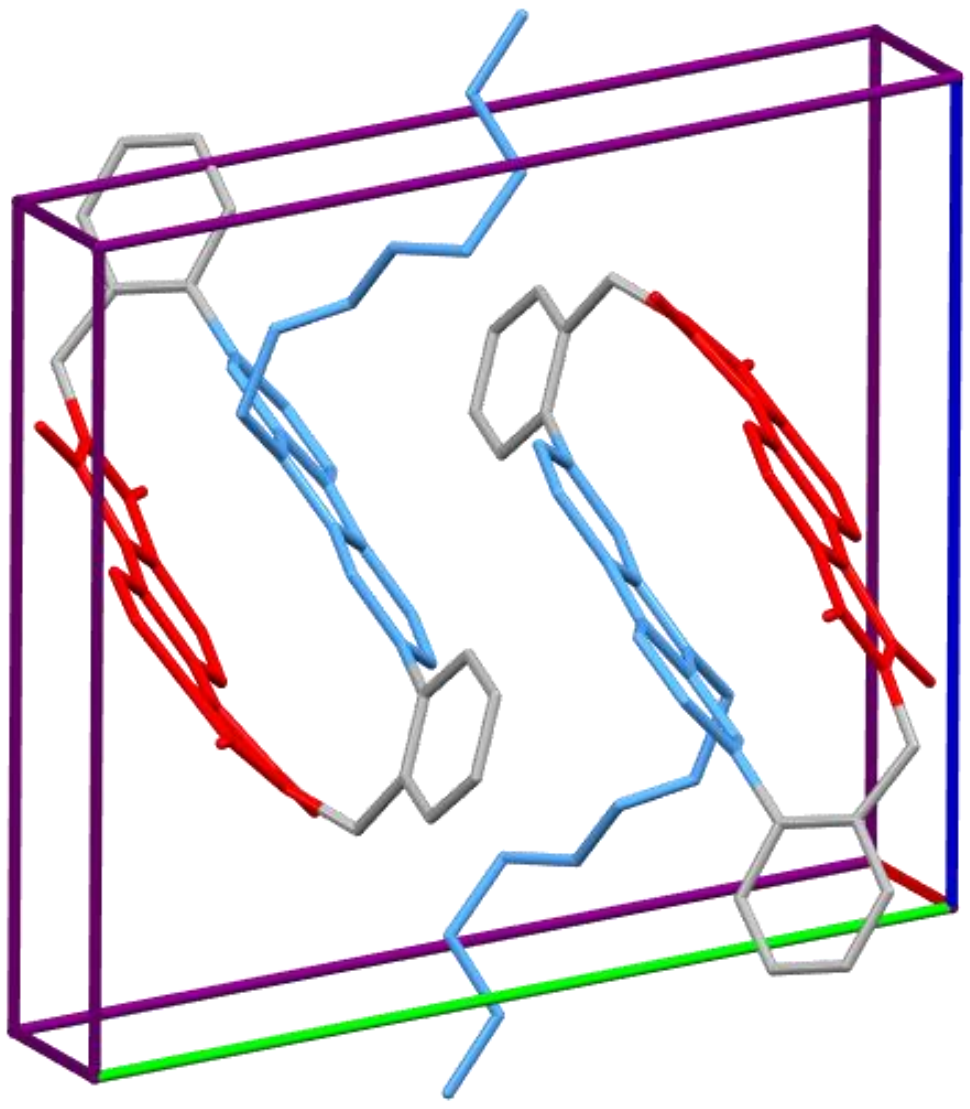

Figure S1. Unit cell in the molecular packing arrangement of **NBICz–Cy** according to single-crystal X-ray analysis.

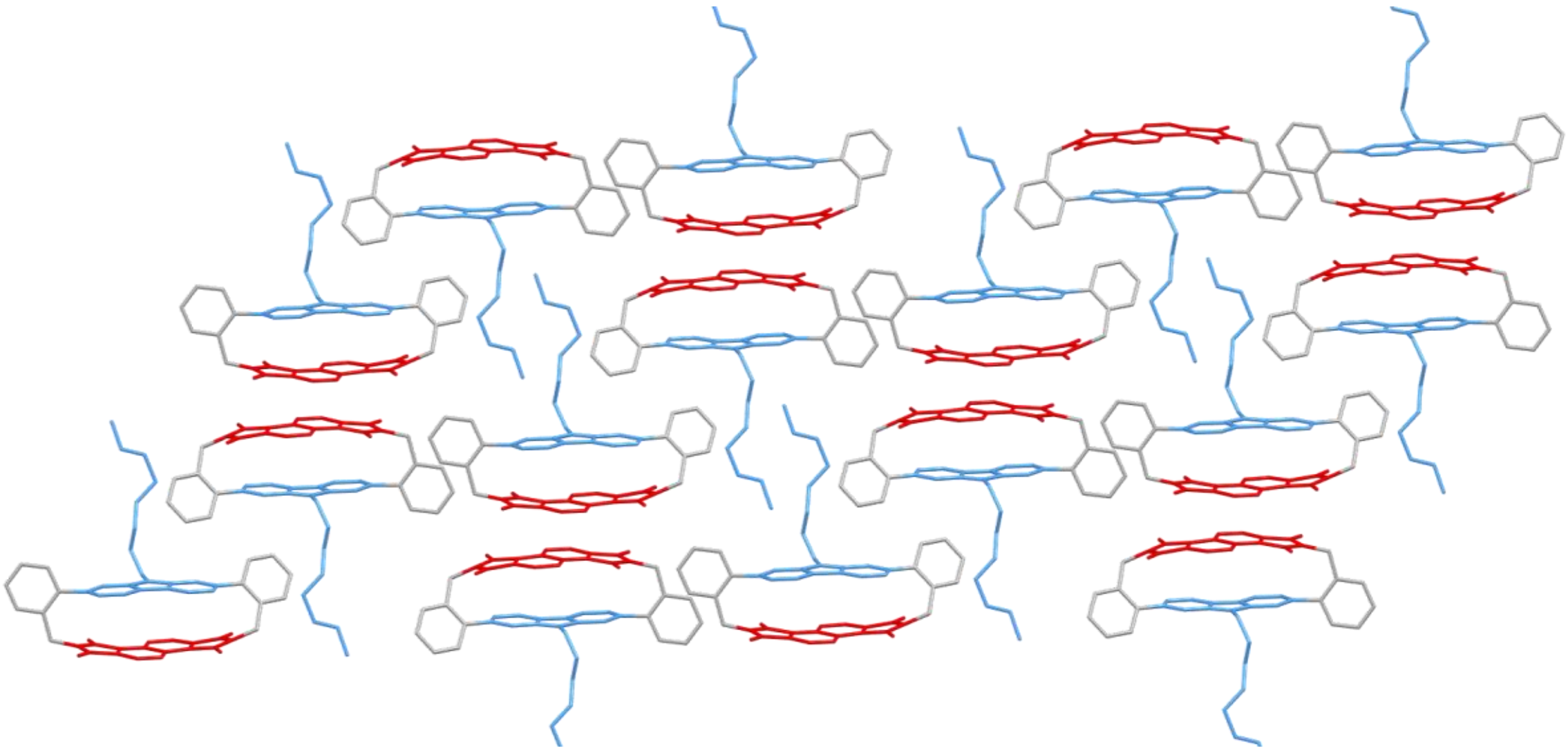

Figure S2. Extended packing of **NBICz–Cy** in the crystal lattice according to single-crystal X-ray analysis.

Table S1. Crystal data and structure refinement for **NBICz–Cy**.

CCDC Number	2531458	
Empirical formula	C48.81 H39.81 Cl2.42 N3 O4	
Formula weight	818.26	
Temperature	100(2) K	
Wavelength	0.61992 Å	
Crystal system	Triclinic	
Space group	P-1	
Unit cell dimensions	a = 10.006(2) Å	α = 97.31(3)°.
	b = 14.338(3) Å	β = 109.67(3)°.
	c = 14.769(3) Å	γ = 90.98(3)°.
Volume	1974.9(8) Å^3	
Z	2	
Density (calculated)	1.376 Mg/m^3	
Absorption coefficient	0.170 mm^{-1}	
F(000)	854	
Crystal size	0.050 x 0.050 x 0.020 mm^3	
Theta range for data collection	1.290 to 27.969°.	
Index ranges	-12<=h<=12, -18<=k<=18, -20<=l<=21	
Reflections collected	74135	
Independent reflections	10581 [R(int) = 0.0405]	
Completeness to theta = 21.836°	96.9 %	
Absorption correction	None	
Refinement method	Full-matrix least-squares on F^2	
Data / restraints / parameters	10581 / 1492 / 768	
Goodness-of-fit on F^2	1.045	
Final R indices [I>2sigma(I)]	R1 = 0.0853, wR2 = 0.2424	
R indices (all data)	R1 = 0.0867, wR2 = 0.2440	
Extinction coefficient	0.049(6)	
Largest diff. peak and hole	0.958 and -1.026 e.Å^{-3}	

4. Theoretical Calculations:

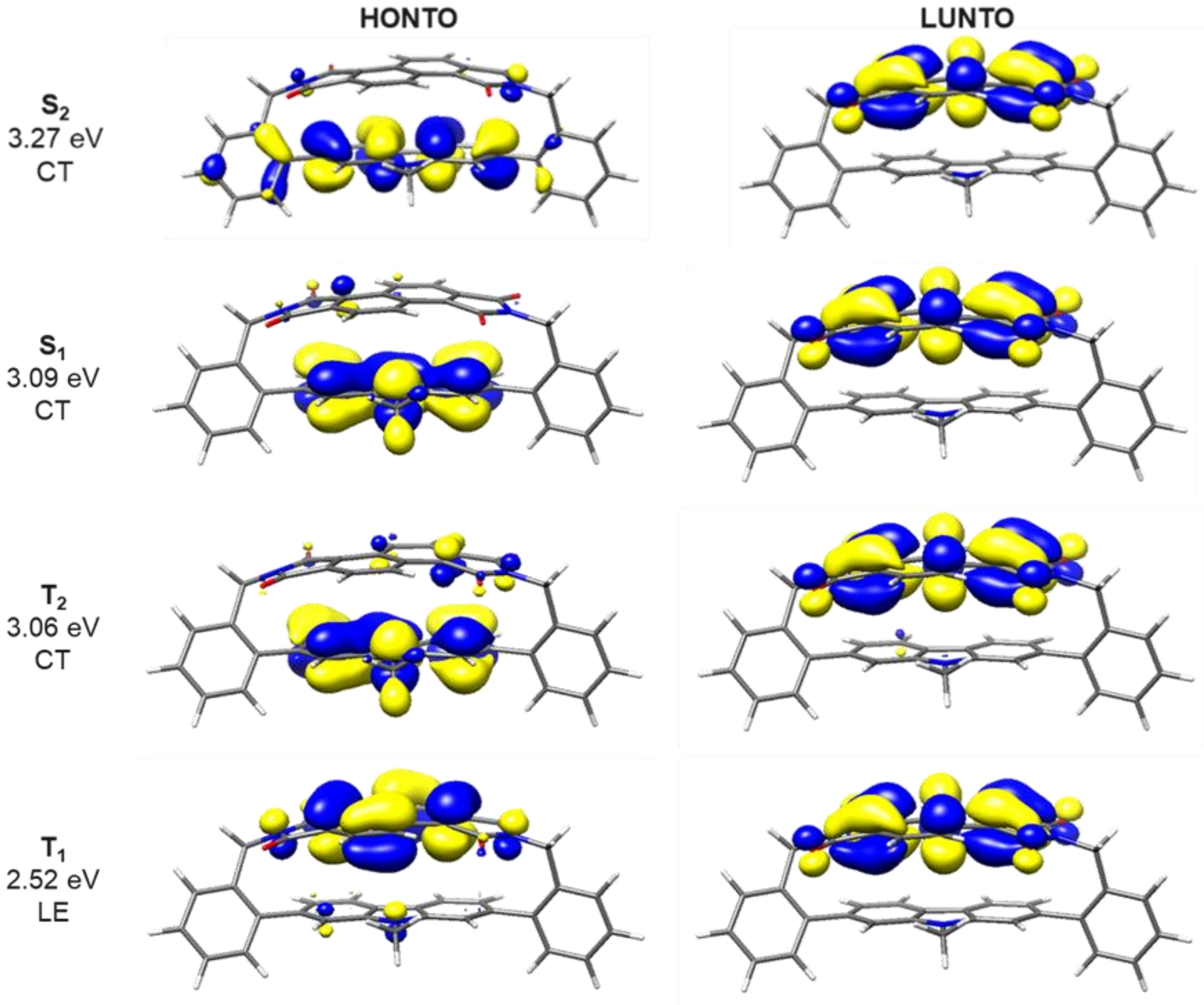

Figure S3. Natural transition orbitals (NTOs) of vertical excitations for S_1, S_2, T_1 and T_2 states for **NBICz–Cy** hetero-cyclophane (CAM-B3LYP-D3/def2-SVP optimized S_0 geometry and natural transition orbitals (NTOs) of vertical excitations from TD (S_1) and TDA (T_1) calculations using Gaussian16, all the calculations are done in the gas phase).

Note: The hole and electron densities are located over the donor and acceptor units, respectively, indicating the charge transfer (CT) nature of the lowest energy singlet state (S_1). The S_2 state is also CT in nature. Whereas, for the lowest energy triplet state (T_1), both hole and electron densities are located over the acceptor core, suggesting its locally excited (LE) character. The second triplet excited state (T_2) is also a CT state in nature (^{3}CT) and is isoenergetic with the ^{1}CT (S_1) state, allowing for efficient ISC and rISC. The ^{3}CT state is vibronically coupled with the lowest energy triplet state (^{3}LE).

5. Optical Properties:

Sample Preparation

Neat films of **NBICz–Cy** hetero-cyclophane were prepared from 4 mg/mL solutions of the respective derivatives in chloroform by spin coating over a clean quartz substrate. 1 wt. % compound: PMMA thin films were prepared by mixing 1 mg of compound in 100 mg of PMMA in 1 mL of chloroform. This mixture was then sonicated for 10–15 min to dissolve all the components thoroughly. The drop-cast PMMA doped film was dried at 60 °C for 30 min before proceeding with the photophysical experiments.

Measurement

Solution state measurements at 77 K were performed using a sample filled in an NMR tube and placed in a cold finger sample holder. For 77 K, liquid N_2 was added to the cold finger, and the spectra were measured. For the room–temperature measurement of the same sample, the same setup without liquid N_2 was used.

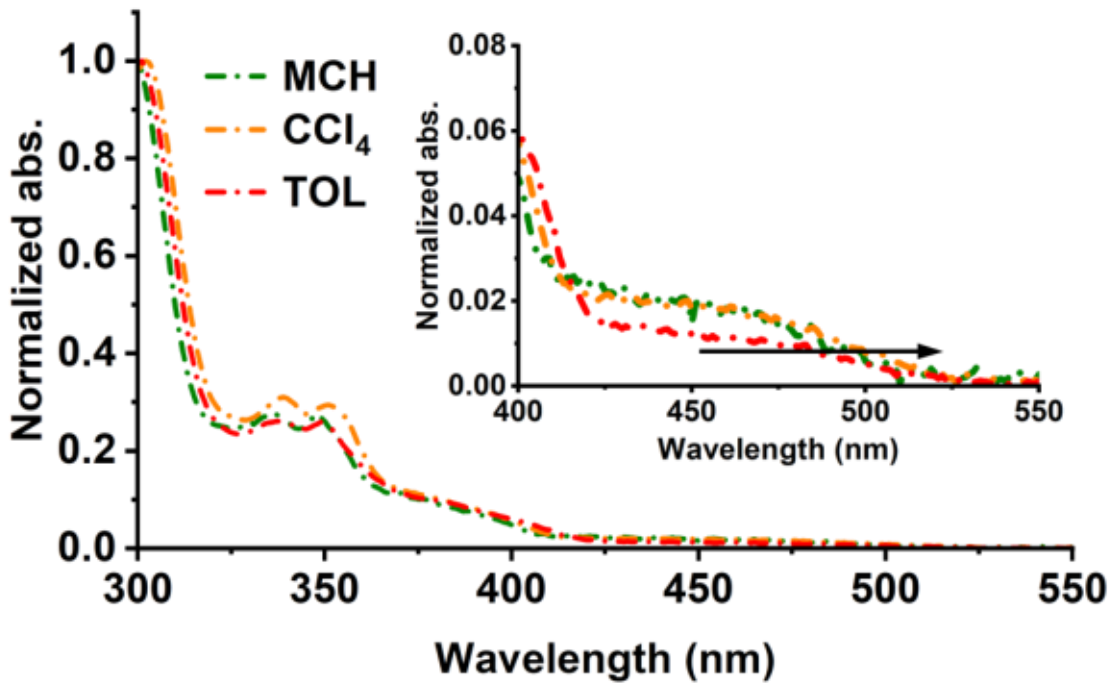

Figure S4. Normalized UV-Visible absorption spectra of **NBICz–F** in solvents of different polarities ([c] = 20 µM in both CCl_4 and TOL, respectively, T = 293 K). The inset depicts the magnified absorbance in the low-energy region.

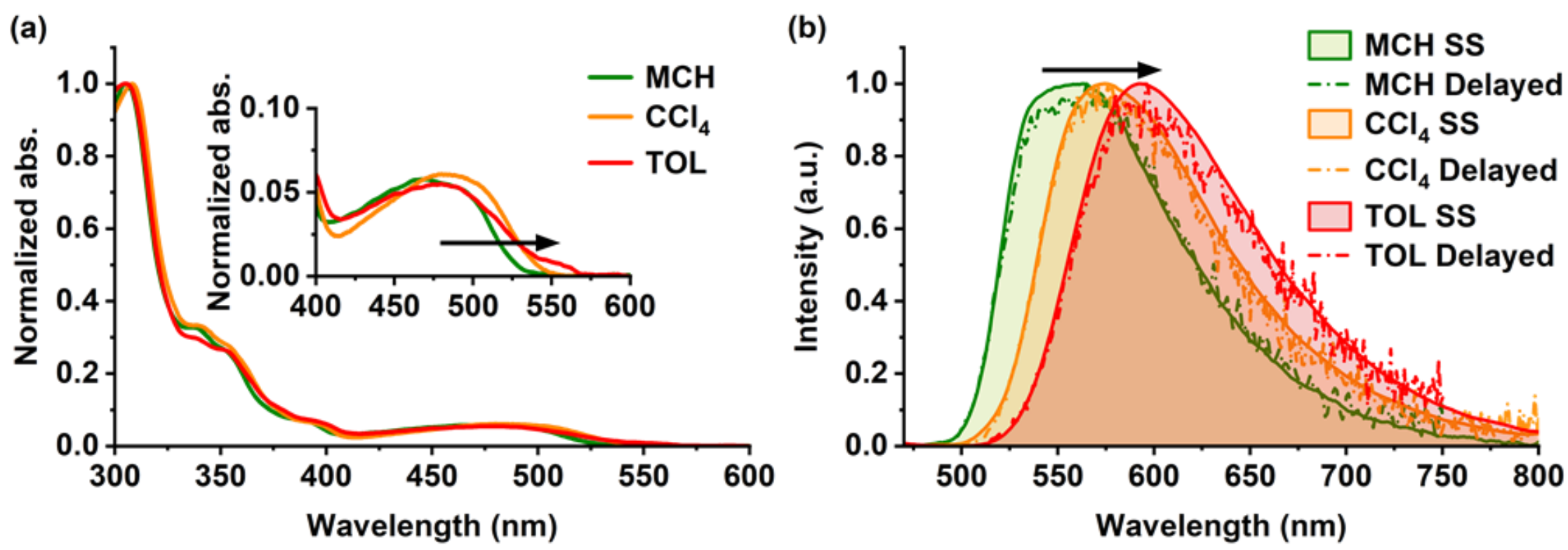

Figure S5. Normalized (a) UV-Visible absorption and (b) steady state and delayed fluorescence (delay time = 50 µs) spectra of **NBICz–Cy** in methyl cyclohexane, carbon tetrachloride and toluene solvents ([c] = 17.2, 11.8, and 10 µM in MCH, CCl_4 and TOL, respectively, T = 293 K). The inset of Figure (a) depicts the magnified absorbance in the low-energy region.

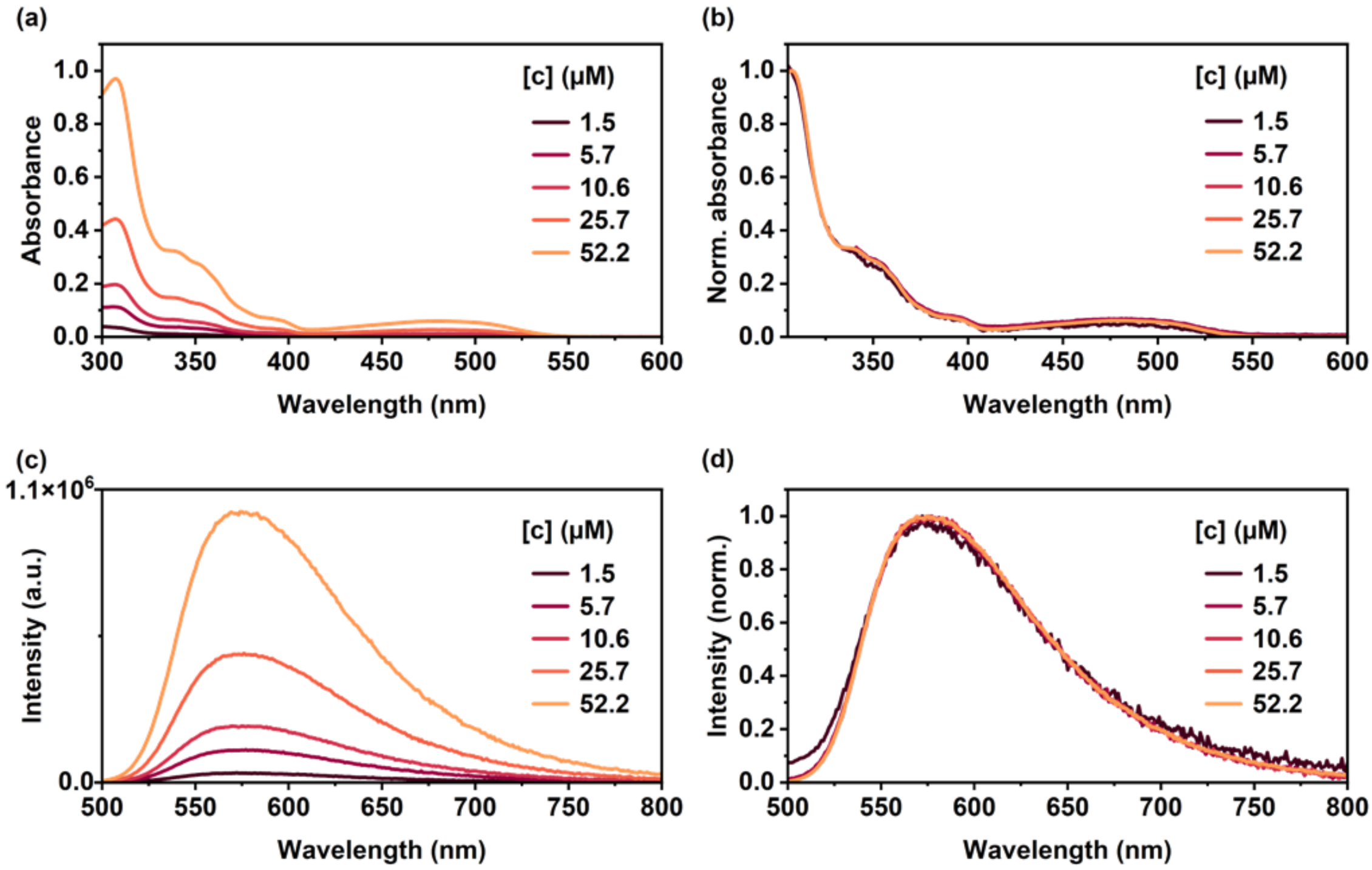

Figure S6. Concentration-dependent (a) UV-visible absorption, (c) steady-state emission spectra and normalized (b) UV-visible absorption and (d) emission spectra of **NBICz–Cy** in carbon tetrachloride (CCl_4) solution ($\lambda_{exc.}$ = 450 nm).

Note: The normalized absorption and emission spectra clearly show no change in the spectral feature with varying concentration, rules out the possibility of any aggregate formation.

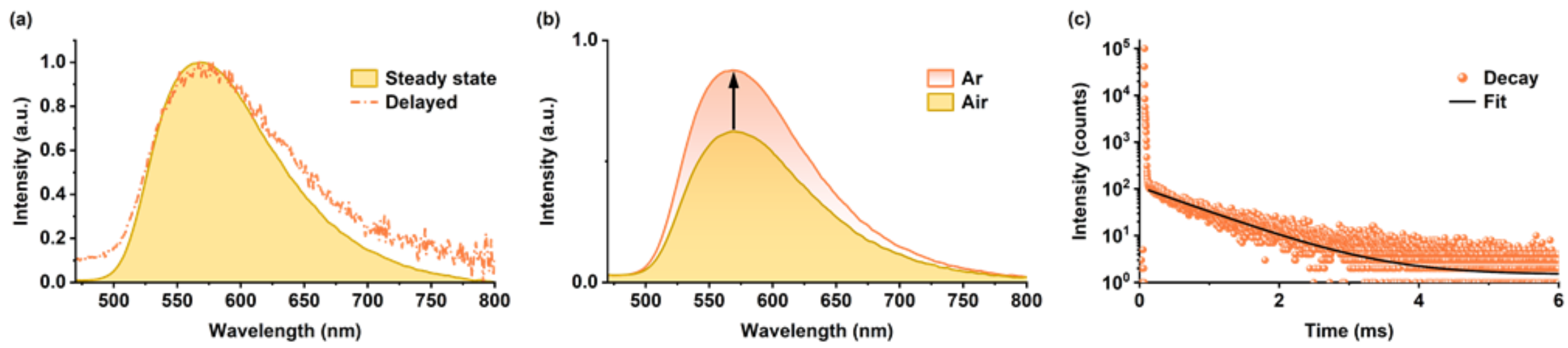

Figure S7. Delayed fluorescence of **NBICz–F** in carbon tetrachloride (CCl_4): (a) Normalized steady-state and delayed fluorescence spectra in argon atmosphere. (b) Steady-state emission spectra under air and argon atmosphere. (c) Delayed fluorescence lifetime decay profile under argon atmosphere ([c] = 20 μM, $\lambda_{exc.}$ = 400 nm, $\lambda_{collected}$ = 570 nm, delay time = 50 μs, T = 293 K).

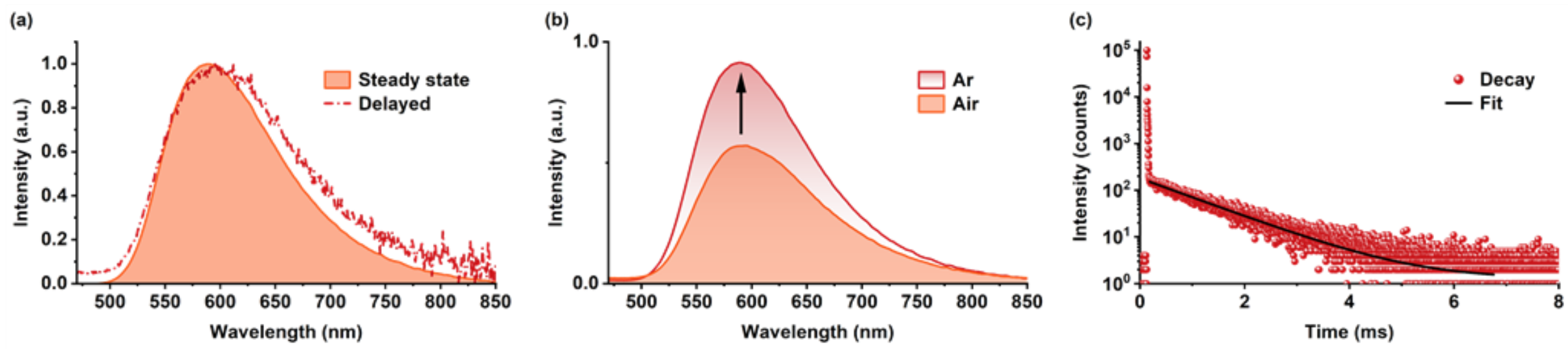

Figure S8. Delayed fluorescence of **NBICz–F** in toluene (TOL): (a) Normalized steady-state and delayed fluorescence spectra in argon atmosphere. (b) Steady-state emission spectra under air and argon atmosphere. (c) Delayed fluorescence lifetime decay profile under argon atmosphere ([c] = 20 μM, $\lambda_{exc.}$ = 400 nm, $\lambda_{collected}$ = 590 nm, delay time = 50 μs, T = 293 K).

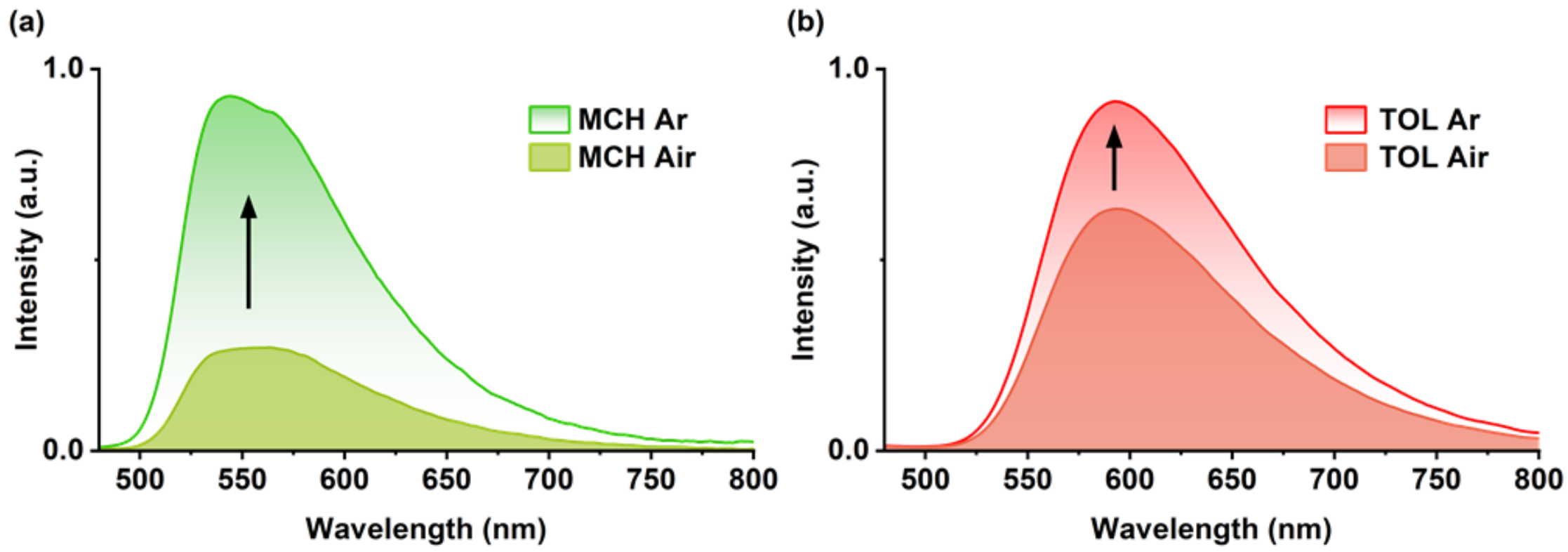

Figure S9. (a) Steady-state emission spectra of **NBICz–Cy** under air and argon atmosphere in (a) methyl cyclohexane and (b) toluene solvents. (T = 293 K, [c] = 17.2 and 10 μM for MCH and TOL, respectively, $\lambda_{exc.}$ = 450 nm in both cases).

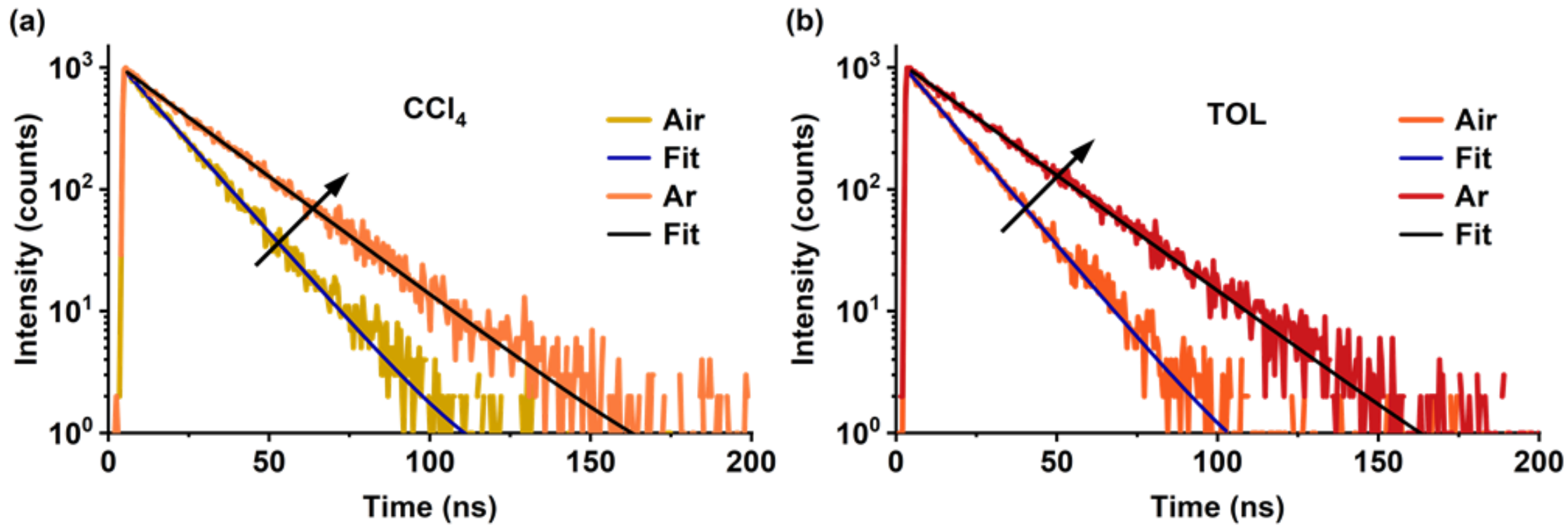

Figure S10. Prompt fluorescence lifetime decay profiles of **NBICz–F** under air and argon atmosphere in (a) carbon tetrachloride and (b) toluene solvents ([c] = 20 μM in CCl_4 and TOL, respectively; $\lambda_{exc.}$ = 400 nm, $\lambda_{collected}$ = 570 and 590 nm for CCl_4 and TOL, respectively, T = 293 K).

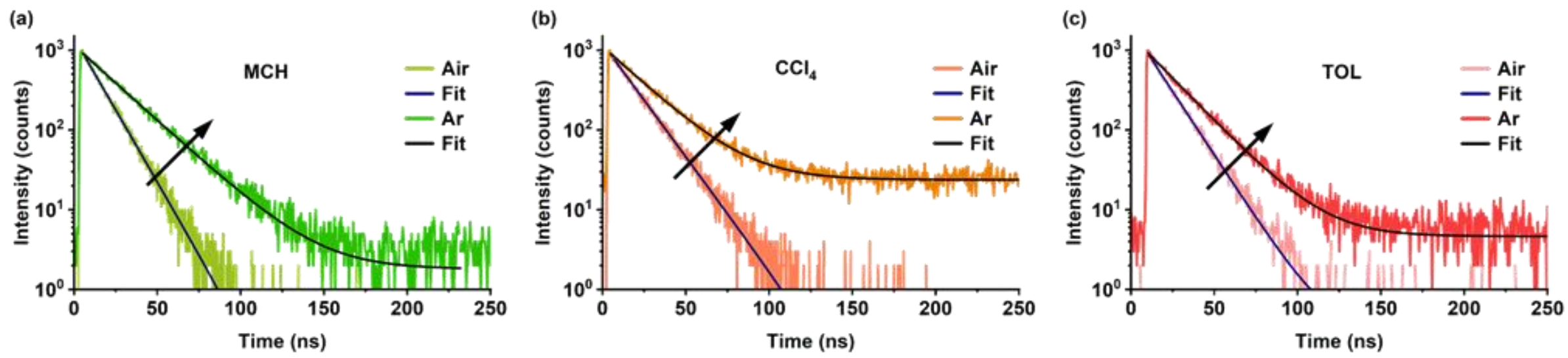

Figure S11. Prompt fluorescence lifetime decay profiles of **NBICz–Cy** under air and argon atmosphere in (a) methyl cyclohexane, (b) carbon tetrachloride and (c) toluene solvents ([c] = 17.2, 11.8, and 10 μM in MCH, CCl_4 and TOL, respectively; $\lambda_{exc.}$ = 450 nm, $\lambda_{collected}$ = 560, 575, and 590 nm for MCH, CCl_4 and TOL, respectively, T = 293 K).

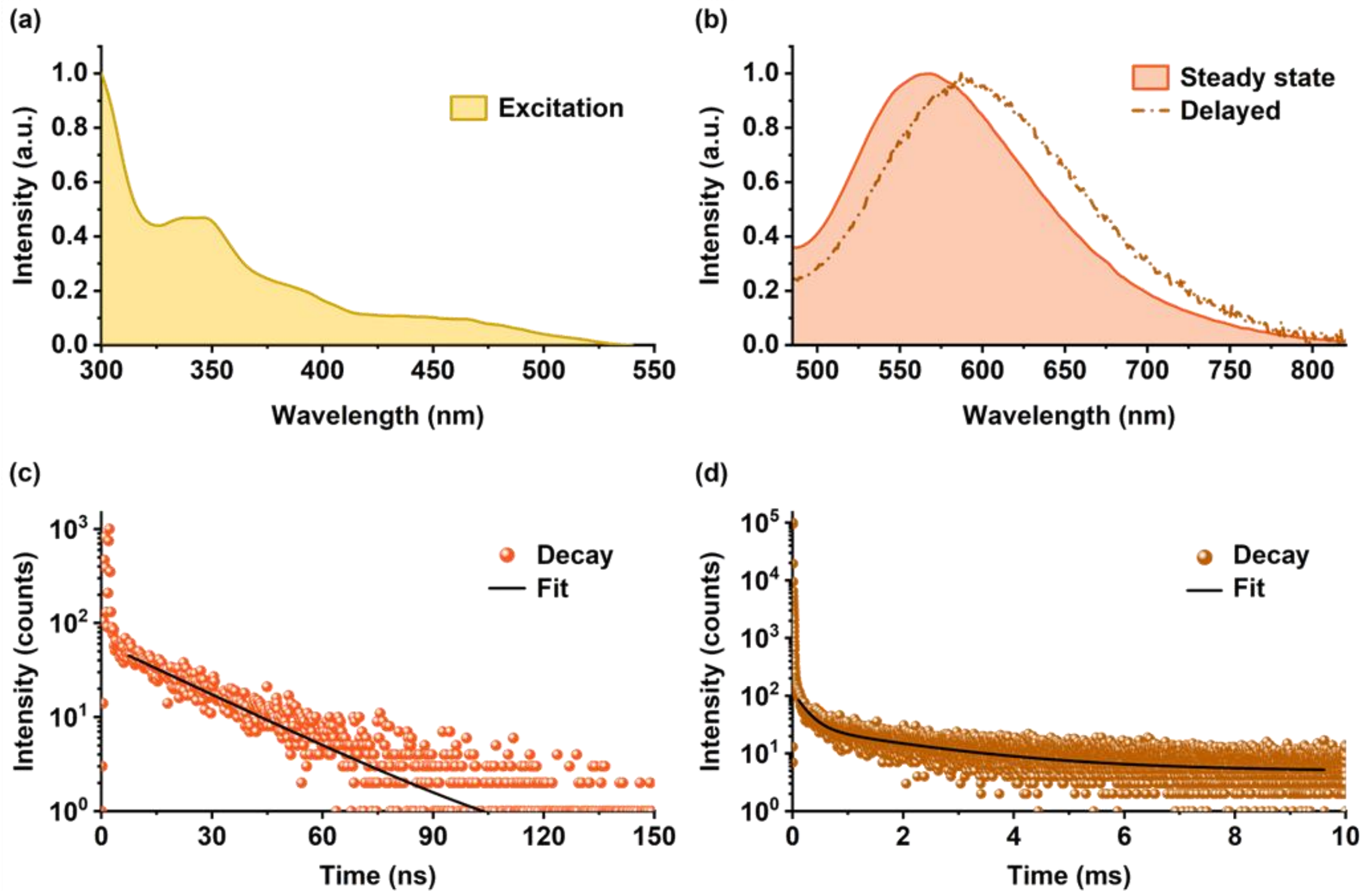

Figure S12. (a) Excitation (b) Steady-state and delayed (delay time = 50 μs) fluorescence spectra, (c) prompt and (d) delayed lifetimes of 1 wt.% **NBICz–F** doped in poly(methyl methacrylate) (PMMA) matrix (T = 293 K, $\lambda_{exc.}$ = 400 nm, for lifetime measurements, $\lambda_{collected}$ = 565 nm, all measurements were performed in ambient conditions).

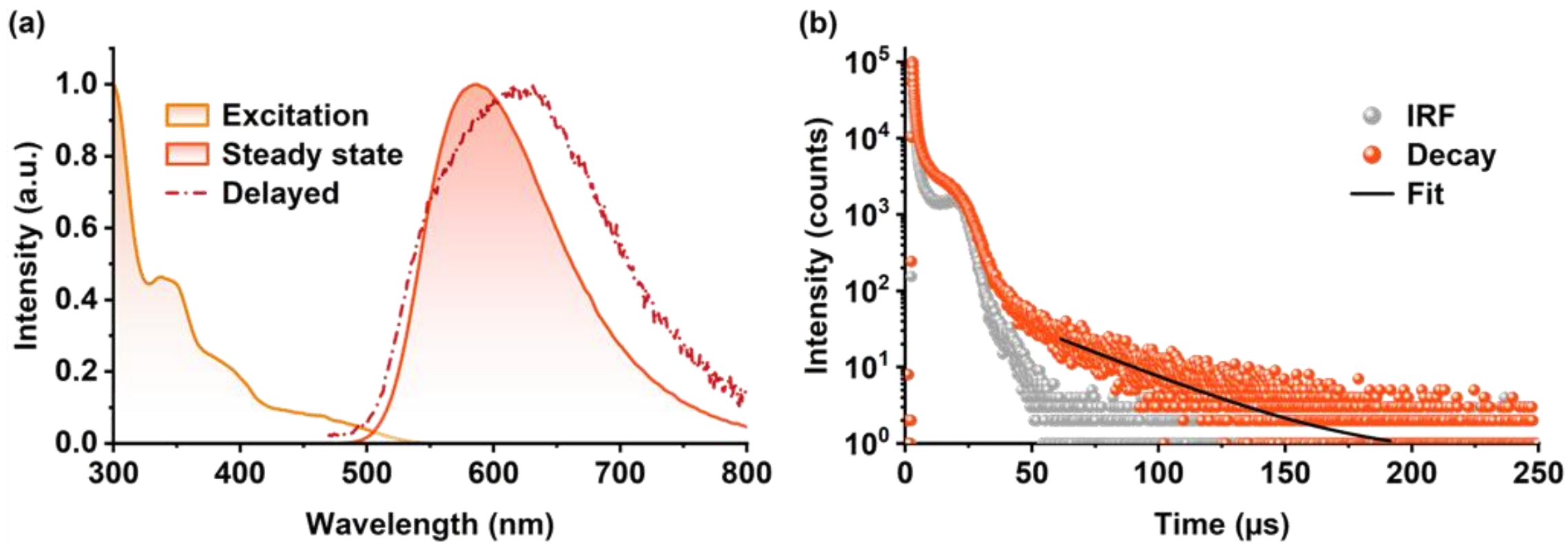

Figure S13. (a) Excitation, steady state, and delayed emission spectra and (b) time-resolved decay profile of **NBICz–F** in amorphous thin film under aerated conditions (T = 293 K, $\lambda_{exc.}$ = 400 nm, $\lambda_{collected}$ = 585 nm; for gated spectra, delay time = 50 μs, frequency = 100 Hz, gate width = 9 ms, source period = 10 ms; excitation spectra was collected at the respective emission maxima; IRF = Instrument Response Function).

Note: The red shift in delayed fluorescence (DF) spectra compared to the prompt fluorescence (PF) spectra might be attributed to excited-state conformational heterogeneity of **NBICz–F** in the solid state (thin film).

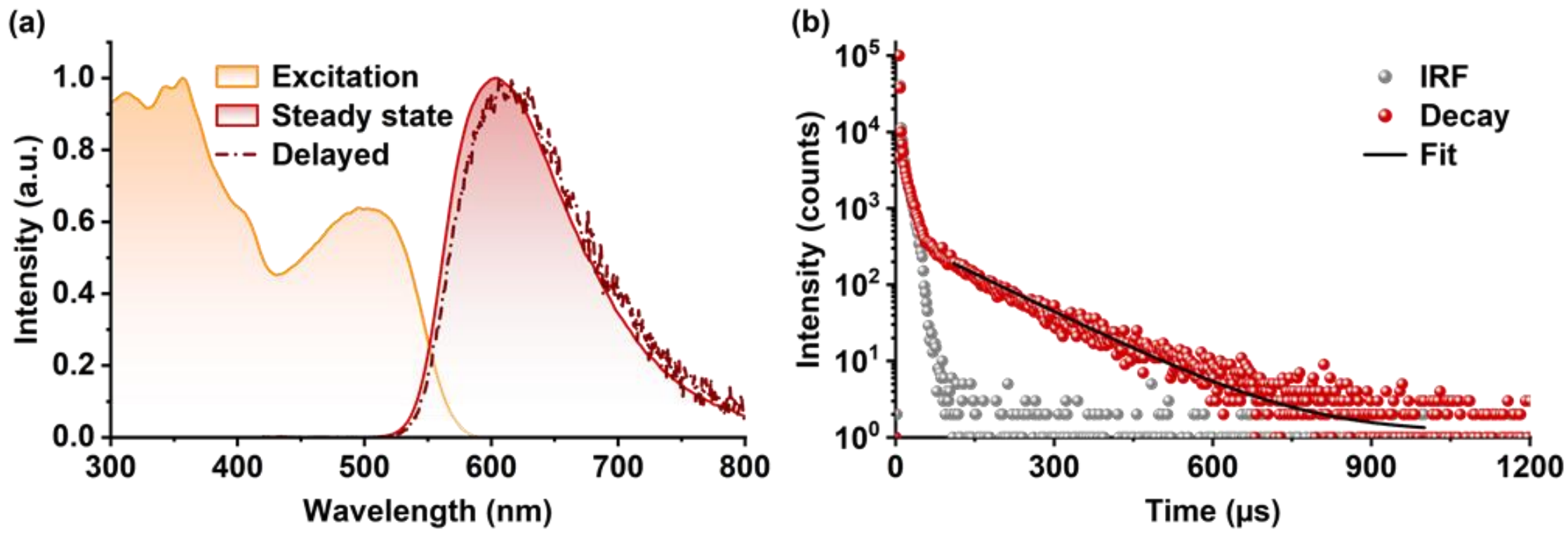

Figure S14. (a) Excitation, steady state, and delayed emission spectra and (b) time-resolved decay profile of **NBICz–Cy** in amorphous thin film under aerated conditions (T = 293 K, $\lambda_{exc.}$ = 450 nm, $\lambda_{collected}$ = 605 nm; for gated spectra, delay time = 50 μs, frequency = 100 Hz, gate width = 9 ms, source period = 10 ms; excitation spectra was collected at the respective emission maxima; IRF = Instrument Response Function).

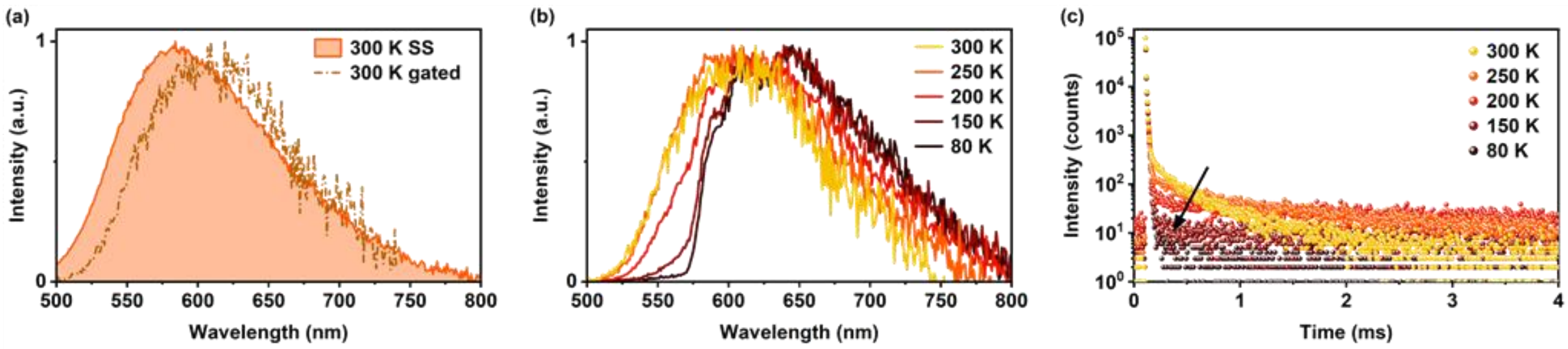

Figure S15. (a) Normalized steady state and delayed emission (delay time = 50 μs) spectra at 300 K, (b) temperature-dependent normalized delayed emission spectra (delay time = 50 μs), and (c) temperature-dependent delayed fluorescence lifetime decay profiles of **NBICz–F** in PMMA doped film ($\lambda_{exc.}$ = 400 nm, $\lambda_{collected}$ = 585 nm).

Note: In case of **NBICz–F**, there is a significant shift in the delayed fluorescence spectra (50 μs delay) compared to the prompt fluorescence spectra in the doped film, which can be attributed to the conformational heterogeneity in the excited state due to the flexible architecture of the foldamer. Temperature-dependent studies clearly demonstrate that there is a presence of phosphorescence at low temperatures. With an increase in temperature, the phosphorescence band disappears, and the delayed fluorescence prevails. From the temperature-dependent time-resolved decay analysis, it is evident that upon a decrease in temperature (from 300 K to 80 K), there is a decrease in delayed fluorescence lifetime, and it eventually disappears at lower temperatures. At 300 K (room temperature), there is no prominent signature of ambient triplet emission, rather delayed fluorescence is the predominant emission pathway.

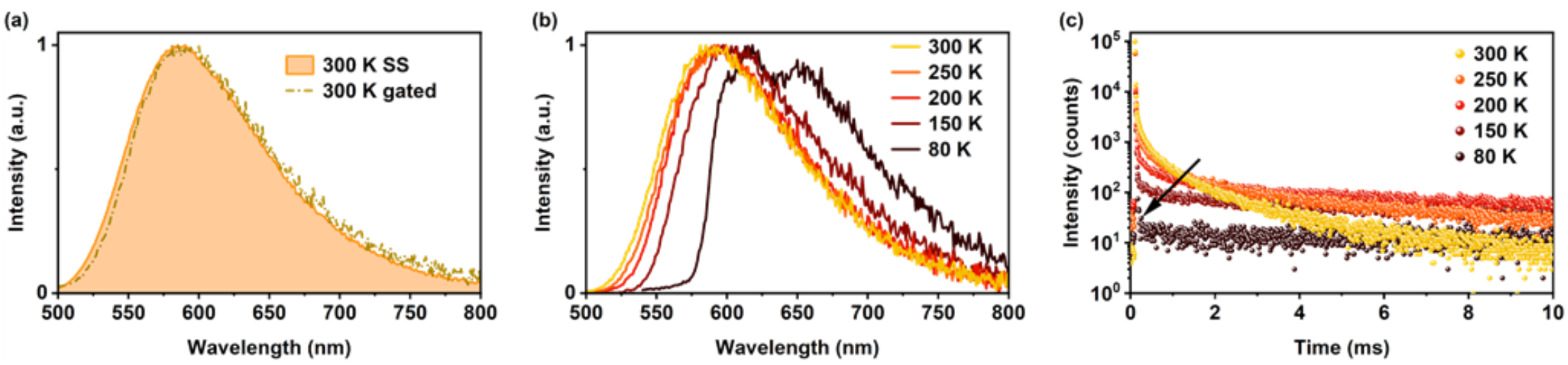

Figure S16. (a) Normalized steady state and delayed emission (delay time = 50 μs) spectra at 300 K, (b) temperature-dependent normalized delayed emission spectra (delay time = 50 μs), and (c) temperature-dependent delayed fluorescence lifetime decay profiles of **NBICz–Cy** in PMMA doped film ($\lambda_{exc.}$ = 450 nm, $\lambda_{collected}$ = 570 nm).

Note: NBICz–Cy exhibits a negligible shift between the steady-state and gated emission spectra in the doped film, which can be attributed to reduced structural reorganization in the excited state arising from the rigid hetero-cyclophane framework. Temperature-dependent emission and lifetime measurements further suggest that the observed emission originates predominantly from a delayed fluorescence process, with no significant contribution from phosphorescence under ambient conditions. At 300 K, no prominent phosphorescence emission is observed, and delayed fluorescence remains the dominant emission pathway. However, at lower temperatures, phosphorescence contributions become apparent alongside the delayed fluorescence emission.

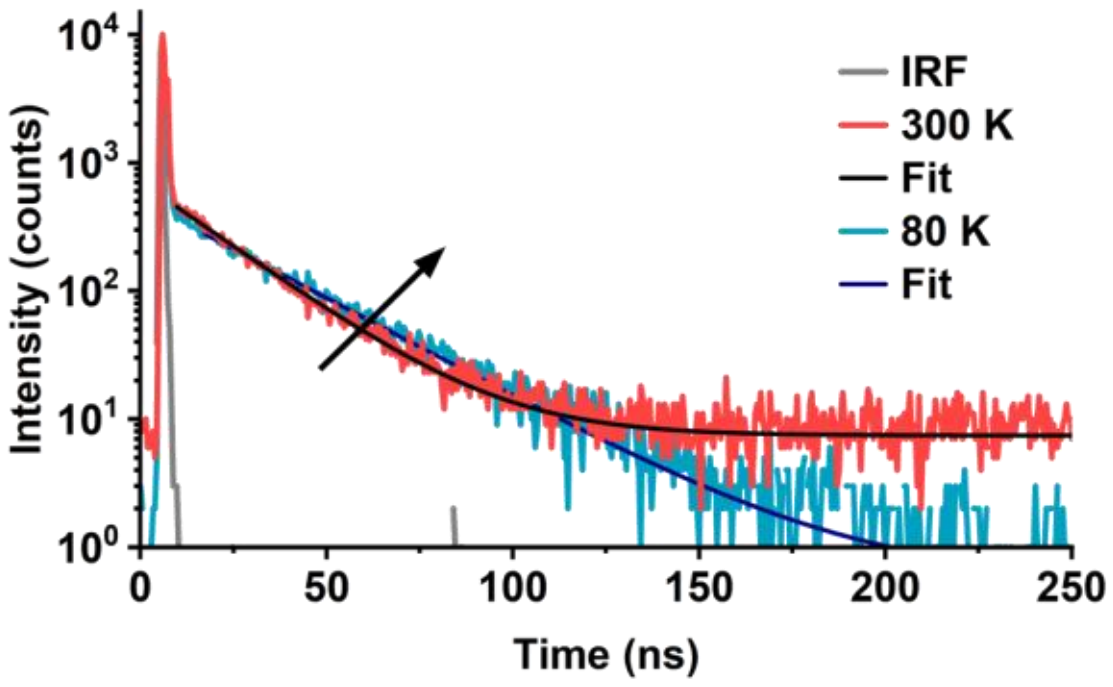

Figure S17. Temperature-dependent prompt fluorescence (PF) lifetimes of 1 wt.% **NBICz–Cy** in PMMA doped film ($\lambda_{exc.}$ = 450 nm, $\lambda_{collected}$ = 590 nm).

Note: With a decrease in temperature, there is an increase in the PF lifetime due to decreased molecular motions and, therefore, reduced non-radiative deactivation pathways.

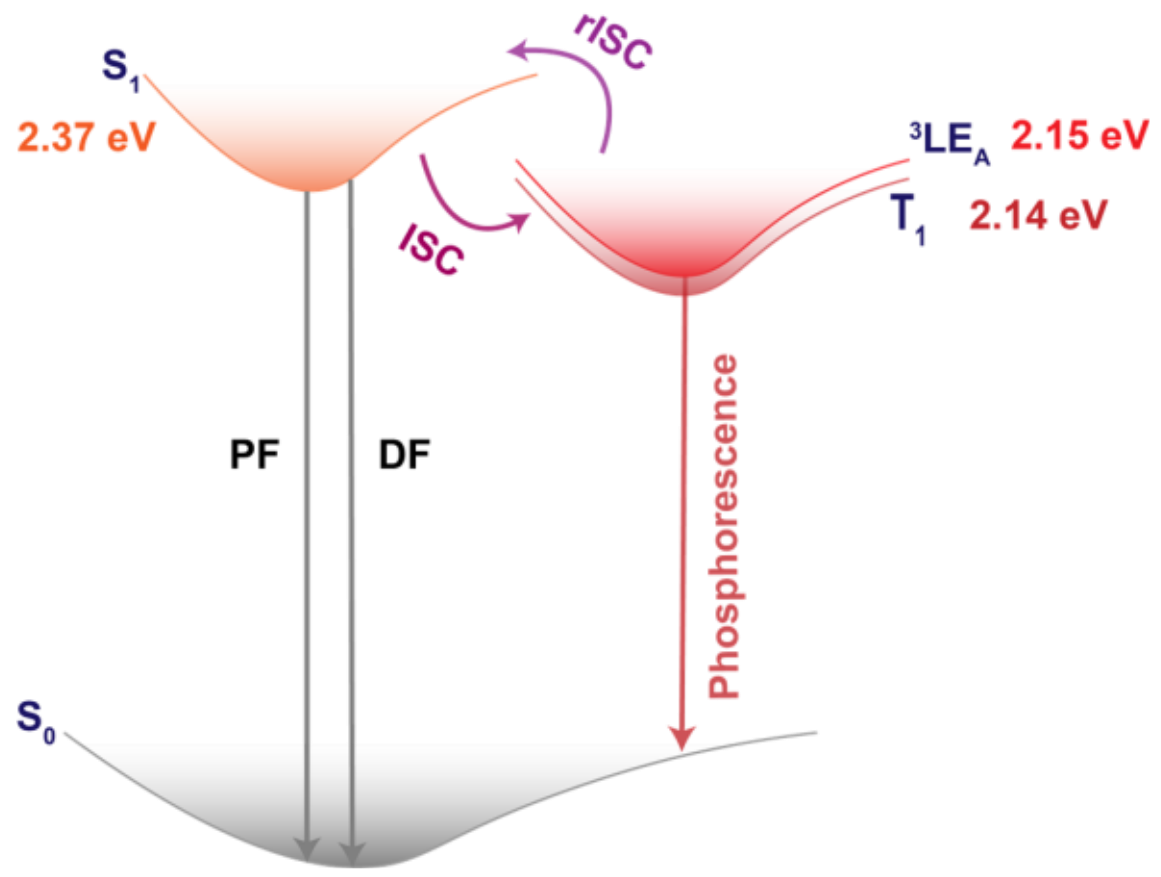

Figure S18. Simplified Jablonski diagram showing the plausible mechanism of delayed fluorescence in **NBICz–Cy** (in 1 wt.% PMMA film).

Note: The lowest energy singlet and triplet (S_1 and T_1) energies were calculated from the onsets of the delayed fluorescence (300 K, 50 µs delay) and phosphorescence spectra (80 K, 2 ms delay) of **NBICz–Cy** (1 wt.% compound doped in PMMA matrix). Whereas the ^3LE_A (energy of the lowest triplet excited state of the pristine acceptor 1,2:5,6–NBI-Ref) was calculated from the onset of the phosphorescence spectra of 1,2:5,6–NBI-Ref at 80 K. It is evident that the ^3LE_A and T_1 states are energetically close to each other.

Table S2. Summary of prompt fluorescence (^{1}CT) decay of **NBICz–F** under air and argon atmosphere at room–temperature ($\lambda_{exc.}$ = 400 nm, T = 293 K).

Solvent	Conditions	$\lambda_{em.}$ (nm)	τ_{PF} (ns)	χ^2
CCl_4	Air	570	14.6	0.94
TOL		590	14.1	0.89
CCl_4	Ar	570	22.3	0.99
TOL		590	22.8	0.99

Table S3. Summary of prompt fluorescence ([1]CT) decay of **NBICz–Cy** under air and argon atmosphere at room–temperature ($\lambda_{exc.}$ = 450 nm, T = 293 K).

**Solvent**	**Conditions**	**$\lambda_{em.}$ (nm)**	**τ_{PF} (ns)**	**χ^2**
MCH	Air	560	12	1.02
CCl_4		575	15.3	1.04
TOL		595	13.6	0.99
MCH	Ar	560	23	1.17
CCl_4		575	22.5	1.01
TOL		595	20.2	1.29

Table S4. Summary of photophysical properties of 1 wt.% **NBICz–F** and **NBICz–Cy** in PMMA matrix under air at room–temperature ($\lambda_{exc.}$ = 400 nm, $\lambda_{collected}$ = 565 nm, T = 293 K, PVA layer was coated over the PMMA matrix to prevent oxygen penetration).

**$\lambda_{em.}$ (nm)**	**τ_{PF} (ns)**	**τ_{DF} (μs)**	**Φ (%)**
565	23	808	4
585	21	962	12

Table S5. Summary of photophysical properties of **NBICz–F** and **NBICz–Cy** neat film under air at room–temperature ($\lambda_{exc.}$ = 400 nm, T = 293 K).

**$\lambda_{em.}$ (nm)**	**τ_{DF} (μs)**	**PLQY (%)**	**χ^2**
585	32.5	5	1.1
605	129.6	10	1.14

Table S6. Summary prompt fluorescence (PF) lifetimes of 1 wt.% **NBICz–Cy** in PMMA doped film at room–temperature and 80 K ($\lambda_{exc.}$ = 450 nm, $\lambda_{collected}$ = 590 nm).

**Temp**	**τ_{PF}**	**χ^2**
300 K	21 ns	1.19
80 K	28.2 ns	1.05

6. Chiroptical Properties:

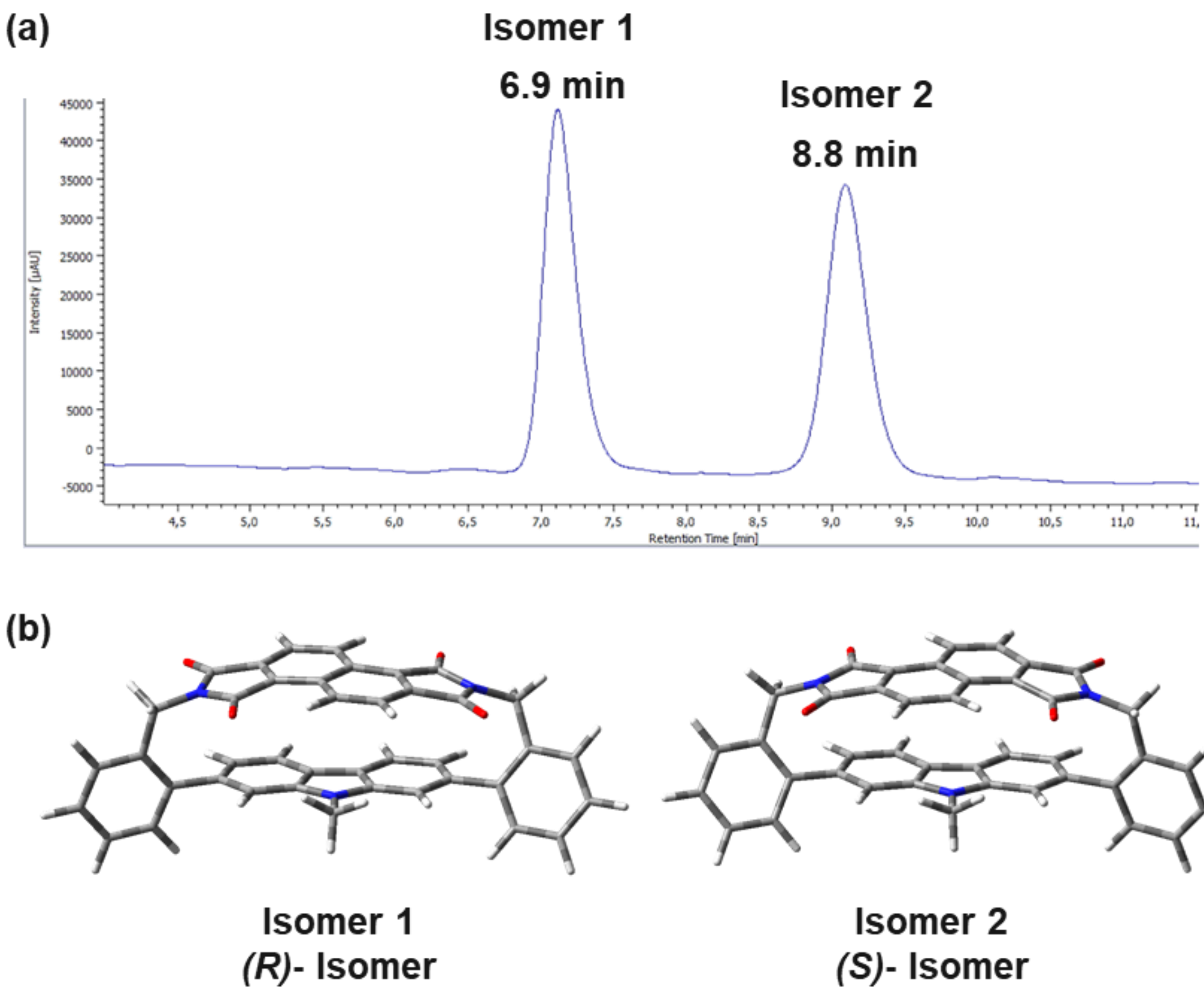

Figure S19. (a) Analytical HPLC chromatogram for the separation of **NBICz–Cy** enantiomers. (b) Calculated structures of the *(R)*- and *(S)*-**NBICz–Cy** (CAM-B3LYP-D3/def2-SVP optimized S_0 geometries and CD spectra from TD (S_1) calculations, including 15 singlet excited states).

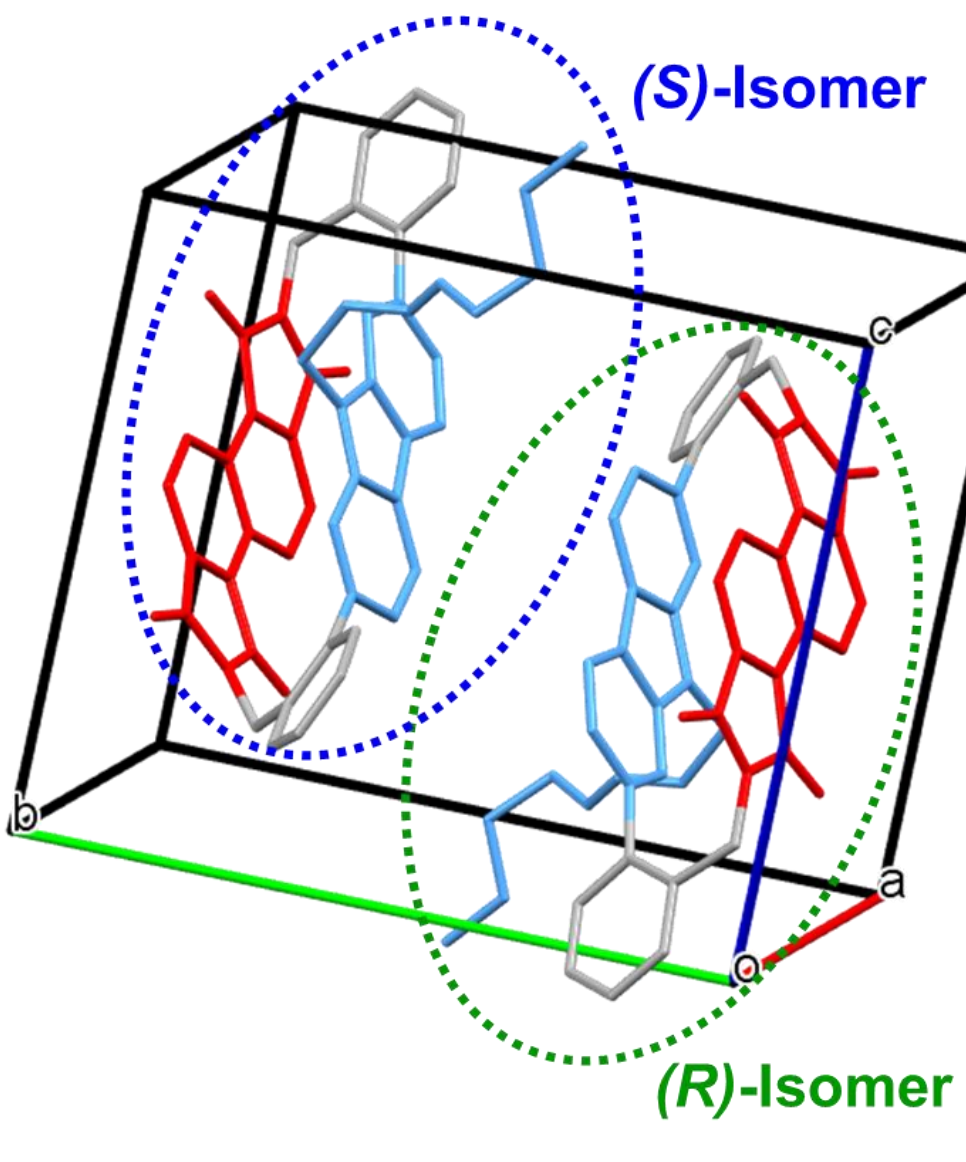

Figure S20. Assignment of the respective *(R)*- and *(S)*- isomers of **NBICz–Cy** in the Unit cell according to single-crystal X-ray analysis.

Note: From the computed structures, the respective isomers can be designated. Further in the crystal, the corresponding structures can be categorized as *(R)*- and *(S)*- isomers. As observed, the unit cell of the **NBICz–Cy** crystal comprises both *(R)*- and *(S)*- isomers, making the whole crystal racemic.

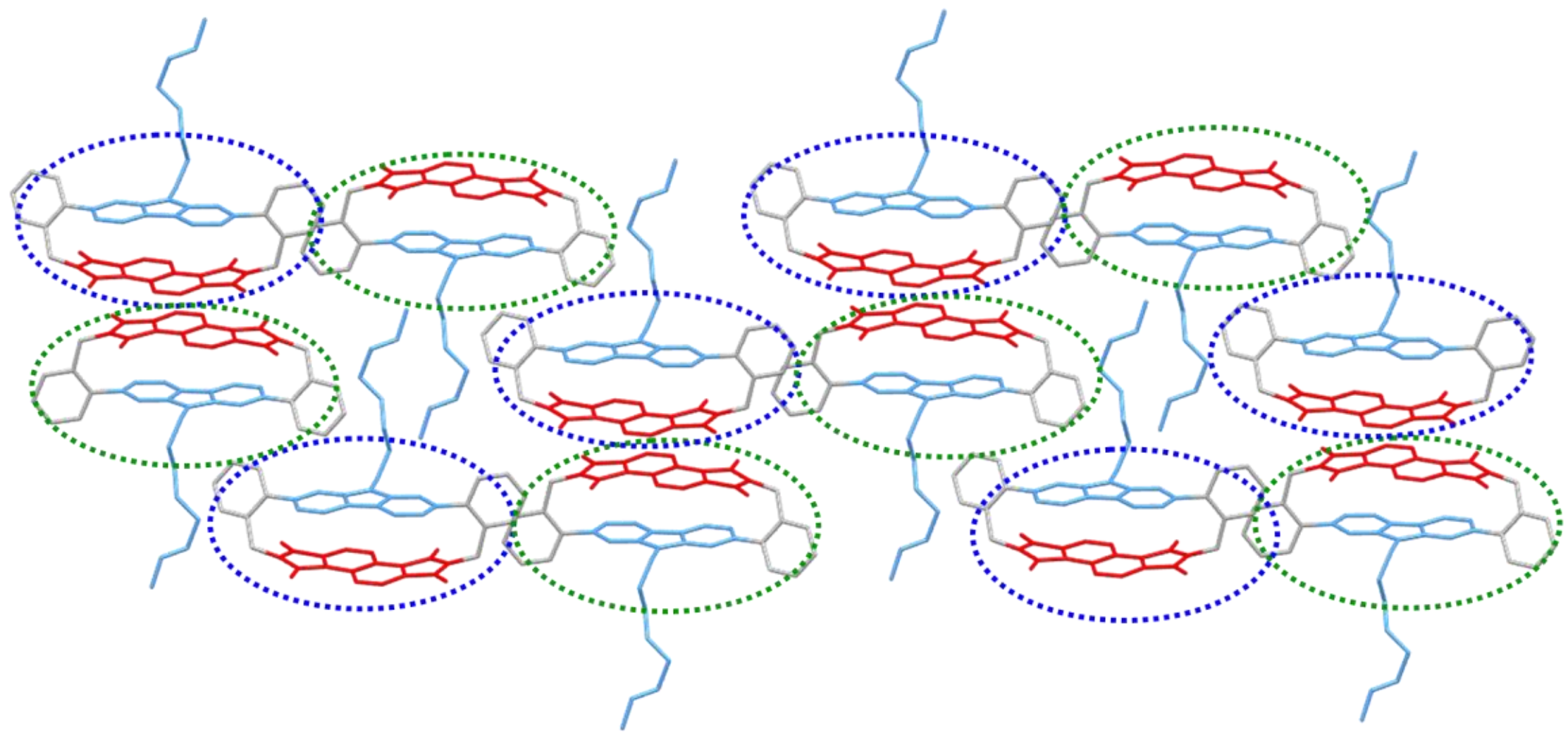

Figure S21. Presence of *(R)*- and *(S)*- isomers in the extended packing of **NBICz–Cy** in the crystal lattice according to single-crystal X-ray analysis (The green and blue circles correspond to *(R)*- and *(S)*- isomers, respectively).

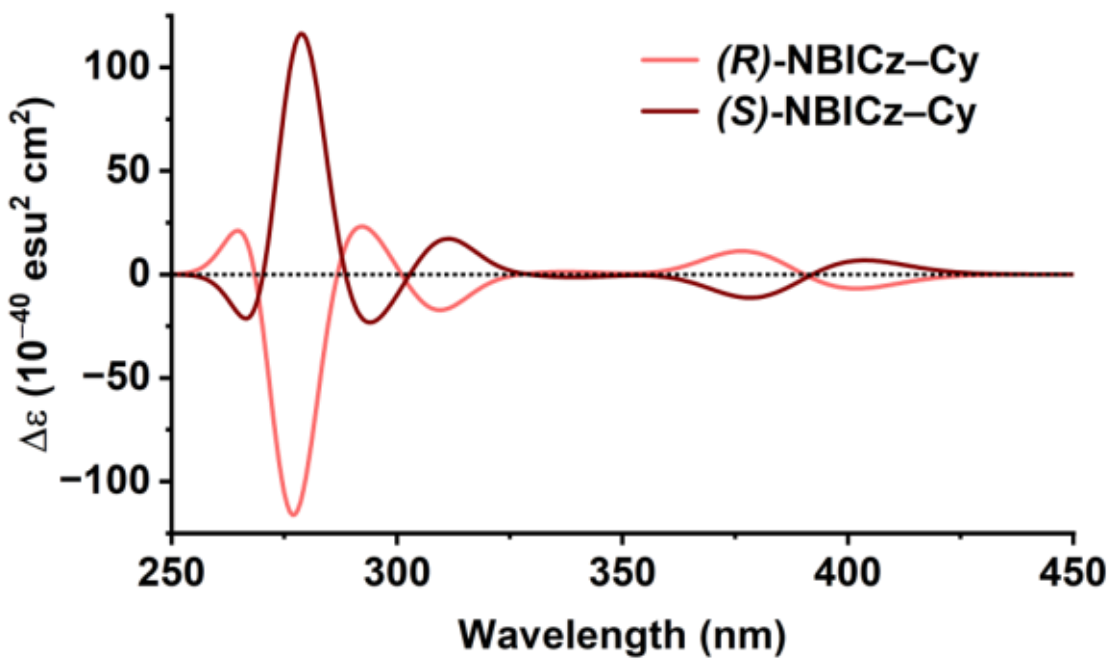

Figure S22. Theoretically calculated electronic circular dichroism (ECD) spectra of **NBICz–Cy** enantiomers (CAM-B3LYP-D3/def2-SVP optimized S_0 geometries and CD spectra from TD (S_1) calculations, including 15 singlet excited states. CD spectrum simulated with a HWHM peak of 0.1 eV using GaussView).

Note: The computed ECD spectra show clear structural resemblance with the experimental ECD spectra.

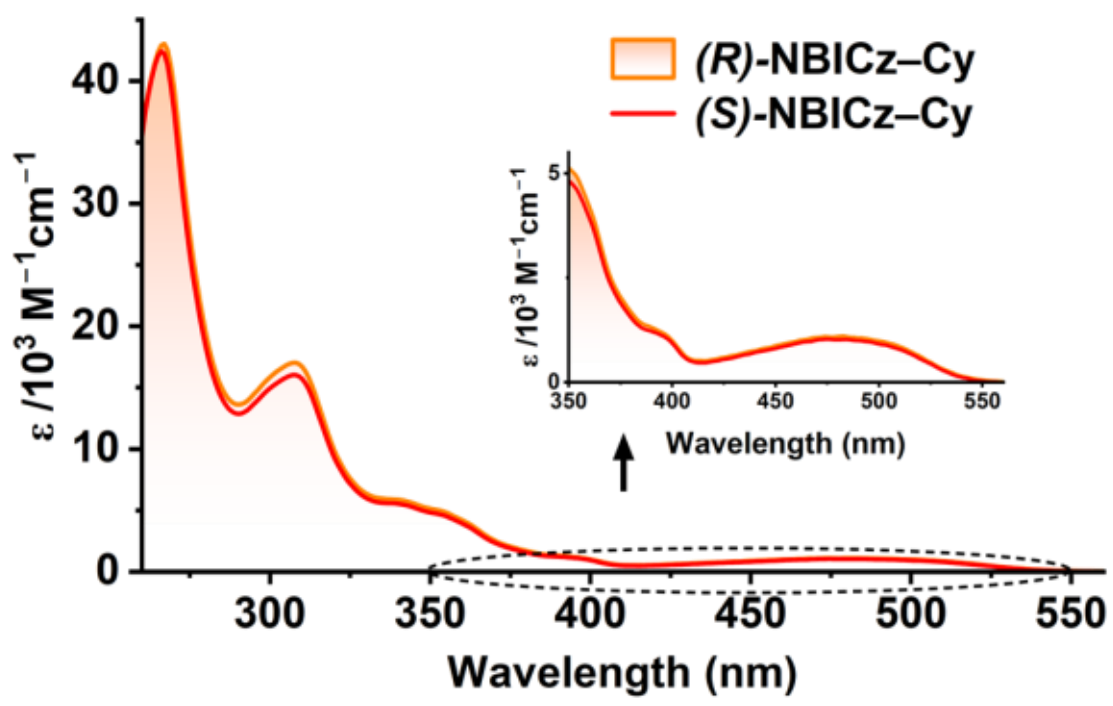

Figure S23. UV-visible absorption spectra of **NBICz–Cy** isomers in CCl_4 solution at 293 K (c ~ 1×10^{-4} M for both cases, $\lambda_{exc.}$ = 370 nm).

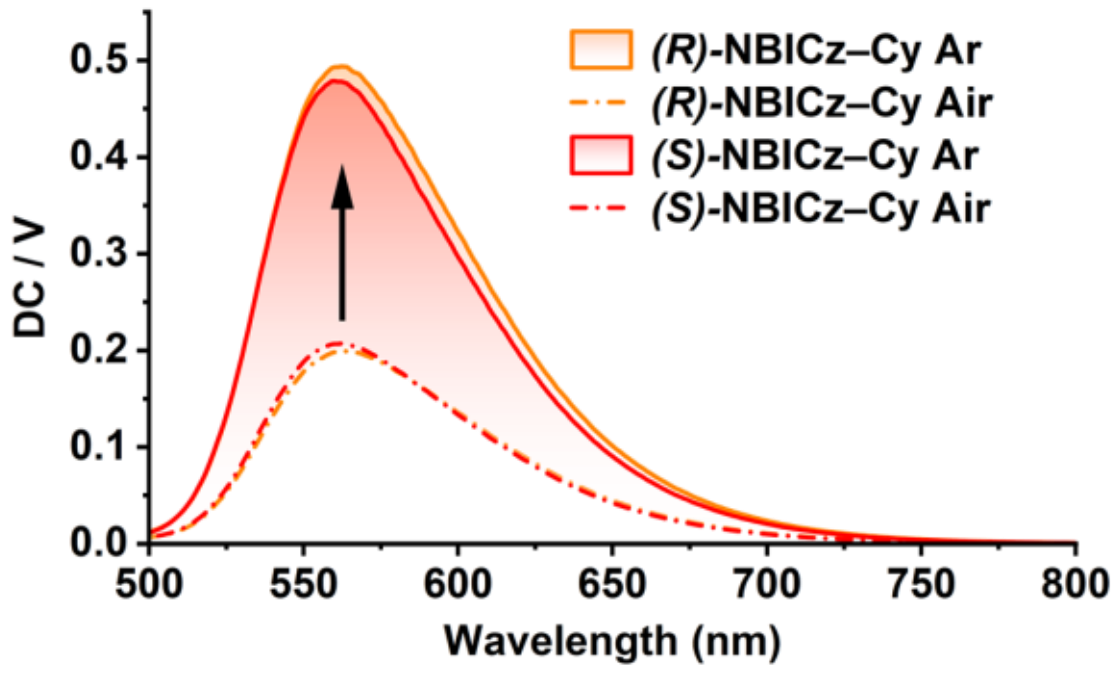

Figure S24. Direct current (DC) spectra of **NBICz–Cy** isomers in CCl_4 solution at 293 K under aerated and deaerated conditions (Ar was bubbled into the solution before the measurement, c ~ 1×10^{-4} M for both cases, $\lambda_{exc.}$ = 370 nm).

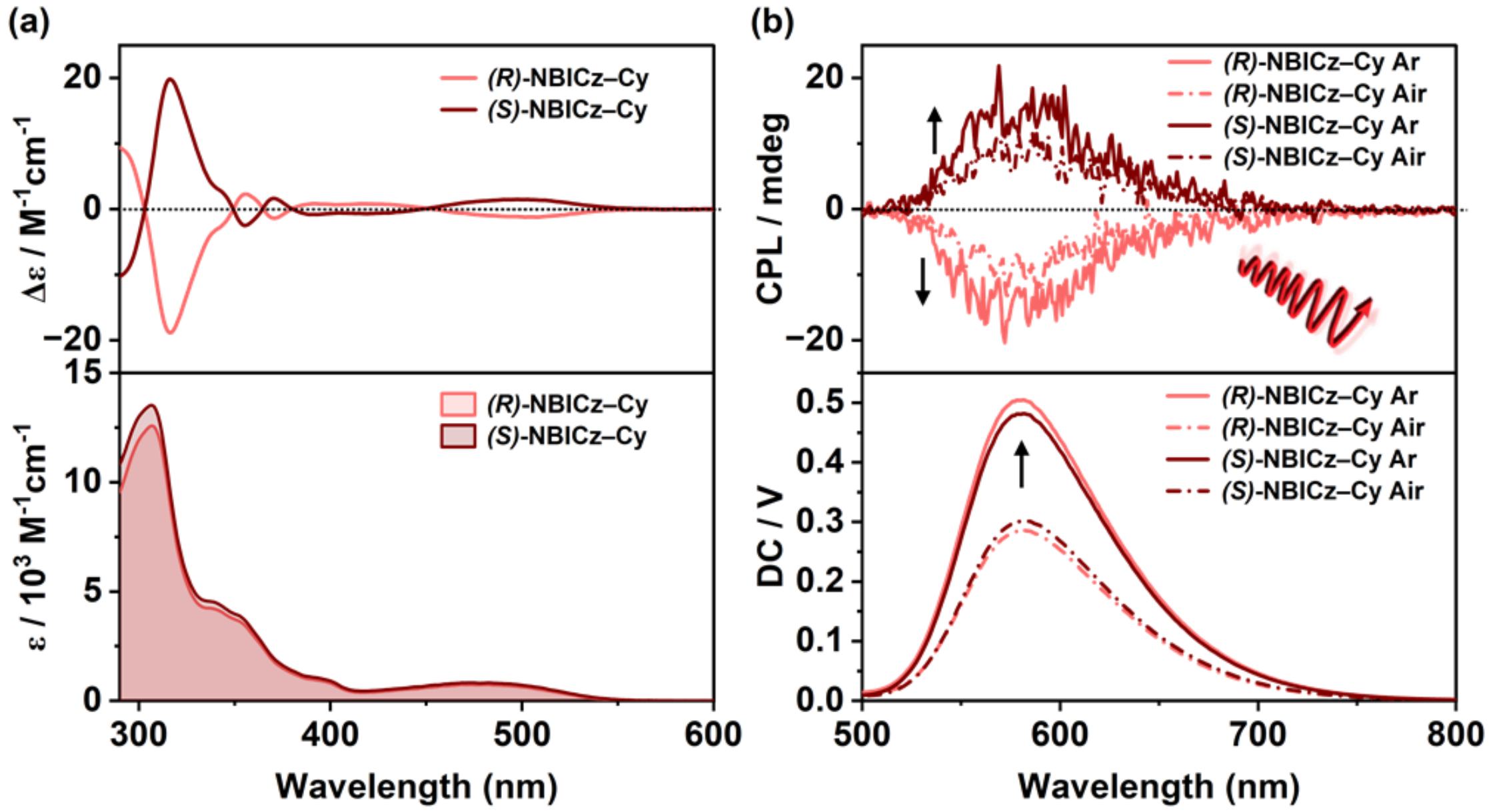

Figure S25. (a) Mirror-image ECD spectra, UV-visible absorption spectra, and (b) Circularly polarized luminescence (CPL) and direct current (DC) spectra of **NBICz–Cy** isomers in toluene solution at 293 K under aerated and deaerated conditions (Ar was bubbled into the solution before the measurement, c ~ 1×10^{-4} M for both cases, T = 293 K, $\lambda_{exc.}$ = 370 nm).

Note: Upon Ar purging, there is a significant increase in the CPL emission intensity as well as direct current (DC) of **NBICz–Cy** isomers in toluene solution, indicating the contribution of triplet excitons to the emission process.

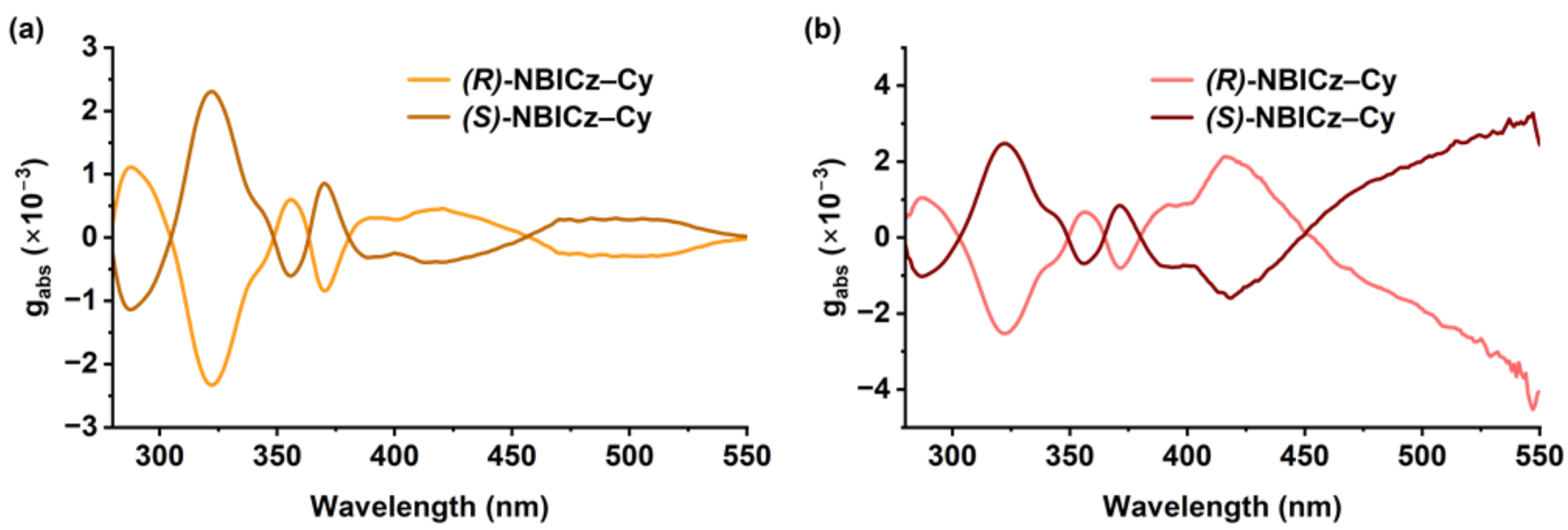

Figure S26. Plots of Absorption dissymmetry factor (g_{abs}) in solutions of **NBICz–Cy** enantiomers in (a) CCl_4 and (b) toluene (c ~ 1×10^{-4} M for both cases, T = 293 K).

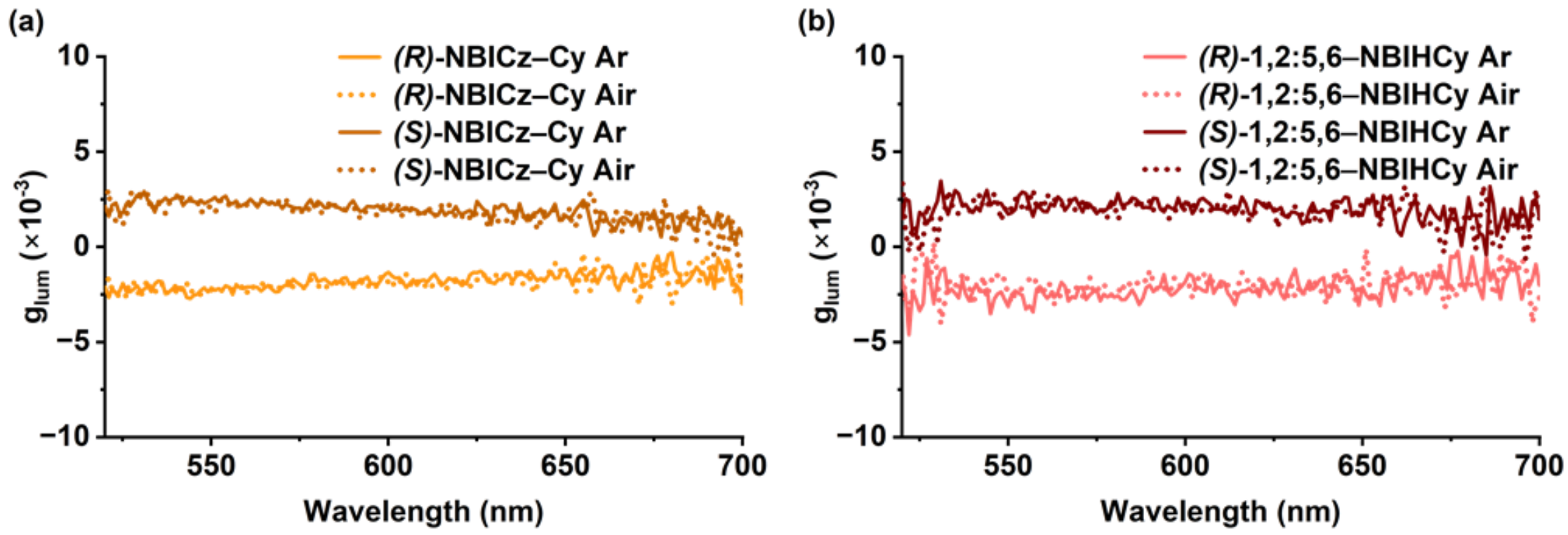

Figure S27. Plots of luminescence dissymmetry factor (g_{lum}) of **NBICz–Cy** enantiomers in aerated and deaerated solutions of (a) CCl_4 and (b) toluene (c ~ 1×10^{-4} M for both cases, T = 293 K).

Note: Upon argon purging, there is no prominent change in the luminescence dissymmetry factor (g_{lum}).

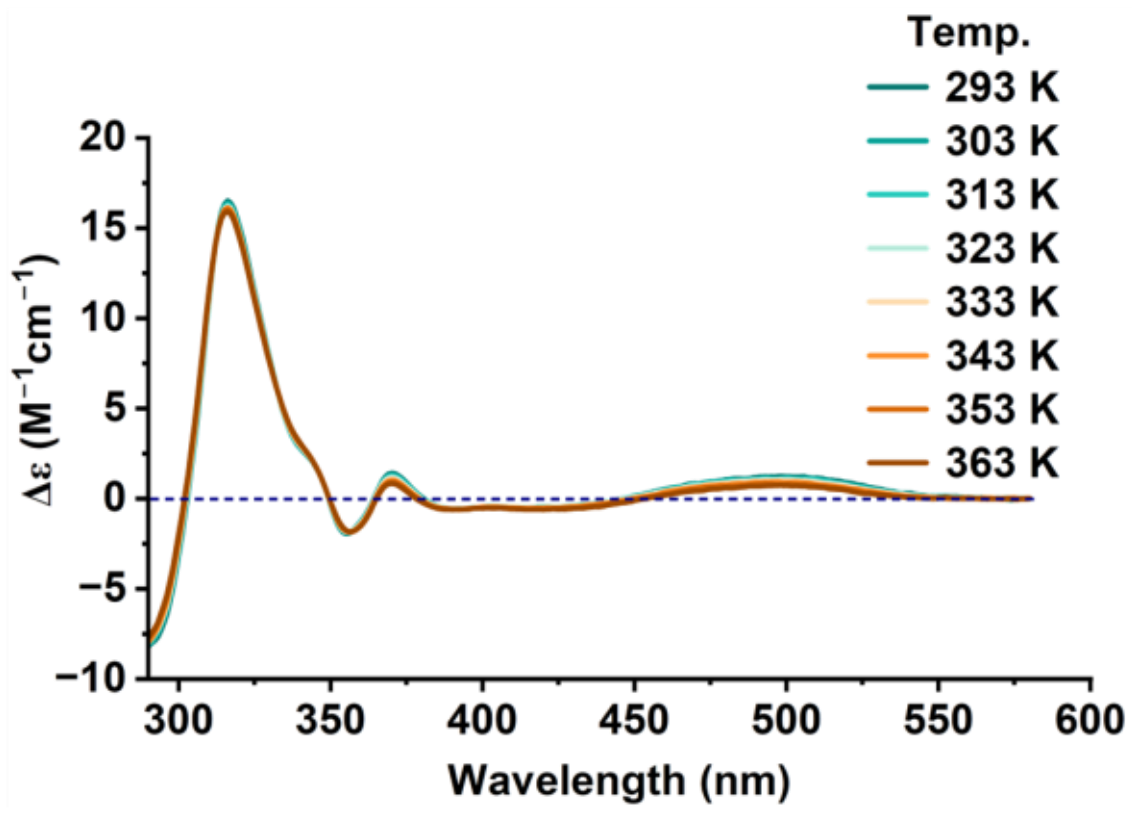

Figure S28. Temperature-dependent ECD spectra of *(S)*-**NBICz–Cy** in toluene solution (c ~ 1×10^{-4} M).

Note: With an increase in temperature up to 363 K, there is a negligible change in ECD spectra, indicating the thermal stability of the enantiomers, thereby hinting towards a high racemization barrier of the enantiomers. All the data are density corrected.[S7]

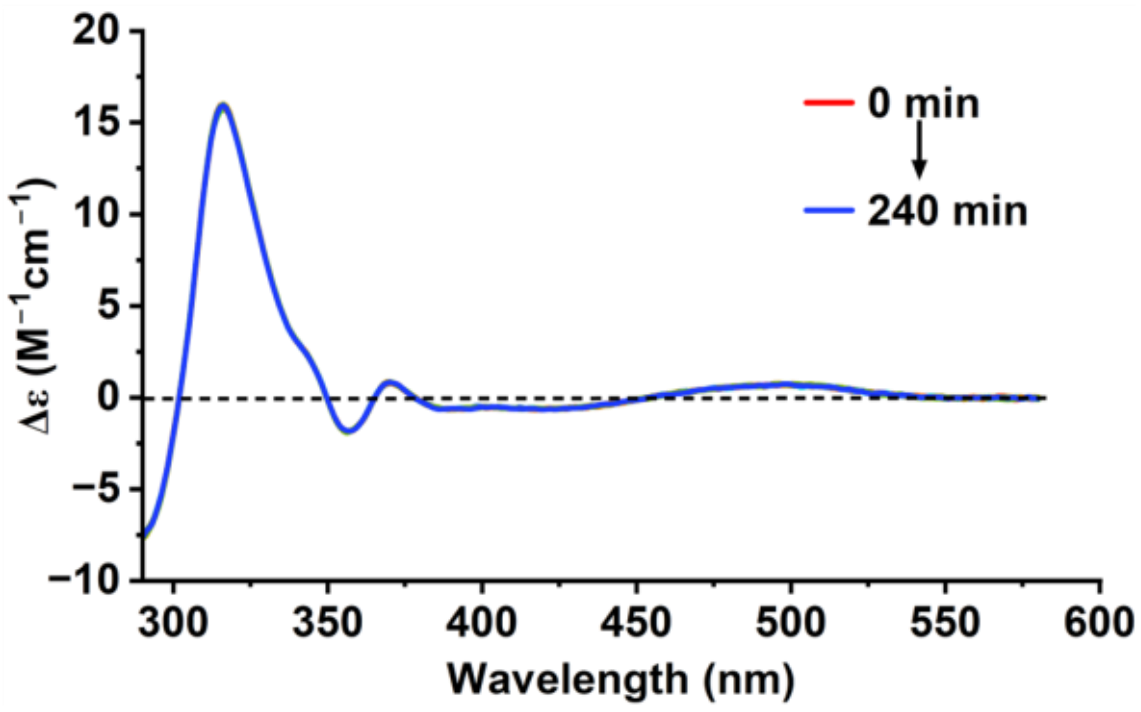

Figure S29. Time-dependent ECD spectra of *(S)*-**NBICz–Cy** in toluene solution at 363 K (c ~ 1×10^{-4} M for both cases).

Note: There is no change in the ECD spectra with varying time, suggesting a high racemization barrier of the **NBICz–Cy** enantiomers.

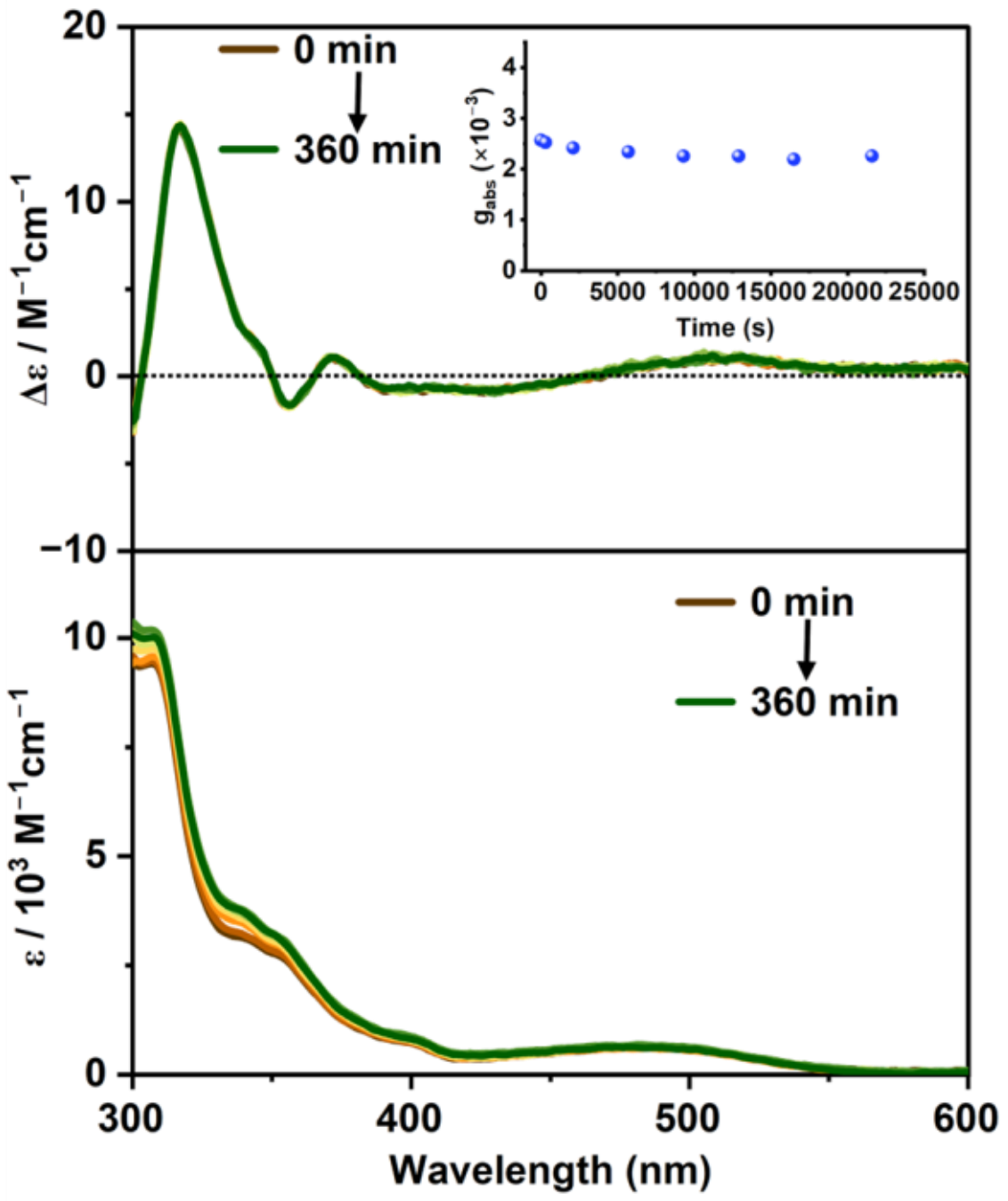

Figure S30. Time-dependent ECD spectra (top) and UV-visible absorption spectra (bottom) of (S)-**NBICz–Cy** in diphenyl ether (monitored at 498 K). The inset of the top figure shows the plot of absorption dissymmetry factor ($|g_{abs}|$) with varying time, monitored at 498 K, showing negligible change.

Note: To determine the activation parameters of **NBICz–Cy**, we performed time-dependent CD spectroscopy at 498 K. [S8] Because of the high activation barrier (as evident from Figure S28, S29), a temperature of 498 K was chosen, and therefore, high-boiling diphenyl ether was used as solvent. All samples were prepared in a temperature-resistant vial and heated to the respective temperature with an oil bath that had already been pre-heated to the desired temperature. For each measurement, the solution was rapidly cooled with cold water, and then a CD spectrum was recorded. Afterwards, the vial with solution was again placed in the oil bath at the respective temperature. An uncertainty for the temperature of σ_T = ±5 K was assumed. The timer for racemization was started each time before heating and stopped before cooling the sample. It was assumed that no racemization takes place during the CD measurement at 293 K.

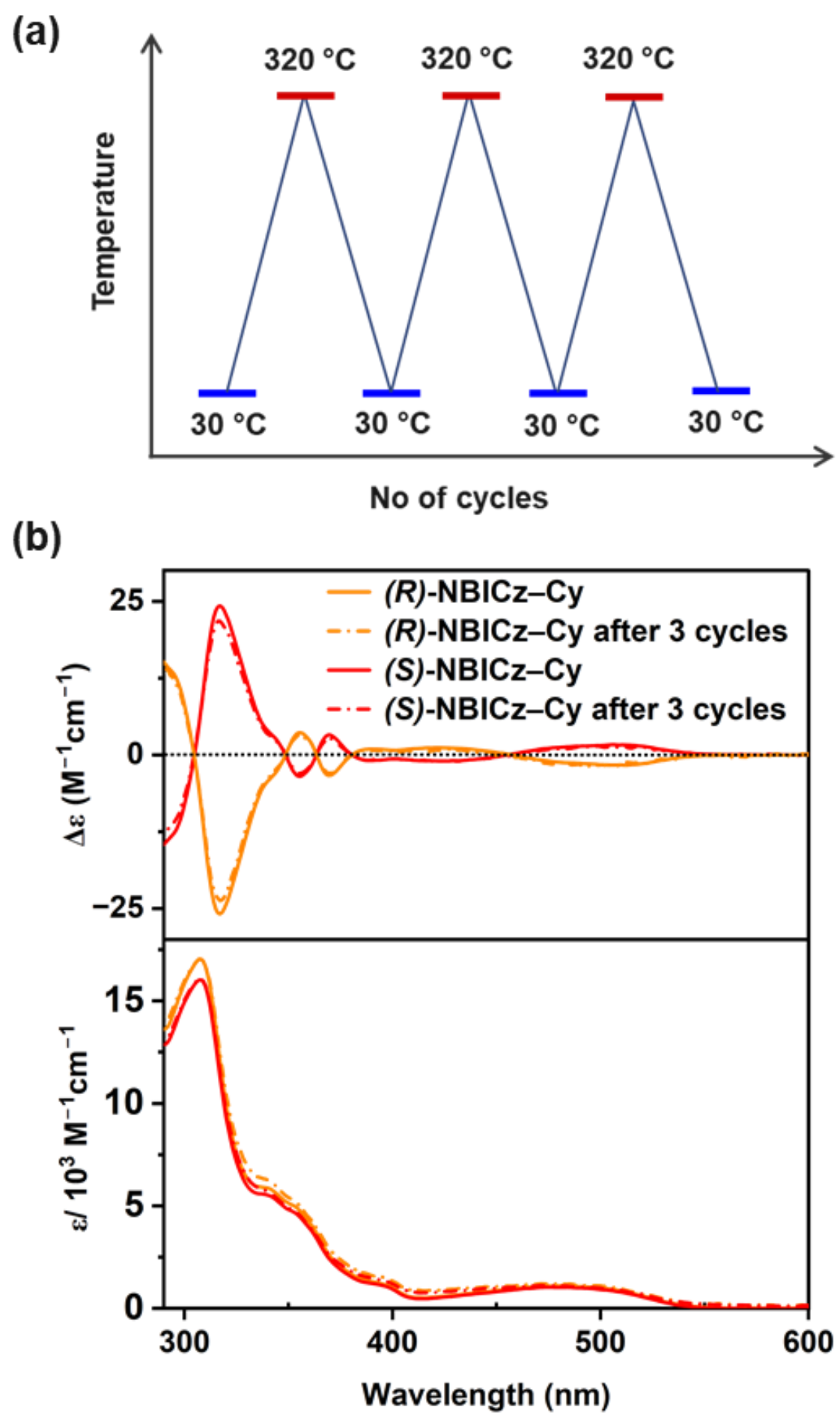

Figure S31. (a) Graphical representation of consecutive heating-cooling cycles performed. (b) Comparison in the ECD (top) and UV-visible absorption (bottom) spectra of the pristine sample (solid line) with the sample obtained after three consecutive heating-cooling cycles (dashed line).

Note: As an alternative method to understand the racemization process, we further conducted three consecutive heating and cooling cycles (30°C to 320°C and vice versa) with the individual enantiomerically pure compounds. Afterwards, we checked the enantiomeric purity of the individual enantiomers, which clearly shows that even after repetitive heating and cooling, the enantiomeric purity is retained. There is no sign of degradation or racemization.

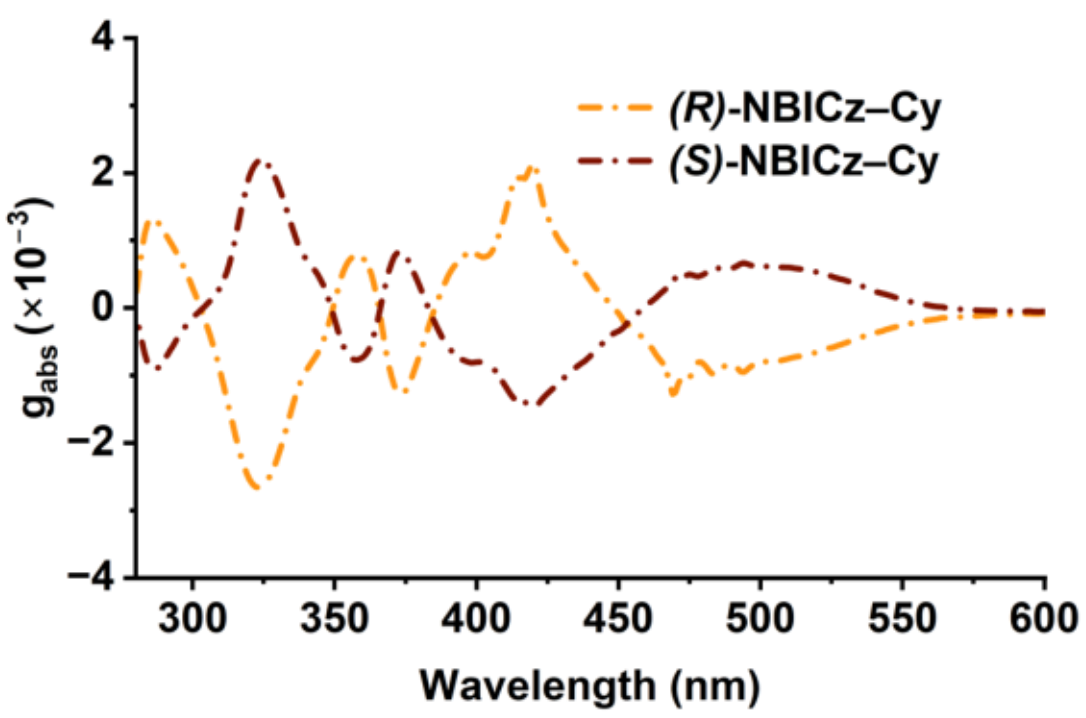

Figure S32. Plots of absorption dissymmetry factor (g_{abs}) of 1 wt.% **NBICz–Cy** isomers doped in PMMA matrix at 293 K.

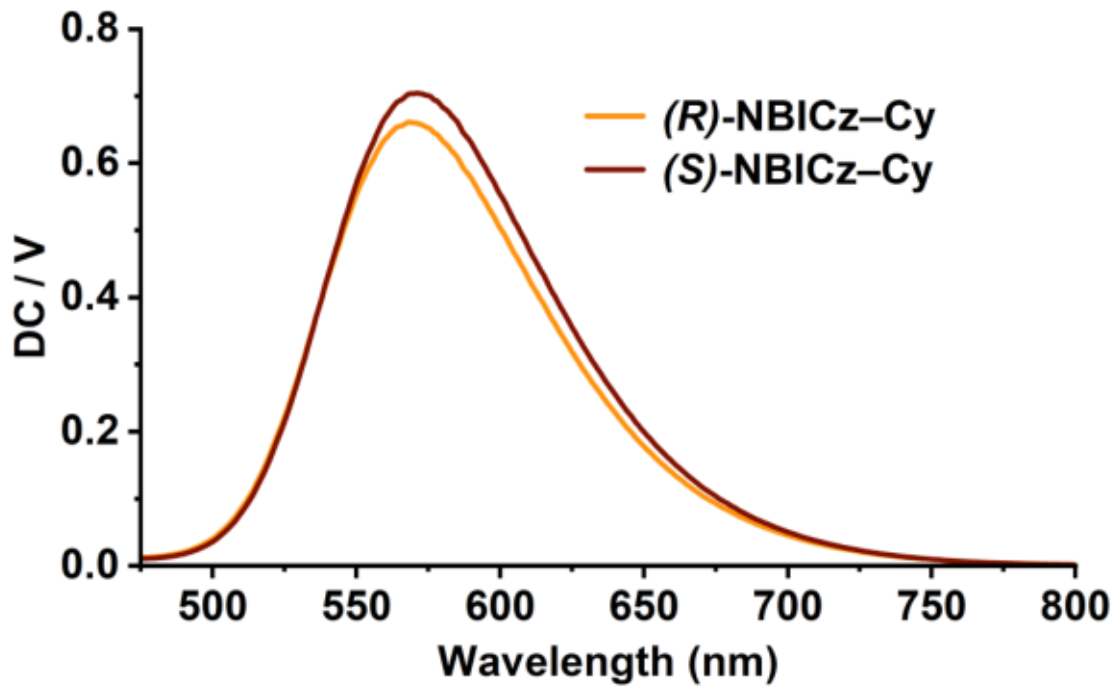

Figure S33. Direct current (DC) spectra of 1 wt. % **NBICz–Cy** isomers doped in PMMA matrix at 293 K ($\lambda_{exc.}$ = 370 nm).

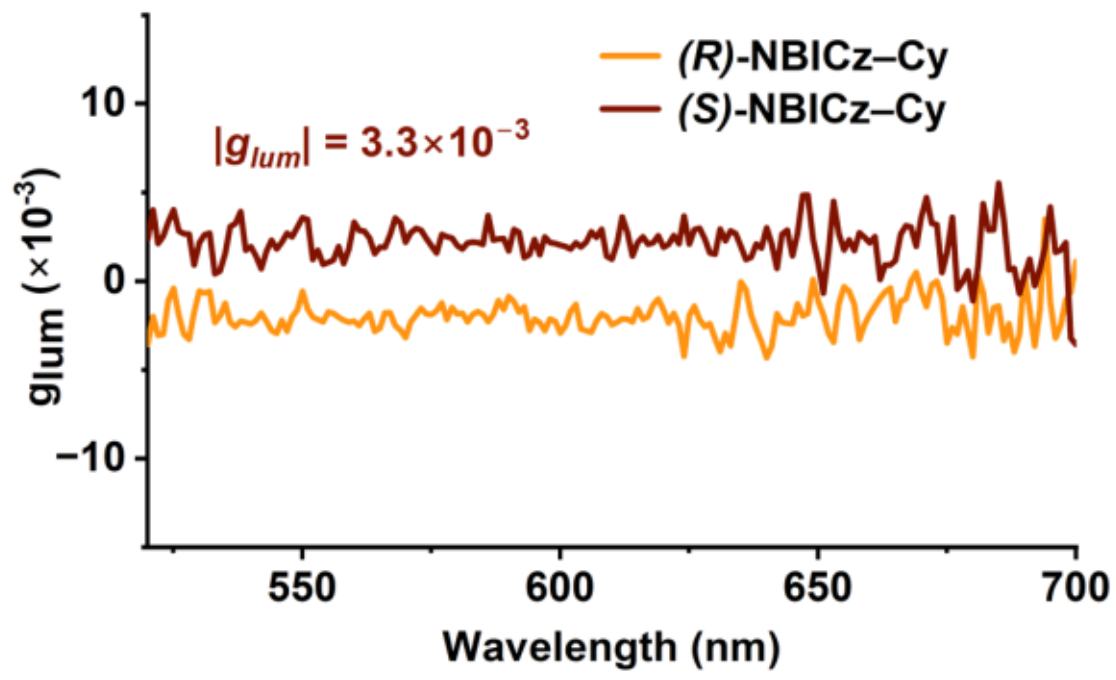

Figure S34. Plot of luminescence dissymmetry factor (g_{lum}) of 1 wt.% **NBICz–Cy** isomers doped in PMMA matrix at 293 K.

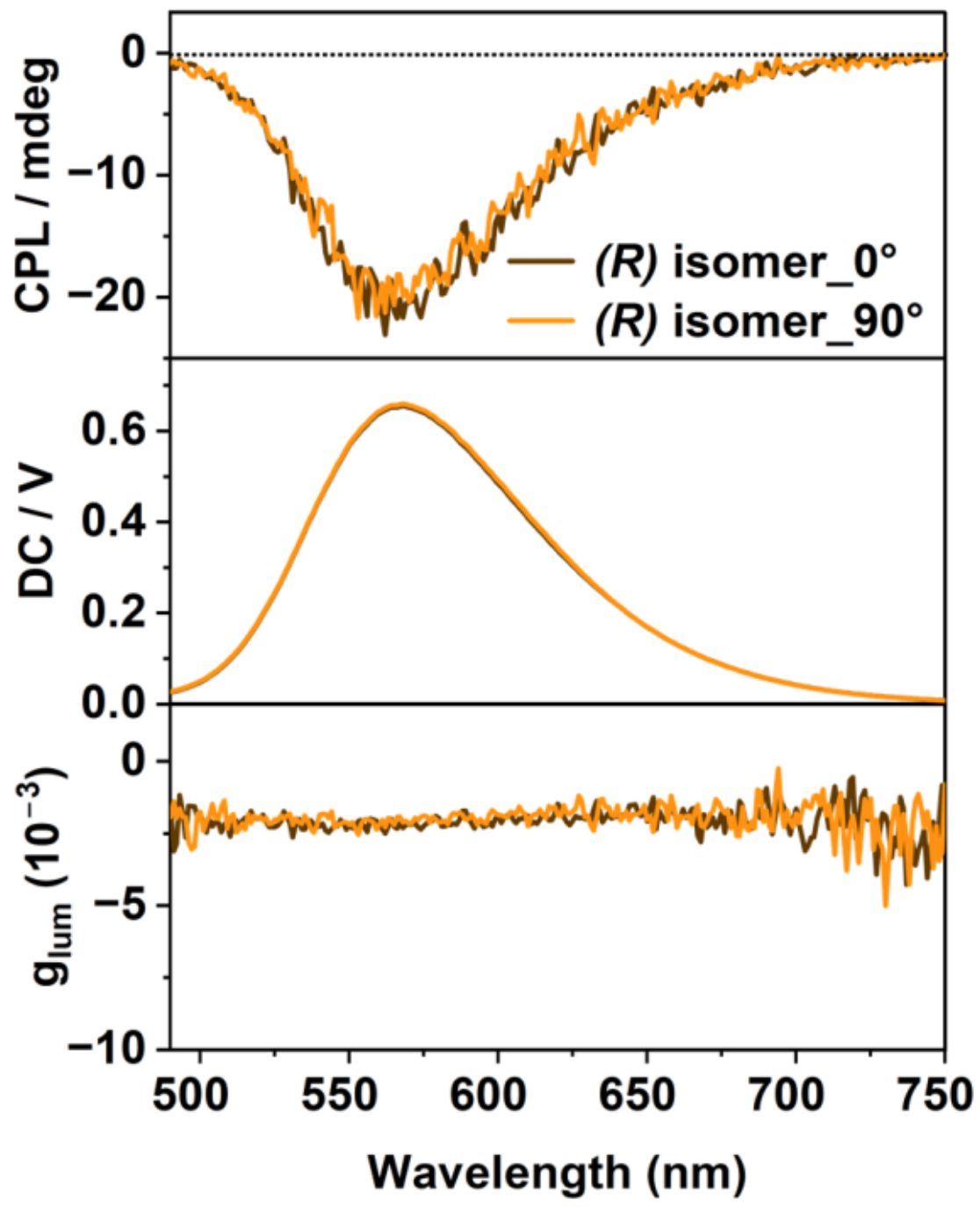

Figure S35. CPL spectra, Direct current (DC), and plot of dissymmetry factor versus wavelength of 1 wt.% *(R)*-**NBICz–Cy** isomer doped in PMMA matrix after 0° and 90° rotation of the film to the direction of light irradiation.

Note: The rotation-dependent experiments (φ = 0°, 90°, with respect to the direction of excitation light) do not show any prominent changes, thereby excluding the presence of macroscopic alignment of the hetero-cyclophane.

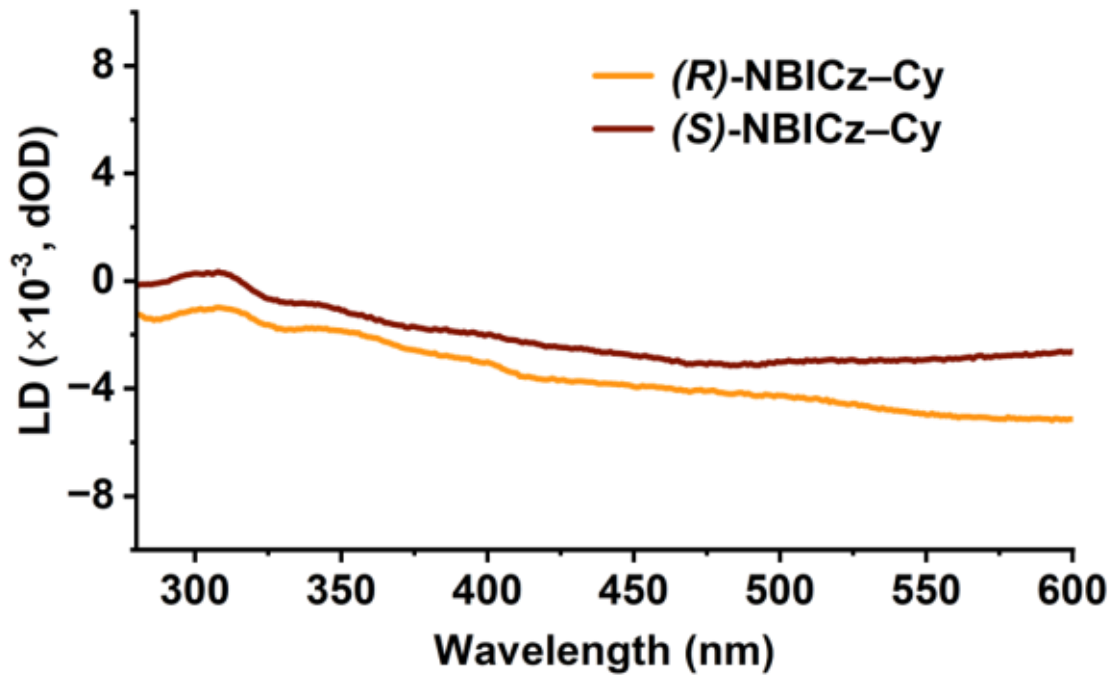

Figure S36. Linear dichroism (LD) spectra of 1 wt.% **NBICz–Cy** doped in PMMA film.

Note: Linear dichroism (LD) experiment ruled out the contribution of any macroscopic alignment of the chromophore in the thin films, which showed that the system has negligible LD contribution to the chiroptical properties.

Table S7. Summary of computed electric (μ) and magnetic (m) transition dipole moments (m), the angle between them, and the rotatory strengths (R).

	μ [a.u.]	m [a.u.]	R [cgs]	θ [°]
S_1 *(R)*	0.250	0.087	+12.6	70
S_1 *(S)*	0.250	0.087	−12.6	110
S_2 *(R)*	0.063	0.116	−18.2	148
S_2 *(S)*	0.063	0.116	+18.2	32

Note: The table above summarizes the computed electric (μ) and magnetic (m) transition dipole moments (m), the angle between them, and the rotatory strengths (R). For the first CT state S_1, μ is larger than m. However, in the second CT state S_2, the trend is inverted: m is larger than μ. The magnitude of R is thus larger than for S_1, and the μ/m angle is also closer to 0° or 180°. The structure of **NBICz–Cy** in solution might be different from the optimized or crystal structure, and S_2 calculated here might, in fact, be the lowest emissive CT state (Figure S3).

7. NMR Spectroscopy:

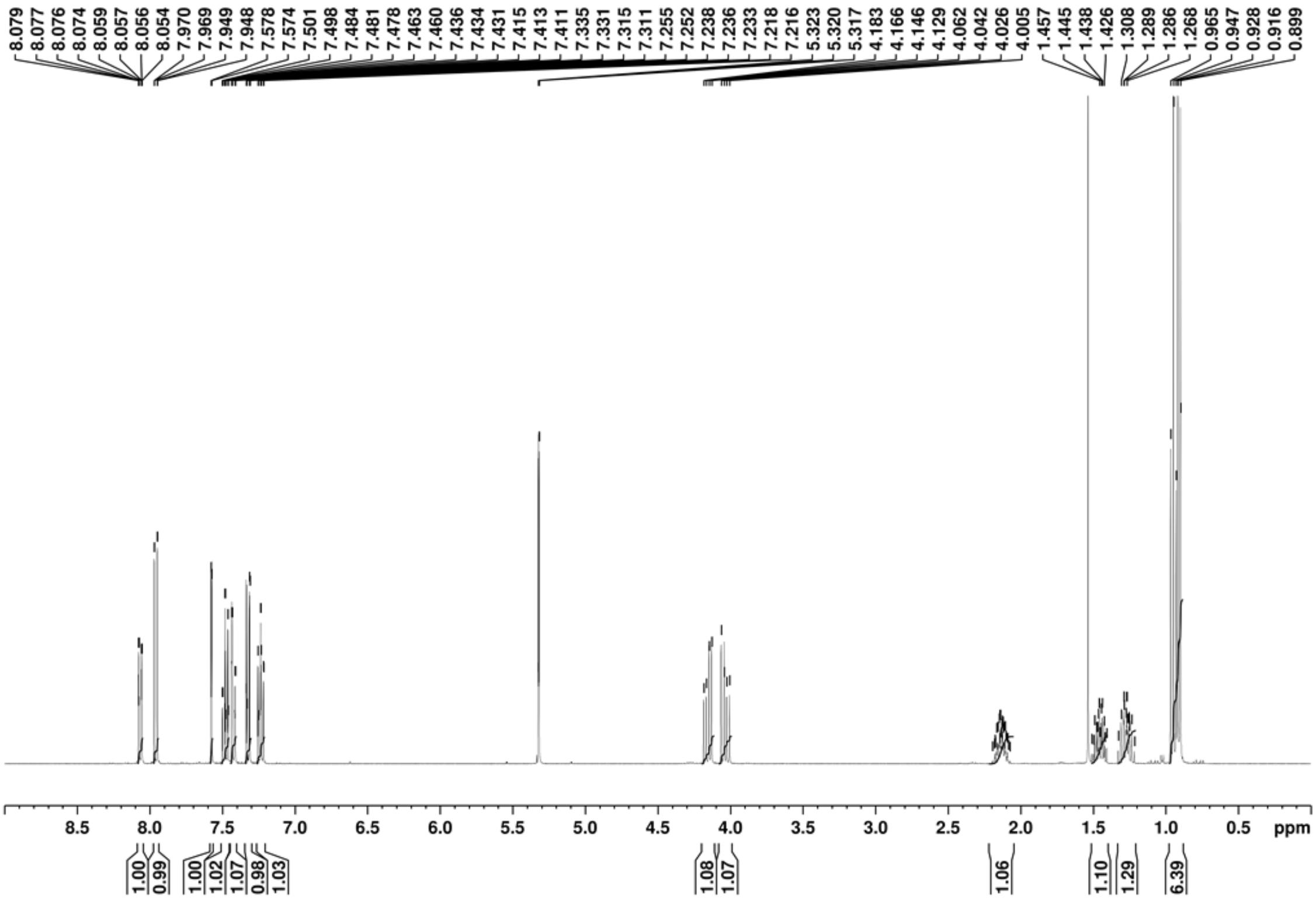

Figure S37. ^{1}H NMR (400 Hz) spectrum of compound **2** in CD_2Cl_2 at 295 K.

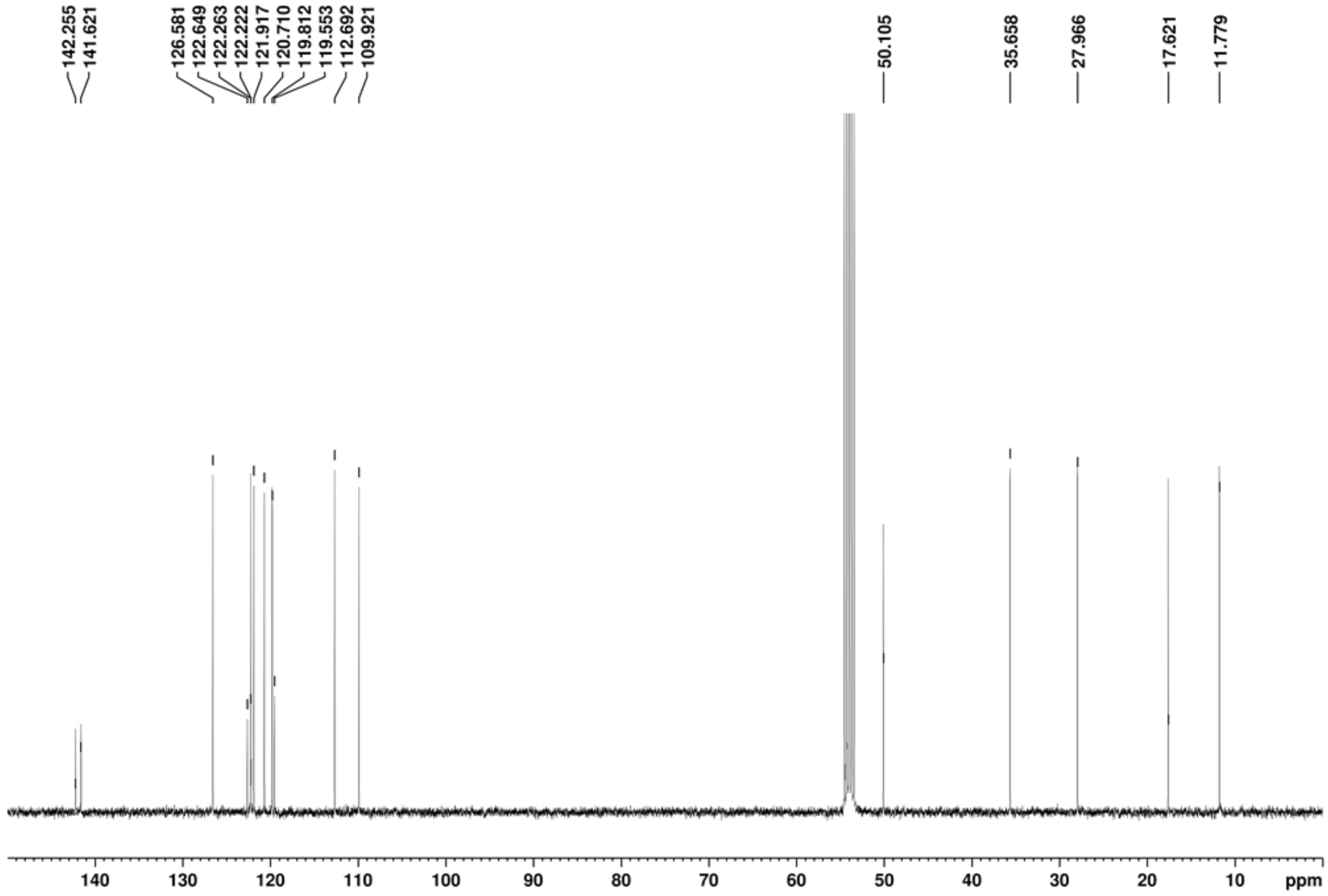

Figure S38. ^{13}C NMR (101 Hz) spectrum of compound **2** in CD_2Cl_2 at 295 K.

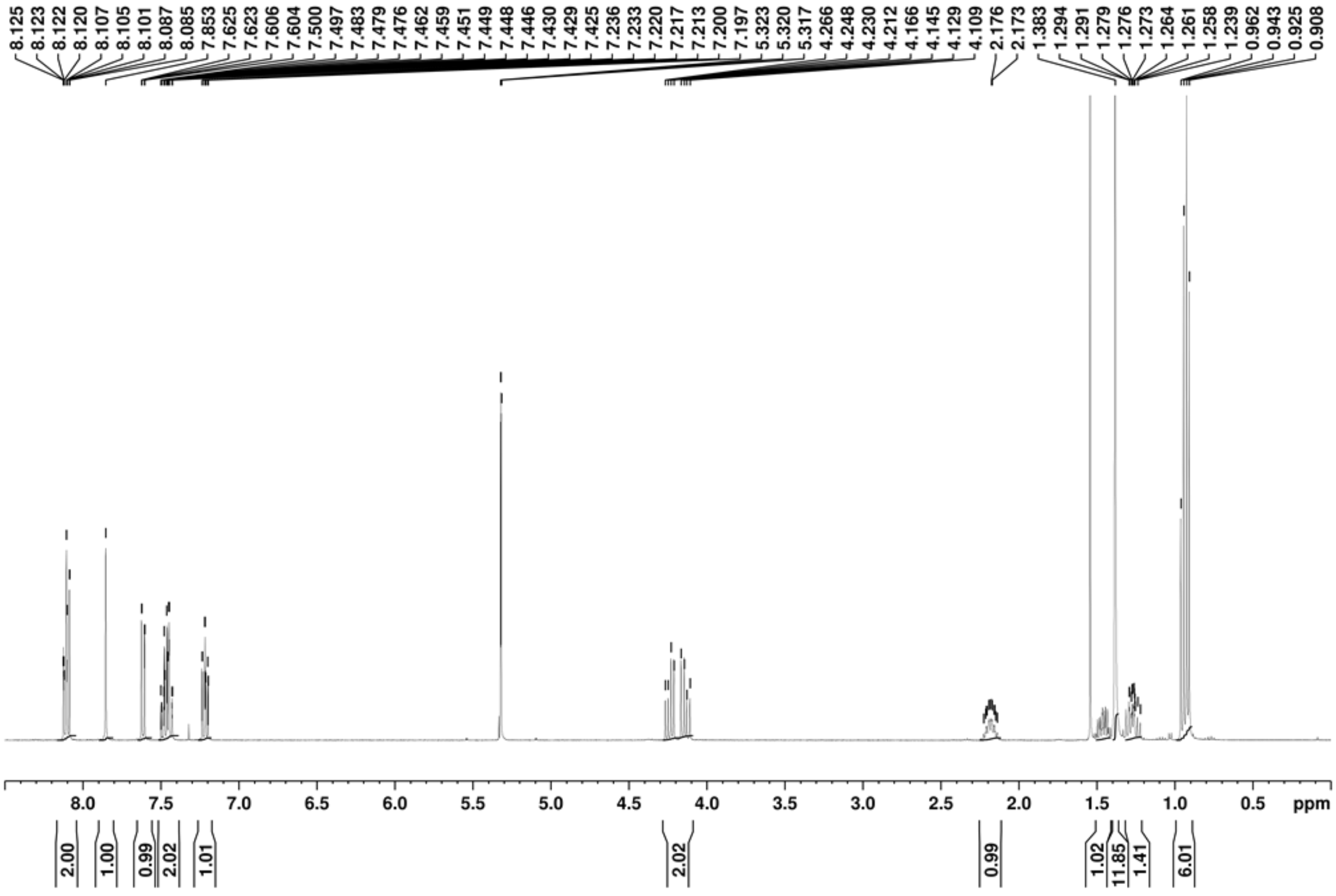

Figure S39. ^{1}H NMR (400 Hz) spectrum of compound **3** in CD_2Cl_2 at 295 K.

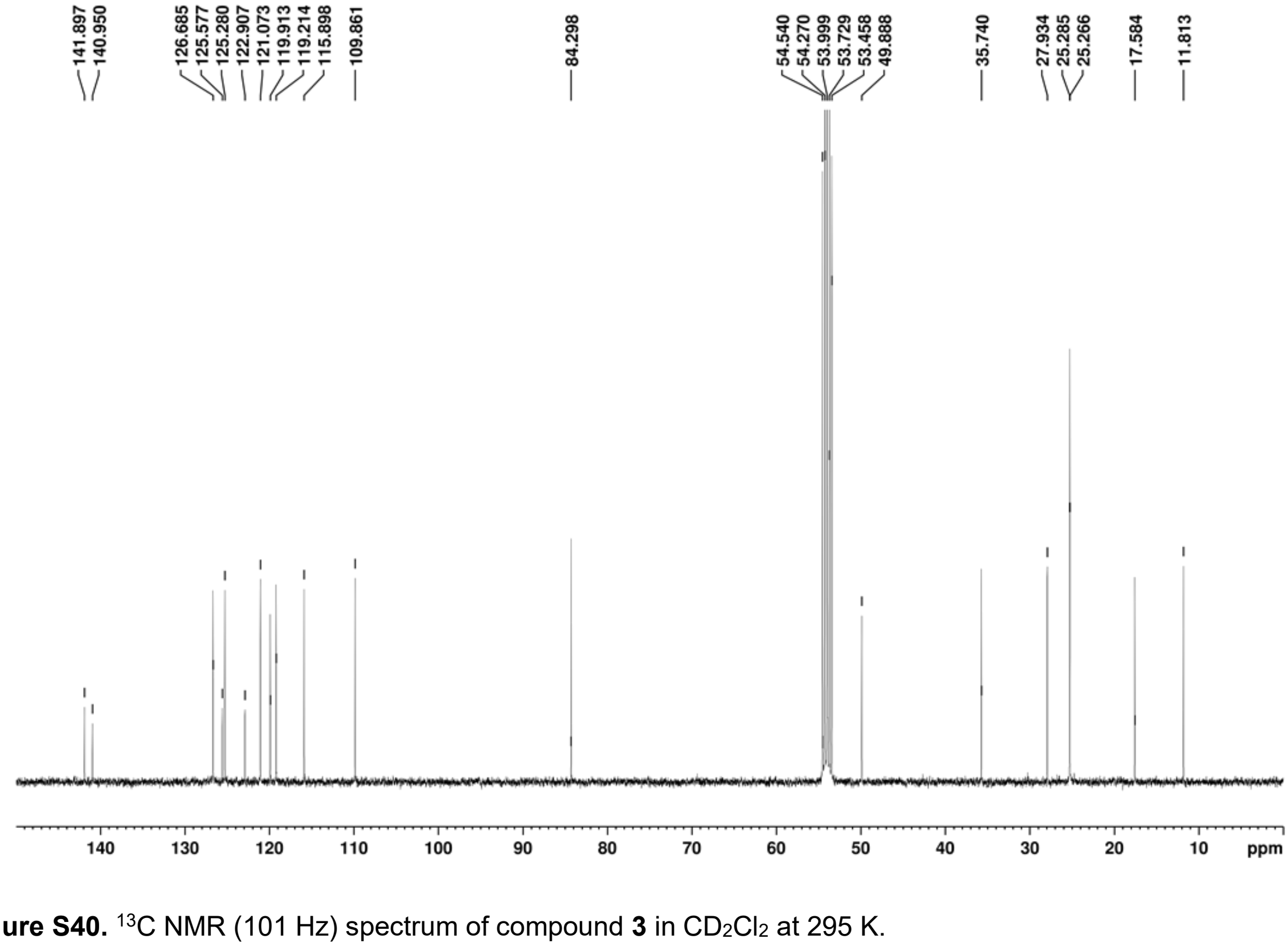

Figure S40. ^{13}C NMR (101 Hz) spectrum of compound **3** in CD_2Cl_2 at 295 K.

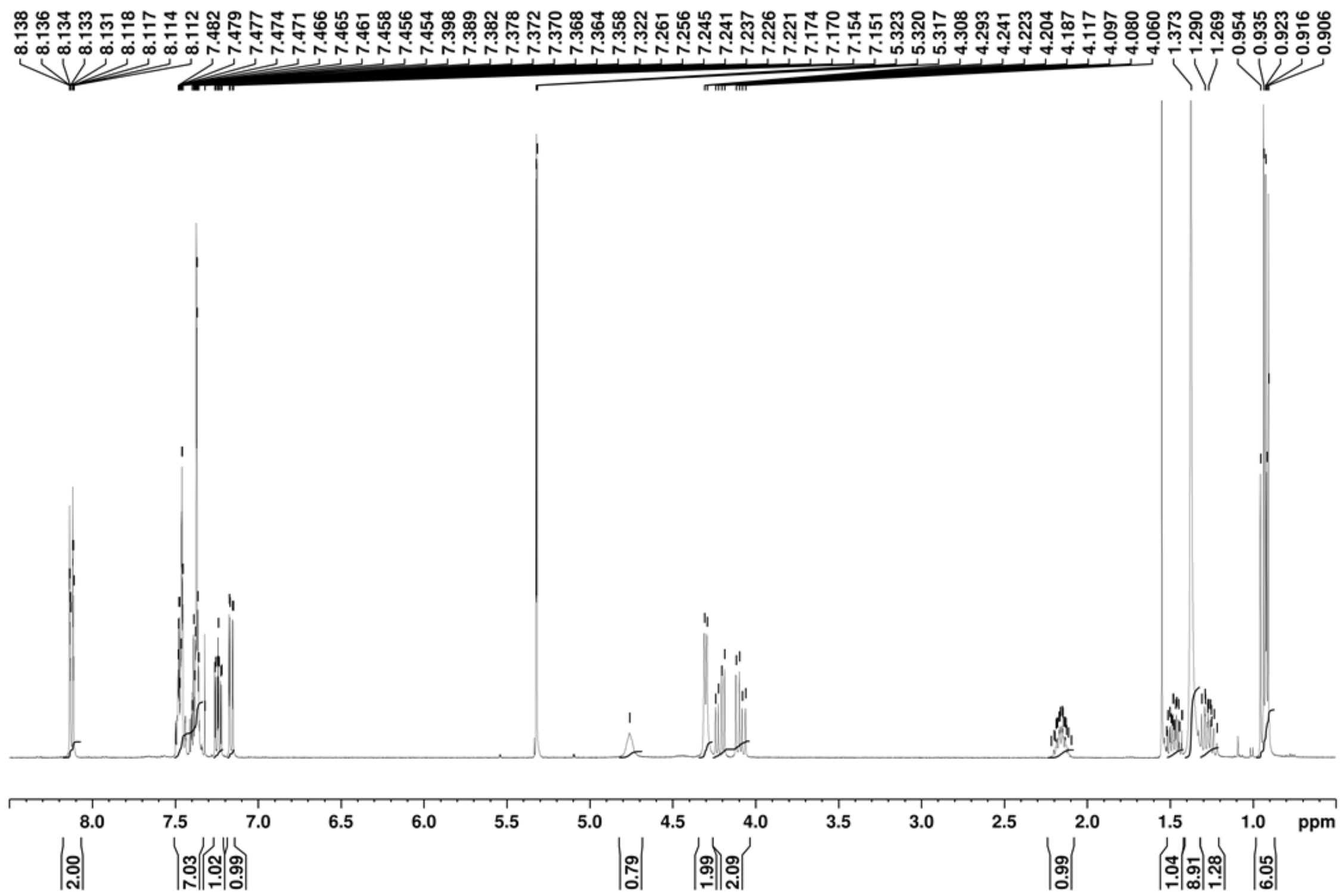

Figure S41. ^{1}H NMR (400 Hz) spectrum of compound **4** in CD_2Cl_2 at 295 K.

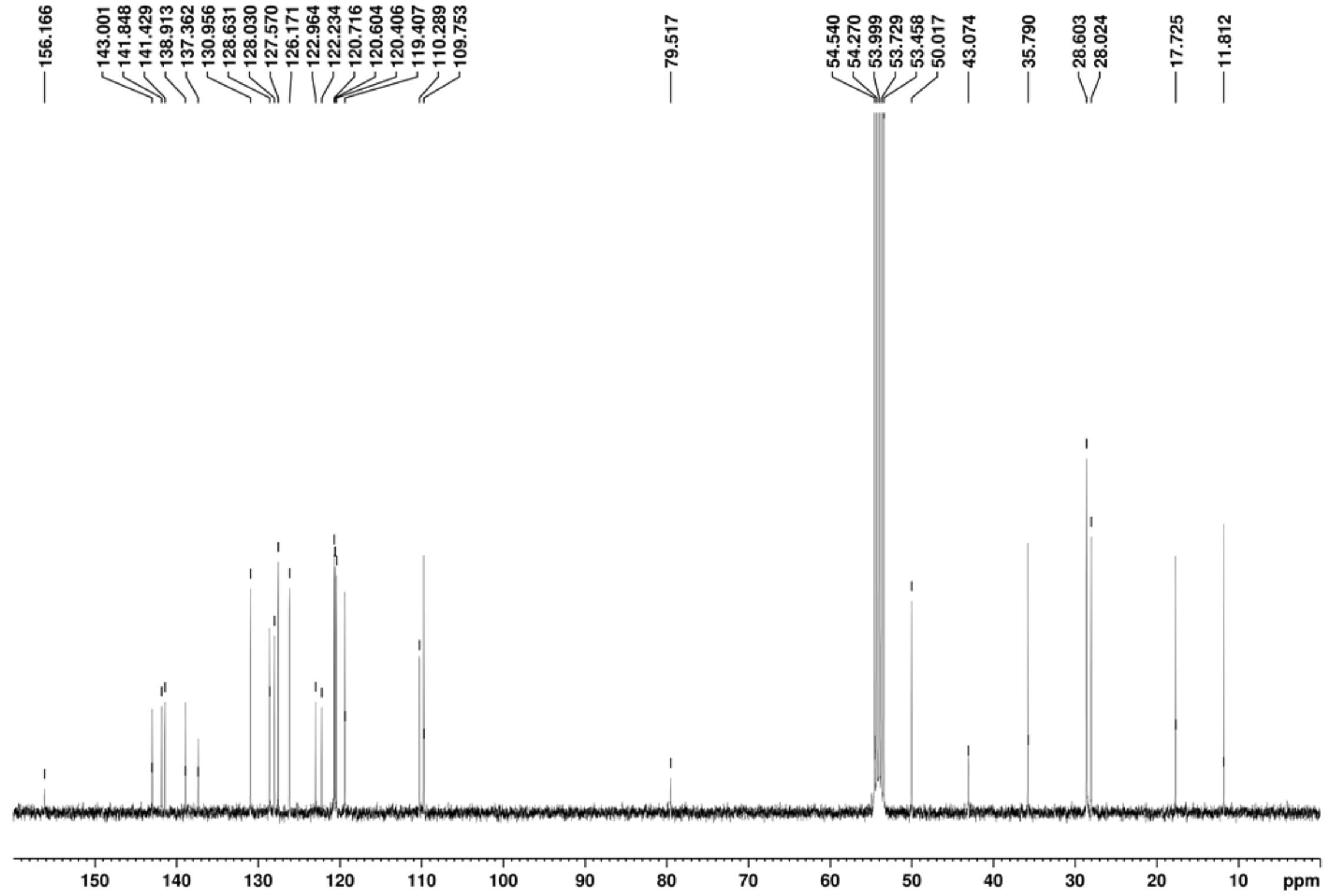

Figure S42. ^{13}C NMR (101 Hz) spectrum of compound **4** in CD_2Cl_2 at 295 K.

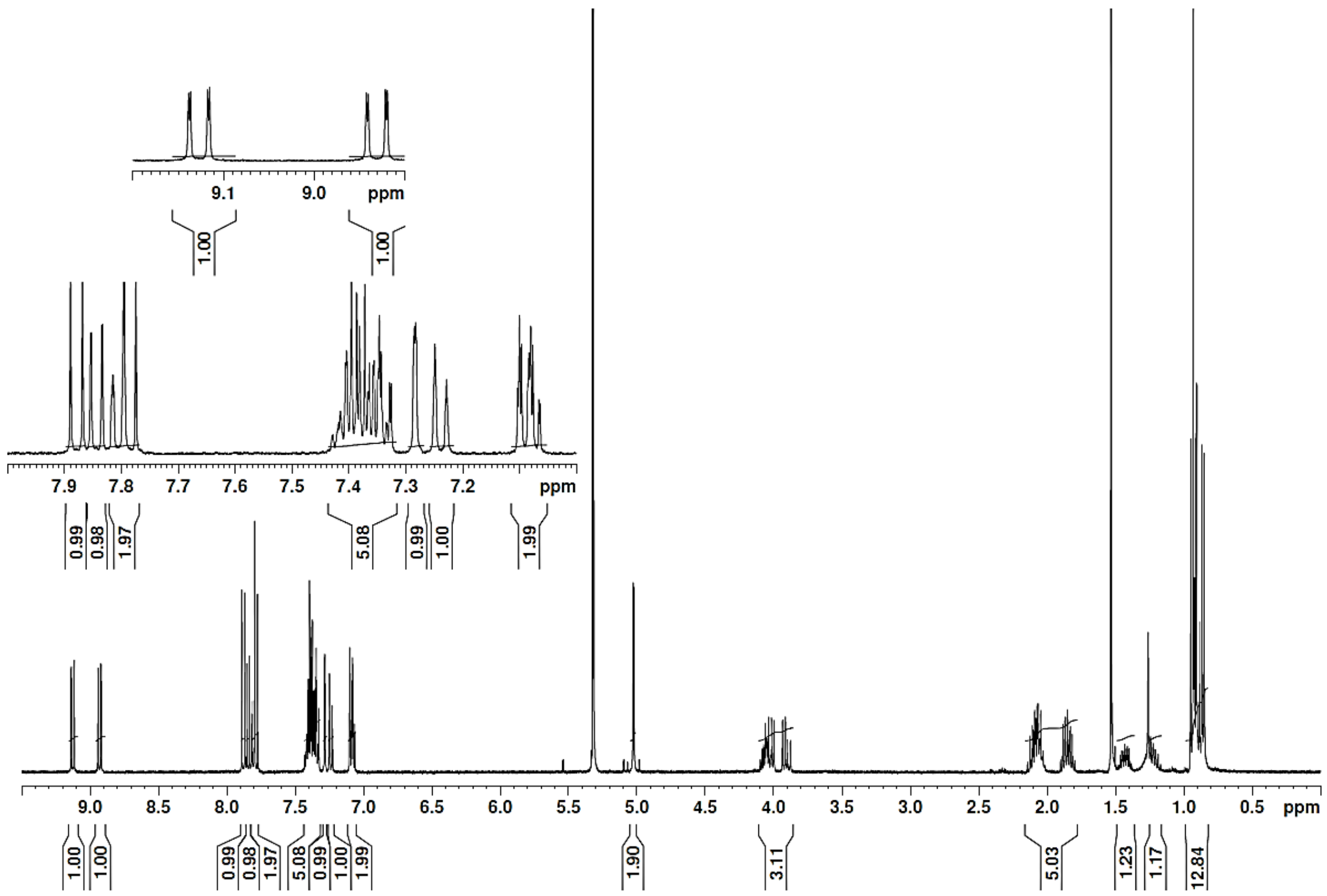

Figure S43. ^{13}C NMR (101 Hz) spectrum of **NBICz–F** in CD_2Cl_2 at 295 K.

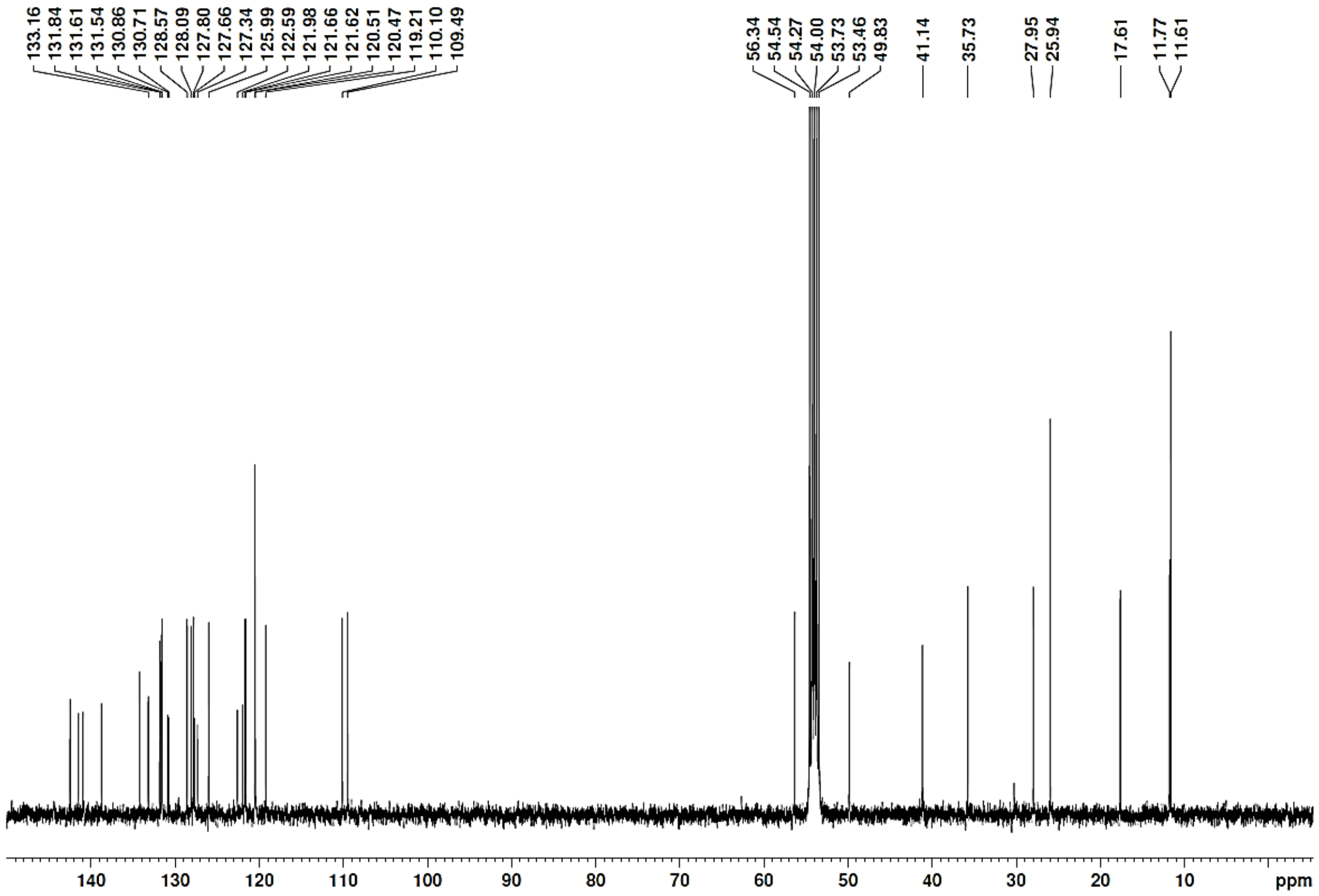

Figure S44. ^{13}C NMR (101 Hz) spectrum of **NBICz–F** in CD_2Cl_2 at 295 K.

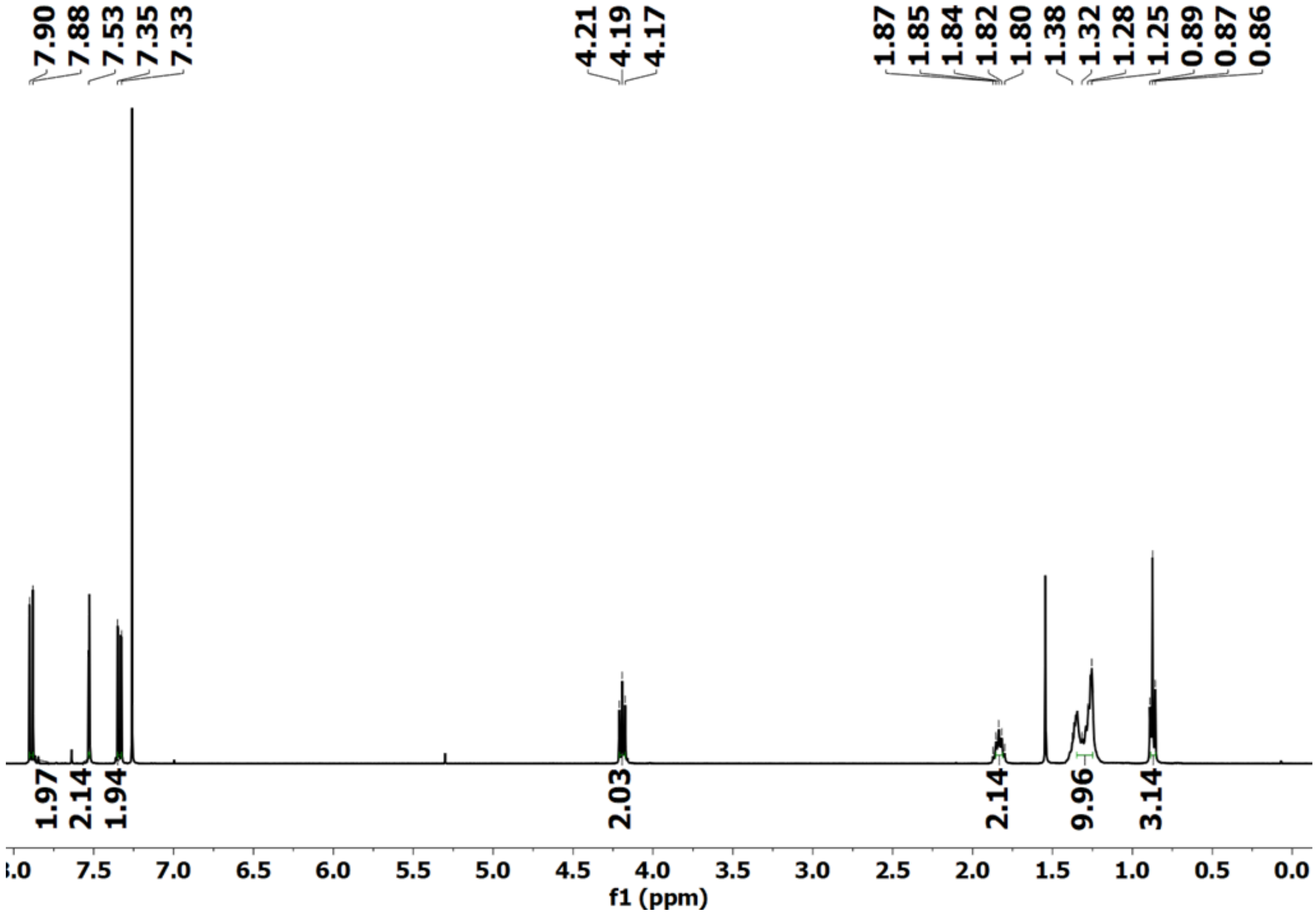

Figure S45. ^{1}H NMR (400 Hz) spectrum of compound **9** in $CDCl_3$ at 295 K.

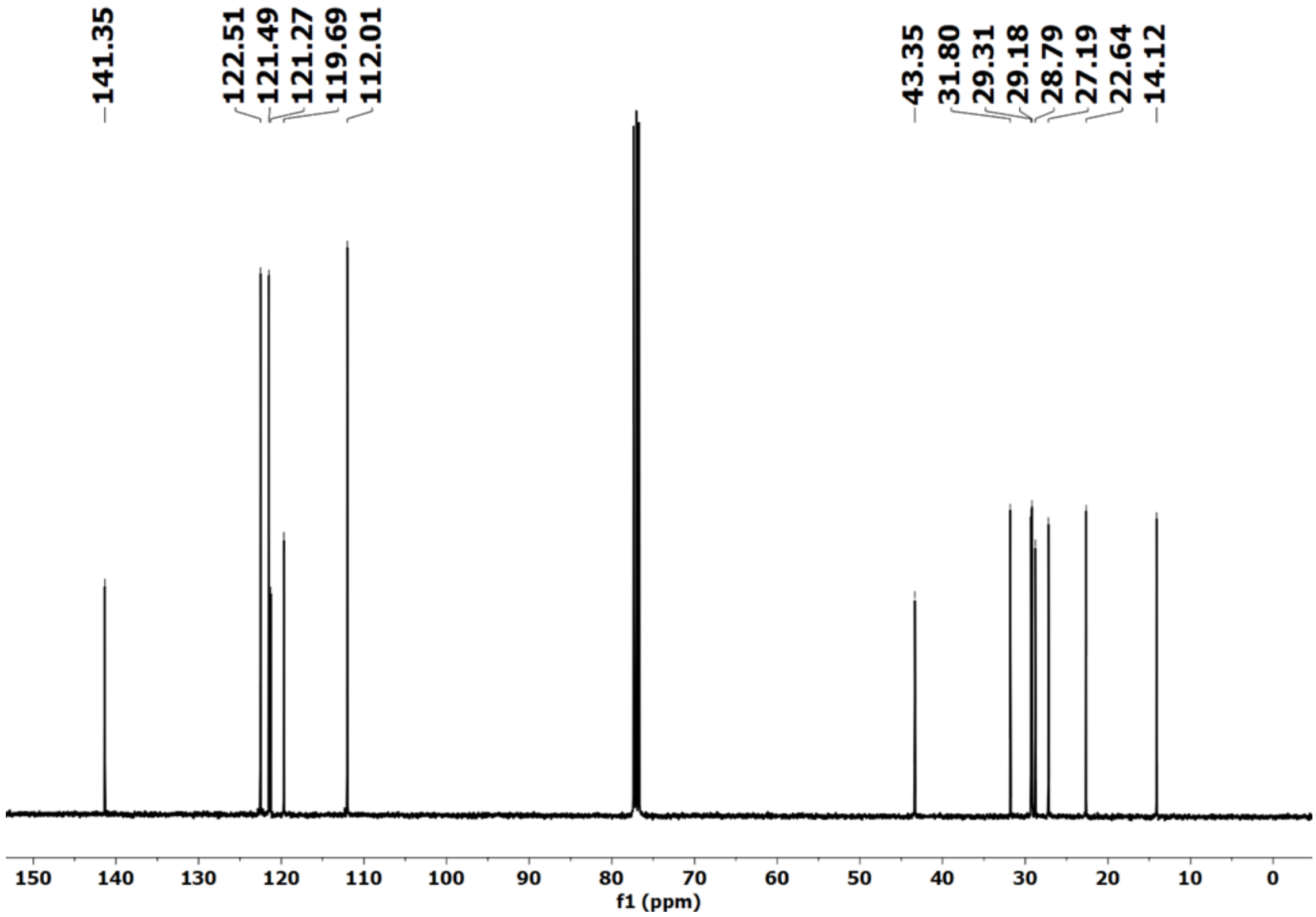

Figure S46. ^{13}C NMR (101 Hz) spectrum of compound **9** in $CDCl_3$ at 295 K.

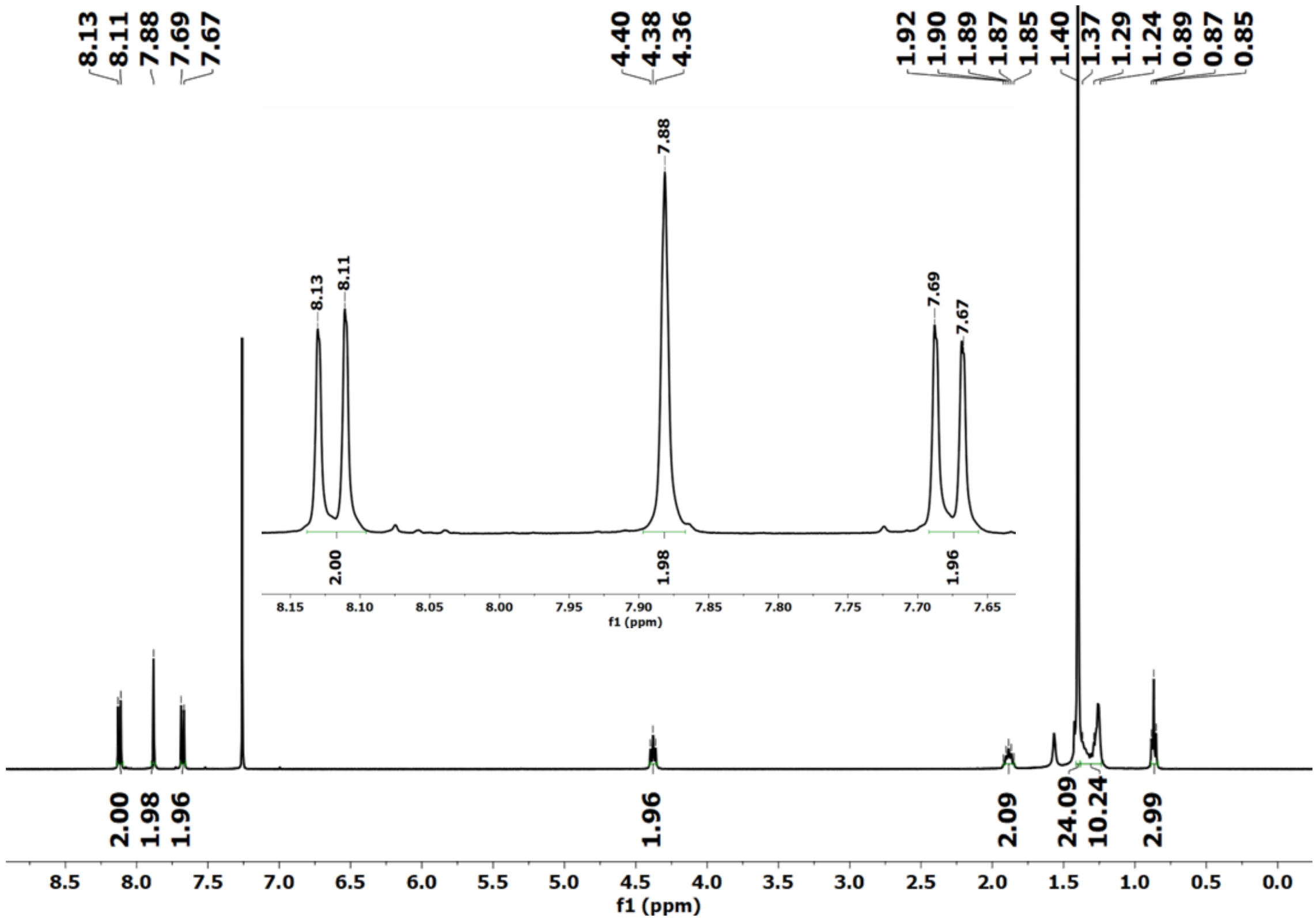

Figure S47. ^{1}H NMR (400 Hz) spectrum of compound **10** in $CDCl_3$ at 295 K.

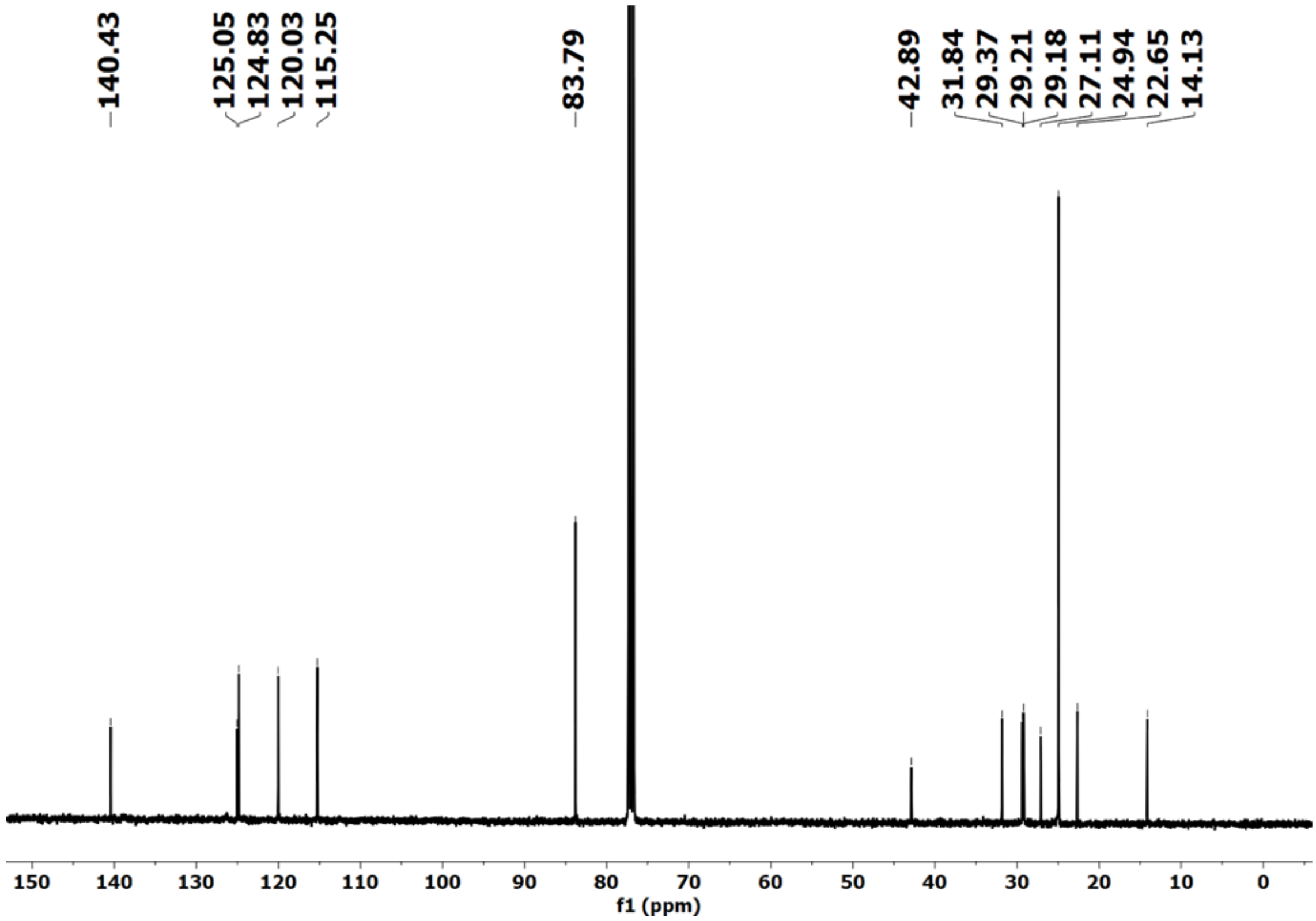

Figure S48. ^{13}C NMR (101 Hz) spectrum of compound **10** in $CDCl_3$ at 295 K.

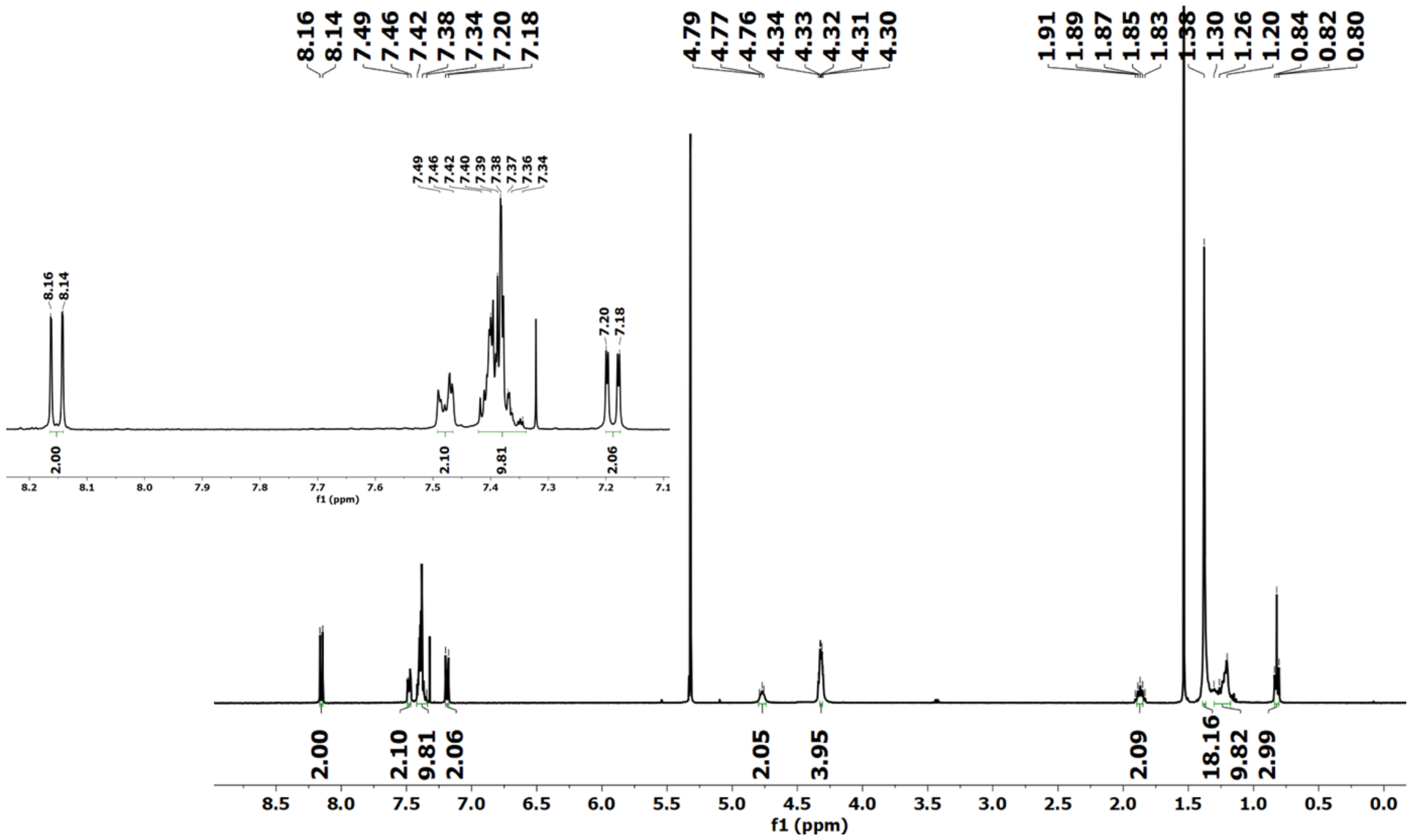

Figure S49. ^{1}H NMR (400 Hz) spectrum of compound **11** in CD_2Cl_2 at 295 K.

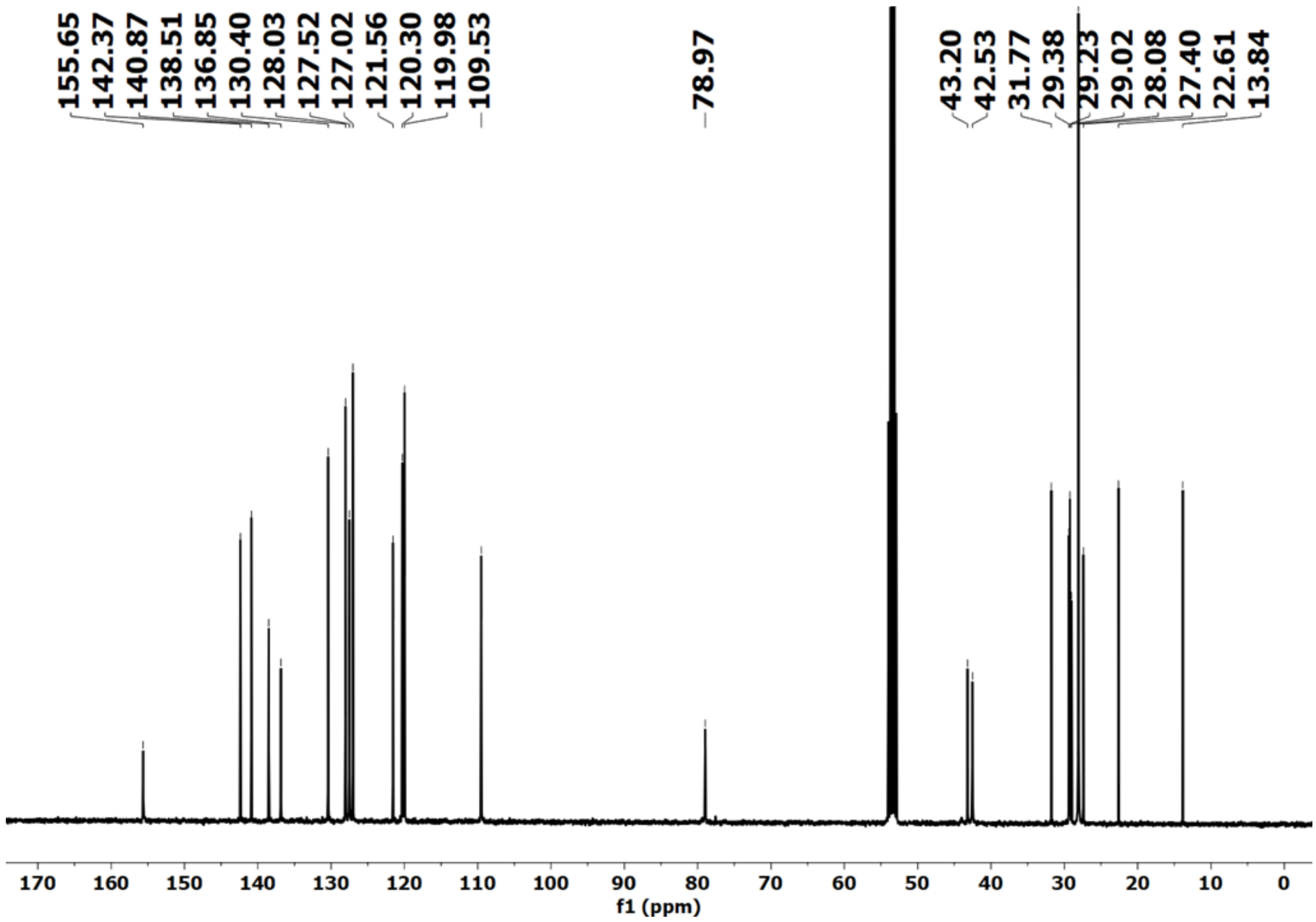

Figure S50. ^{13}C NMR (101 Hz) spectrum of compound **11** in CD_2Cl_2 at 295 K.

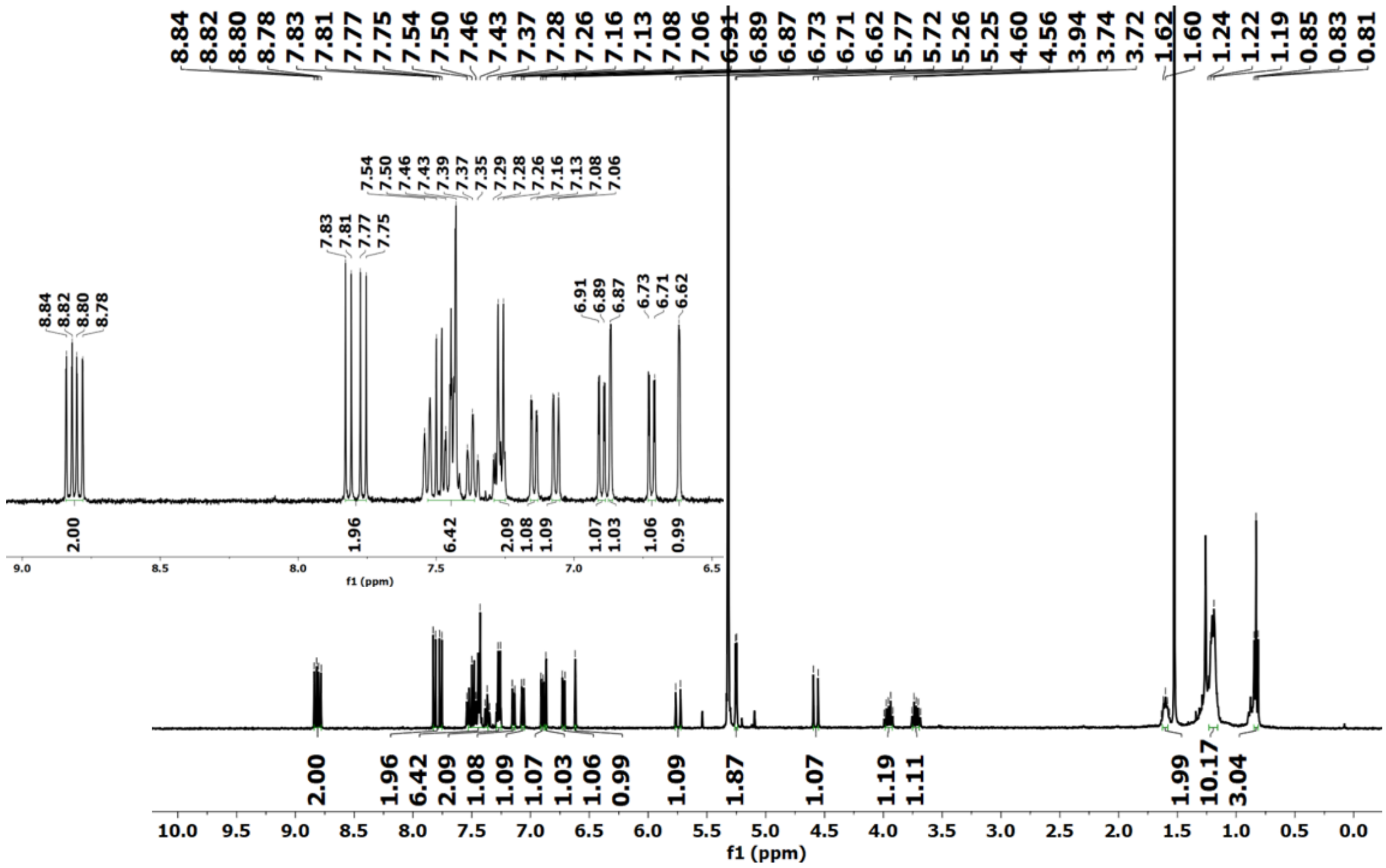

Figure S51. ^{1}H NMR (400 Hz) spectrum of racemic **NBICz–Cy** in CD_2Cl_2 at 295 K.

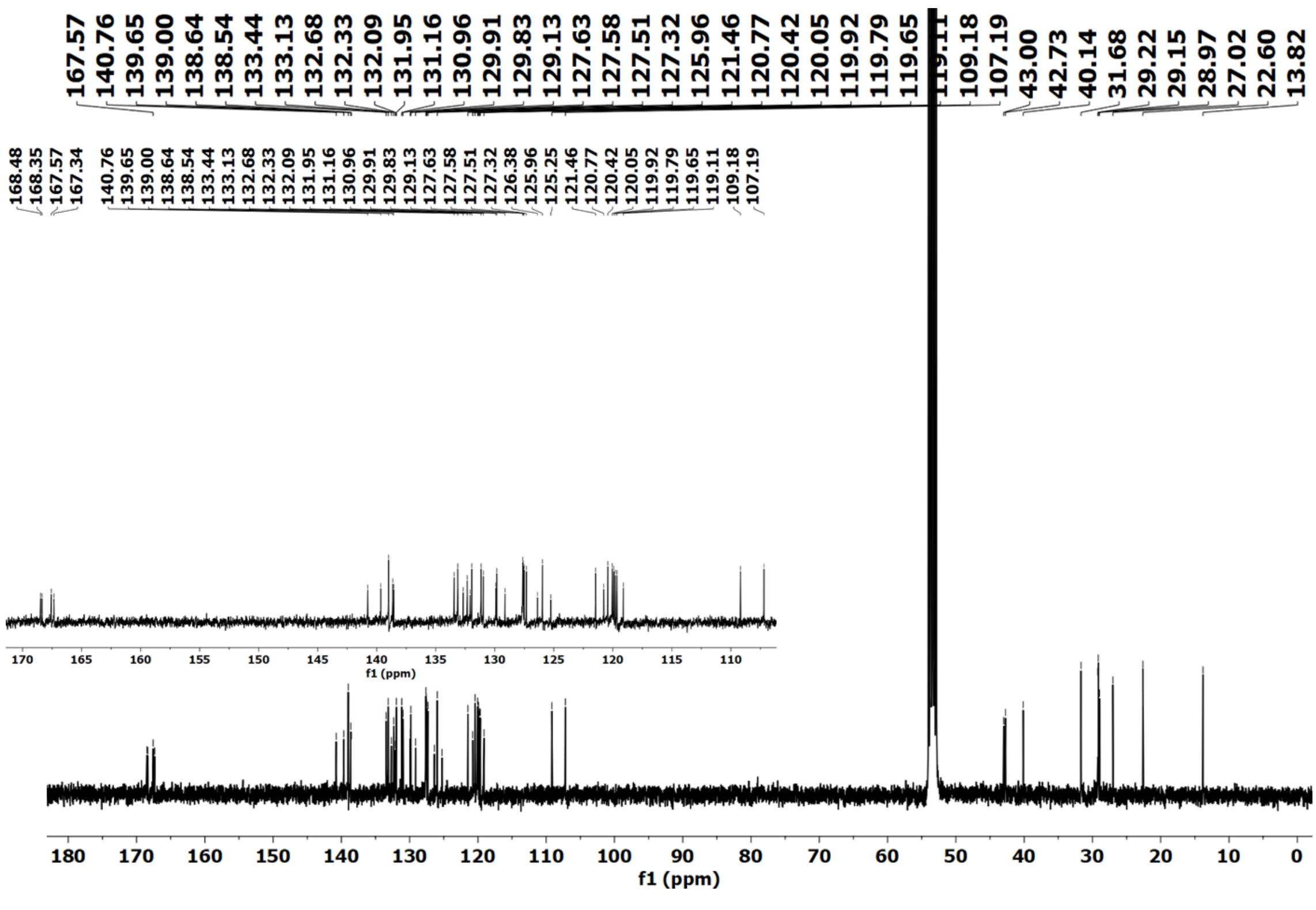

Figure S52. ^{13}C NMR (101 Hz) spectrum of racemic **NBICz–Cy** in CD_2Cl_2 at 295 K.

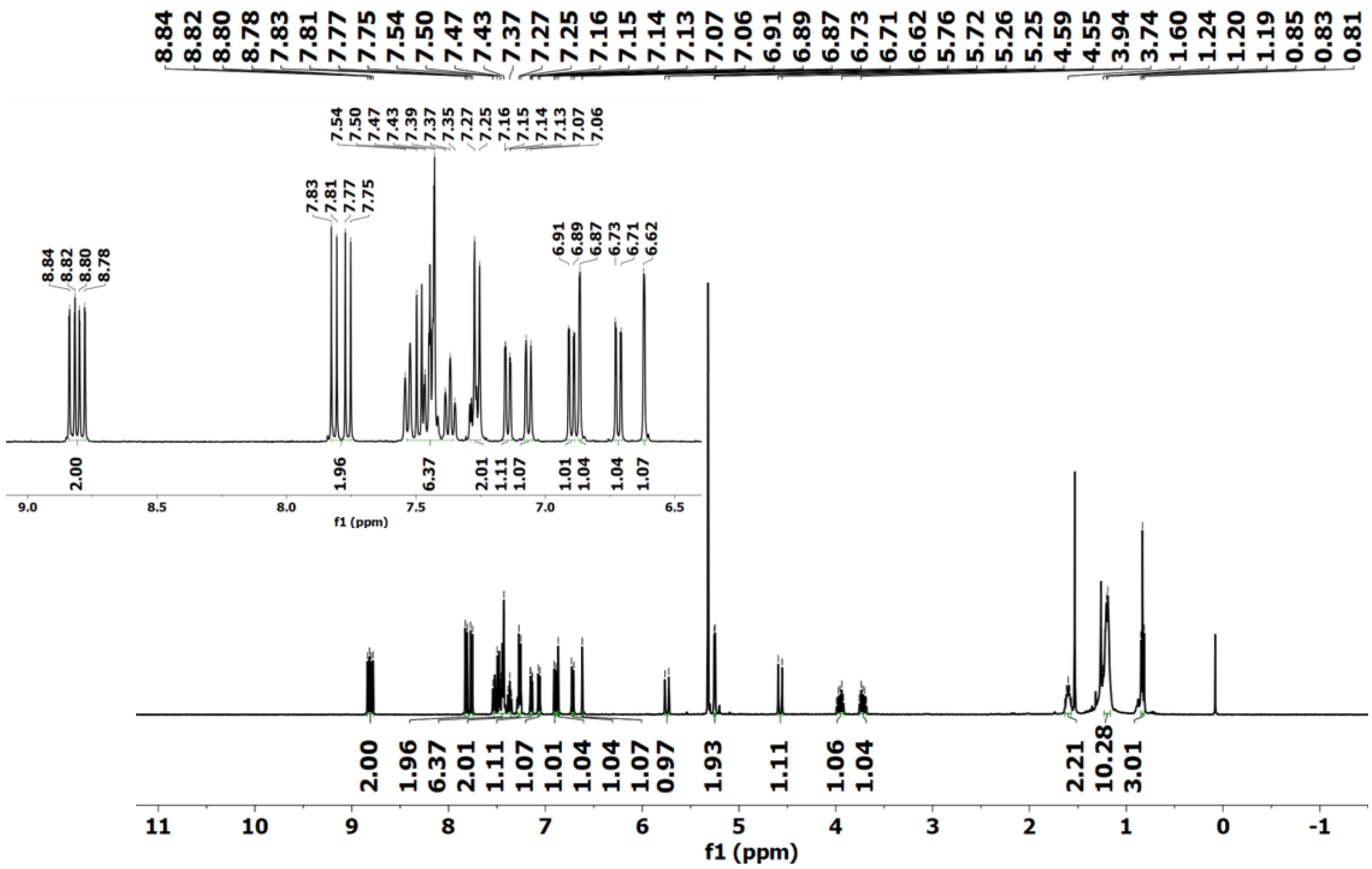

Figure S53. ^{1}H NMR (400 Hz) spectrum of compound *(R)*-**NBICz–Cy** in CD_2Cl_2 at 295 K.

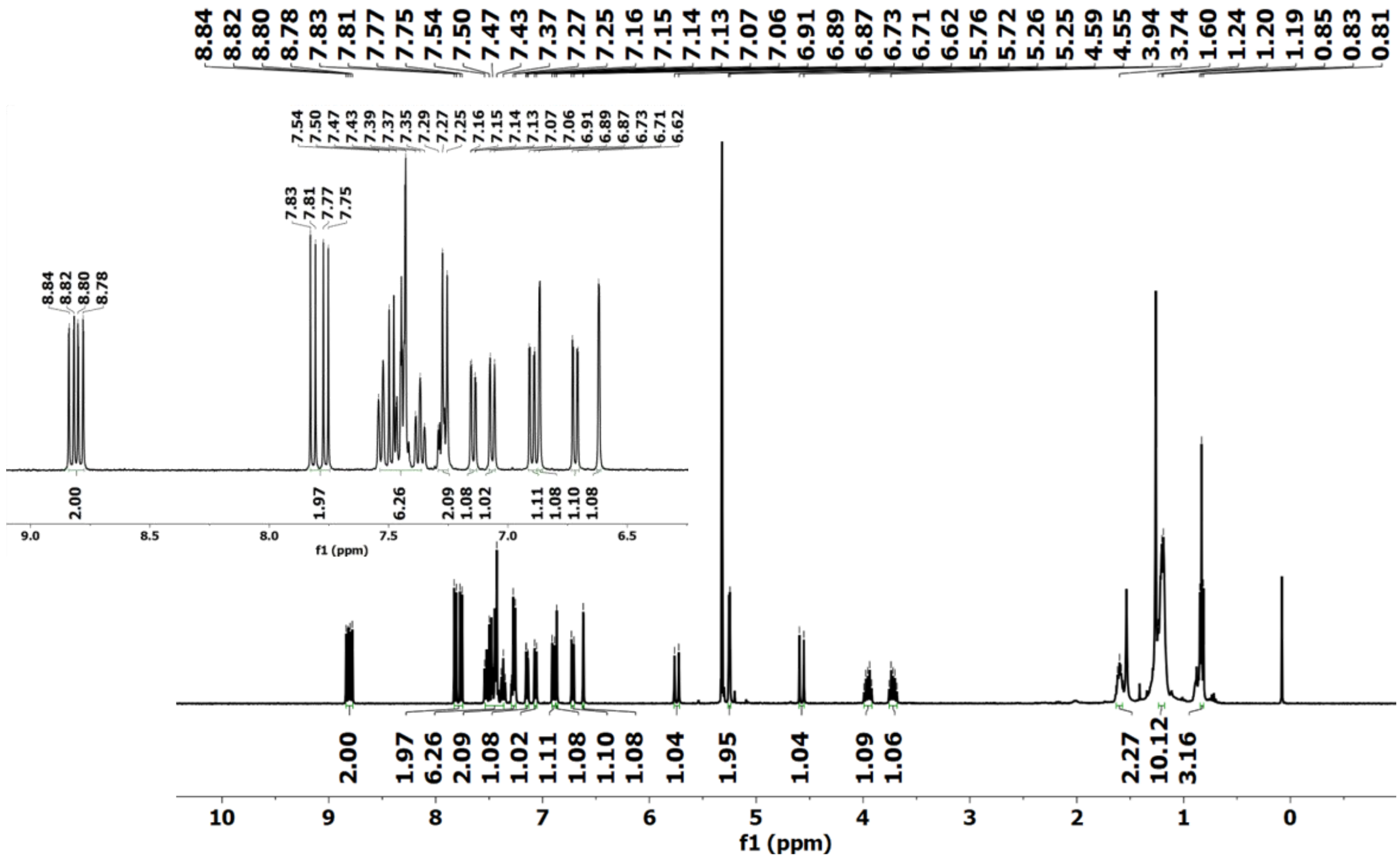

Figure S54. ^{1}H NMR (400 Hz) spectrum of compound *(S)*-**NBICz–Cy** in CD_2Cl_2 at 295 K.

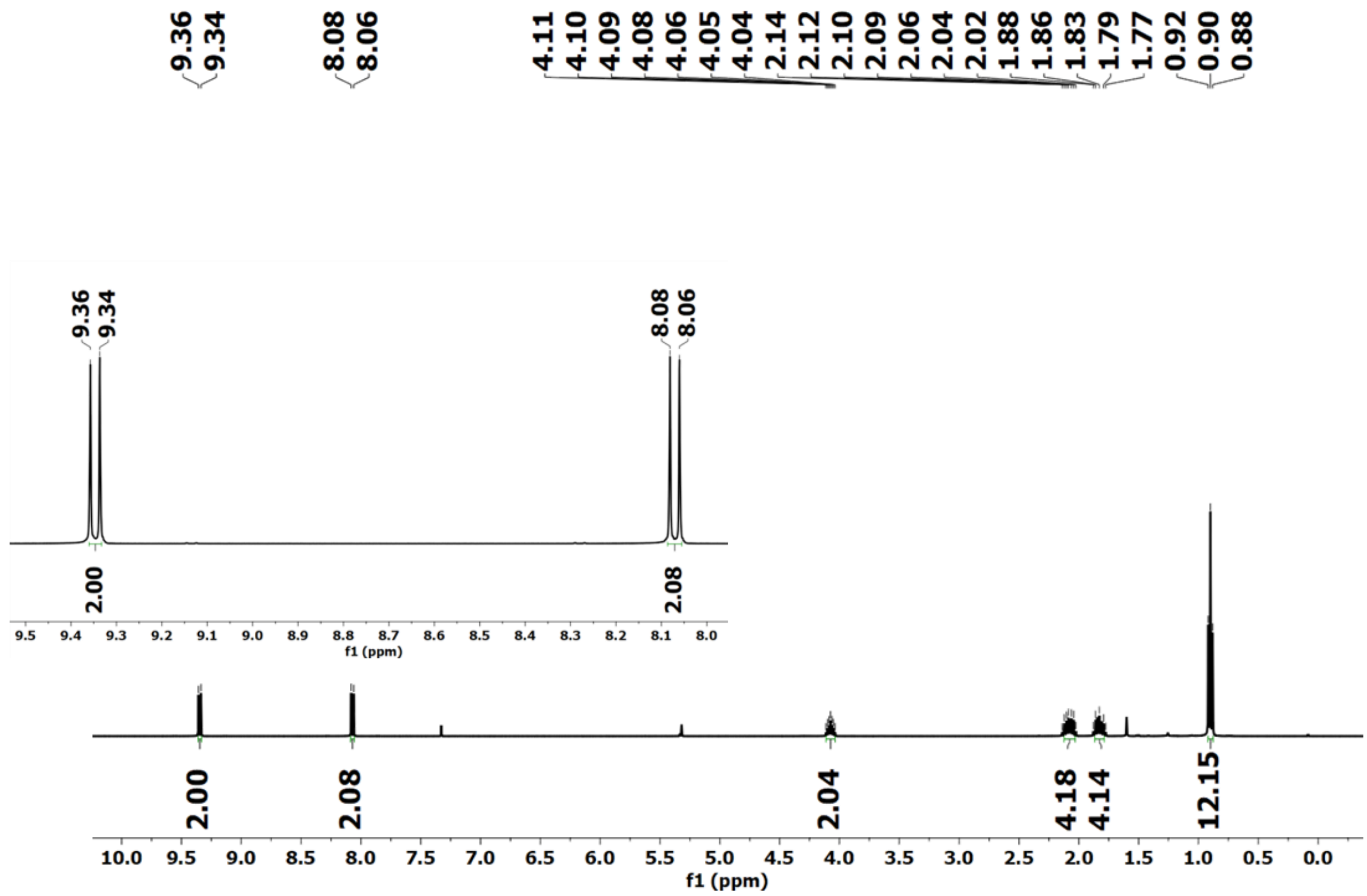

Figure S55. ^1H NMR (400 Hz) spectrum of **1,2:5,6–NBI-Ref** in CD_2Cl_2 at 295 K.

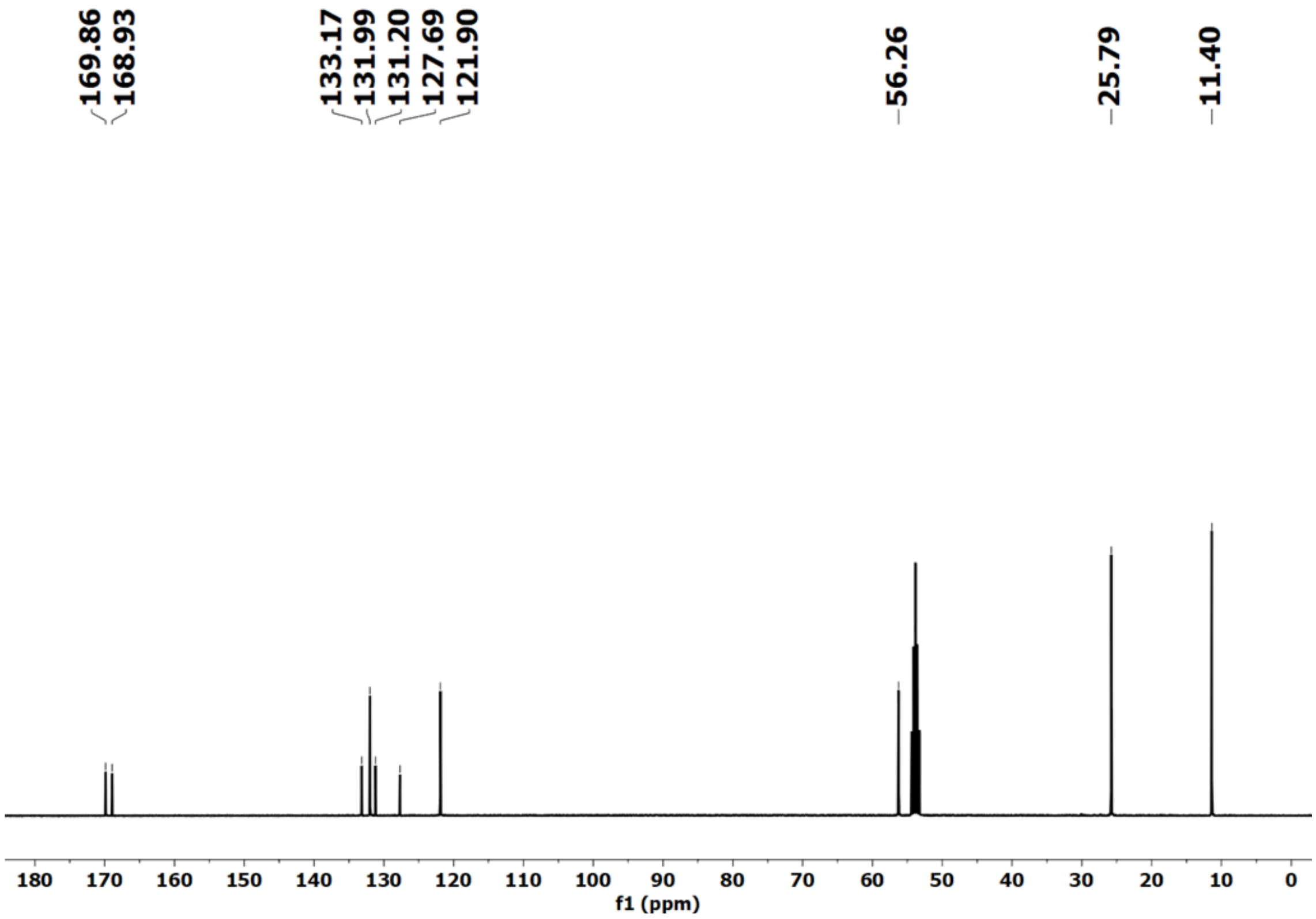

Figure S56. ^{13}C NMR (101 Hz) spectrum of **1,2:5,6–NBI-Ref** in CD_2Cl_2 at 295 K.

8. Mass Spectrometry:

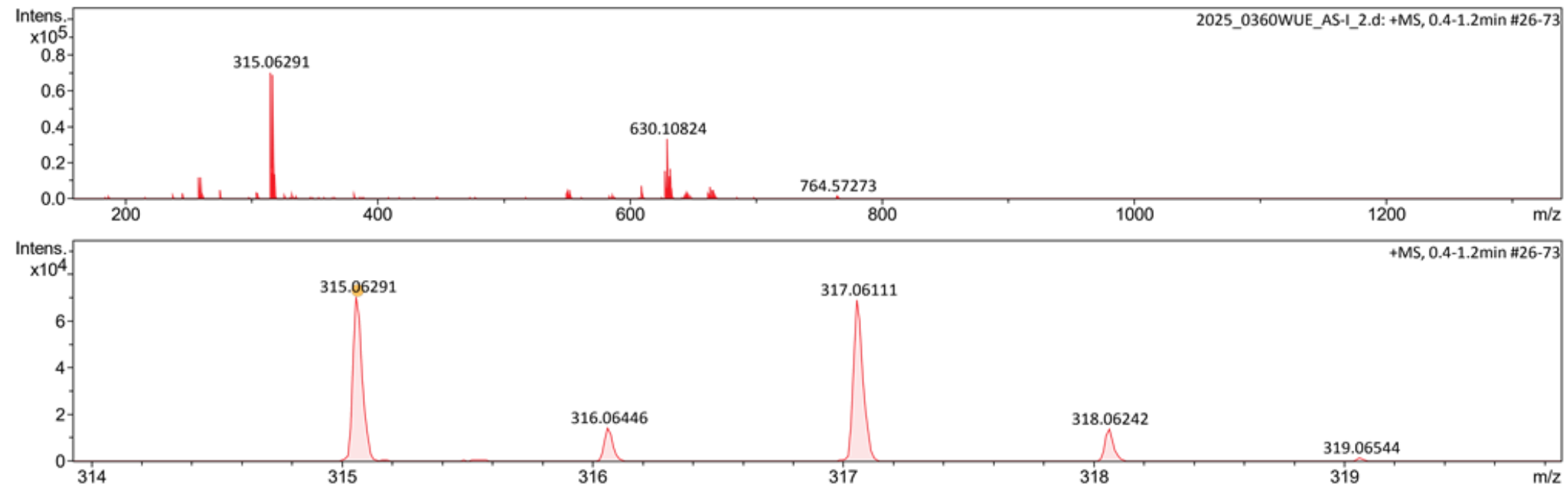

Meas. m/z	#	Ion Formula	m/z	err [ppm]	mSigma	# mSigma	Score	rdb	e¯ Conf	N-Rule
315.06291	1	C17H18BrN	315.06226	-2.07	7.4	1	100.00	9.0	odd	ok

Figure S57. HRMS-APCI data of compound **2**.

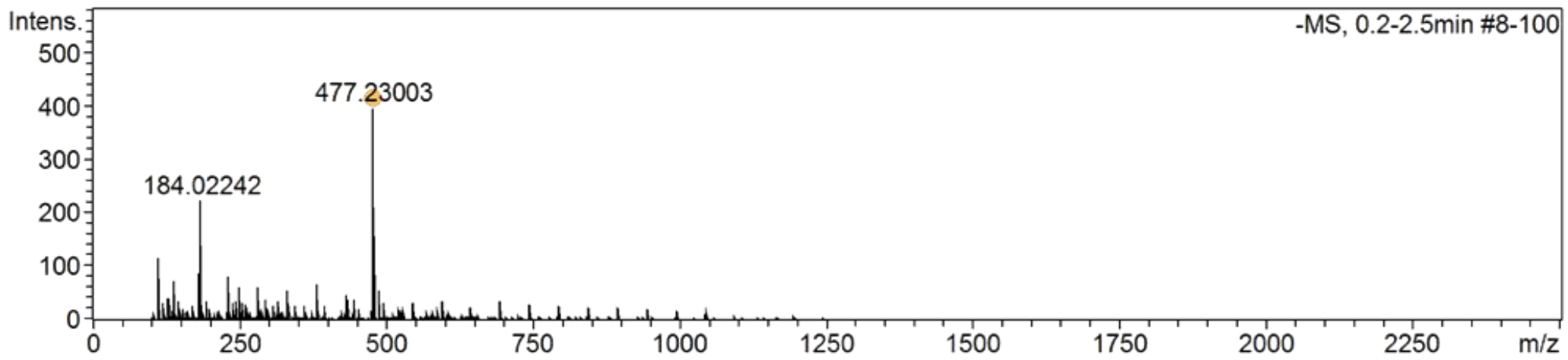

Meas. m/z	#	Ion Formula	m/z	err [ppm]	mSigma	# mSigma	Score	rdb	e¯ Conf	N-Rule
477.23003	1	C29H34ClN2O2	477.23143	2.94	51.2	1	100.00	13.5	even	ok

Figure S58. HRMS-ESI data of compound **3**.

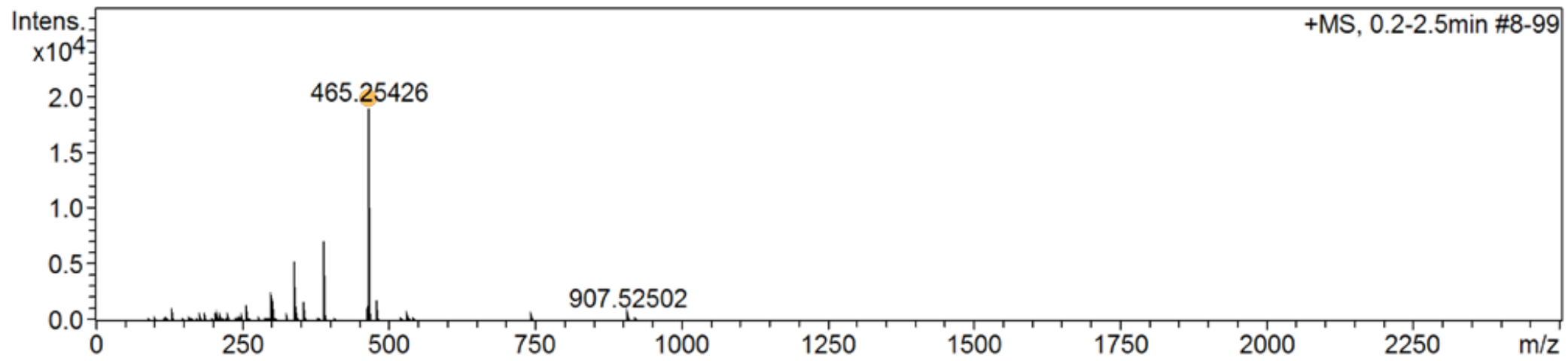

Meas. m/z	#	Ion Formula	m/z	err [ppm]	mSigma	# mSigma	Score	rdb	e¯ Conf	N-Rule
465.25426	1	C29H34N2NaO2	465.25125	-6.48	45.5	1	100.00	13.5	even	ok

Figure S59. HRMS-ESI data of compound **4**.

+MS, 0.2-2.5min #8-100

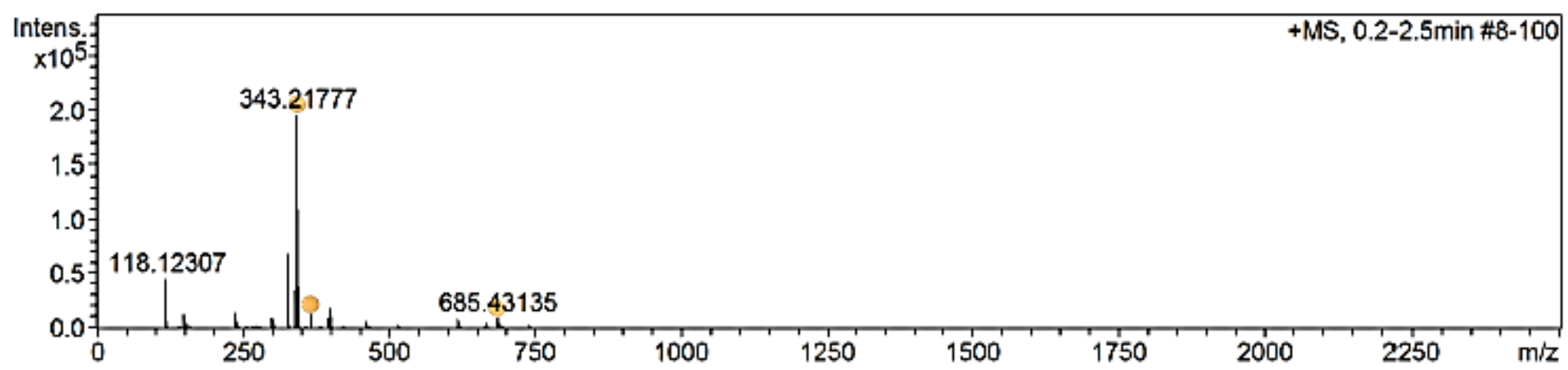

Meas. m/z	#	Ion Formula	m/z	err [ppm]	mSigma	# mSigma	Score	rdb	e¯ Conf	N-Rule
343.21777	1	C24H27N2	343.21688	-2.62	41.6	1	100.00	12.5	even	ok
365.20028	1	C24H26N2Na	365.19882	-3.99	28.7	1	100.00	12.5	even	ok
685.43135	1	C48H53N4	685.42647	-7.12	25.0	1	100.00	24.5	even	ok

Figure S60. HRMS-ESI data of compound **5**.

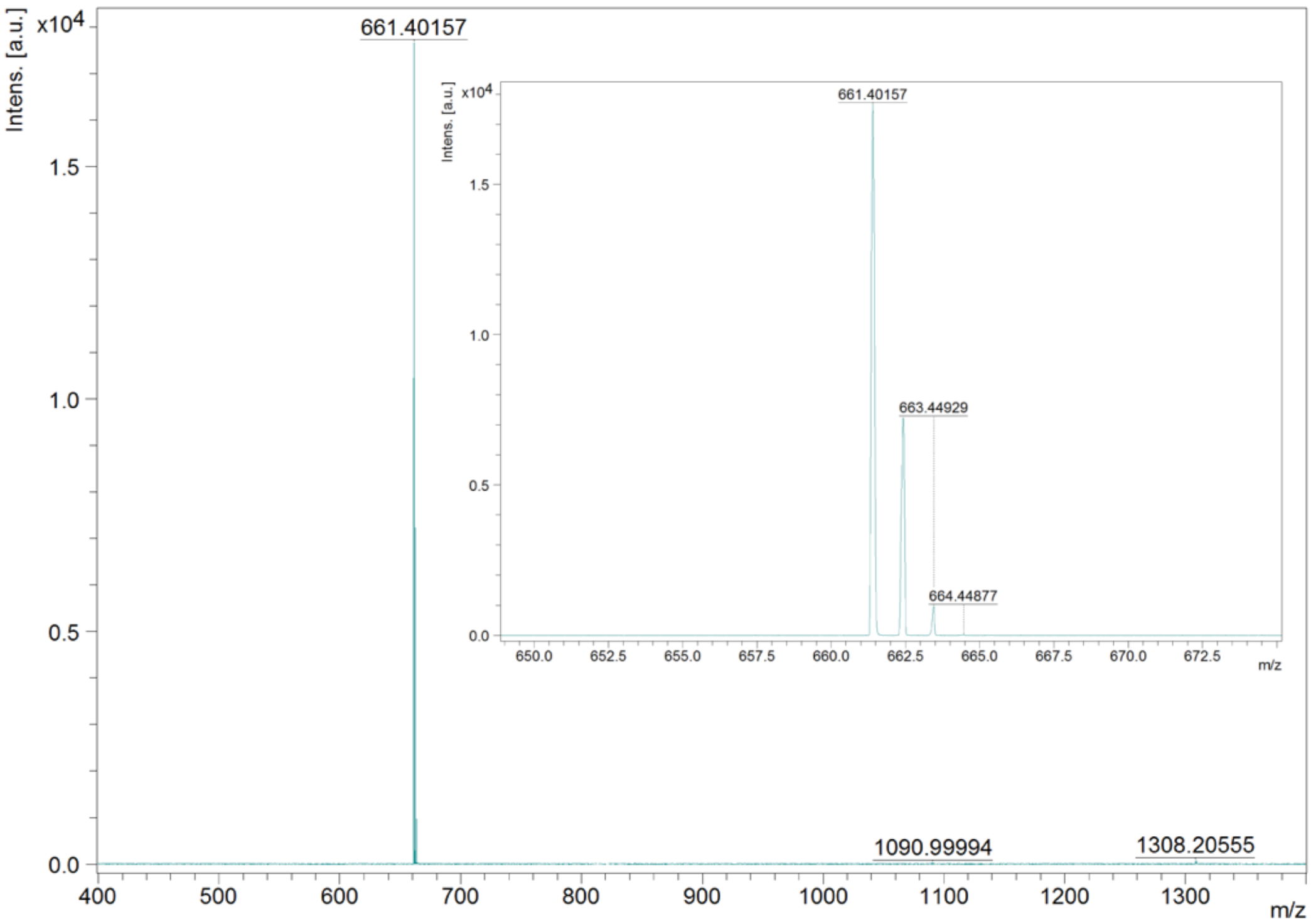

Figure S61. MALDI-TOF spectrum (negative mode, DCTB in chloroform) of **NBICz–F.**

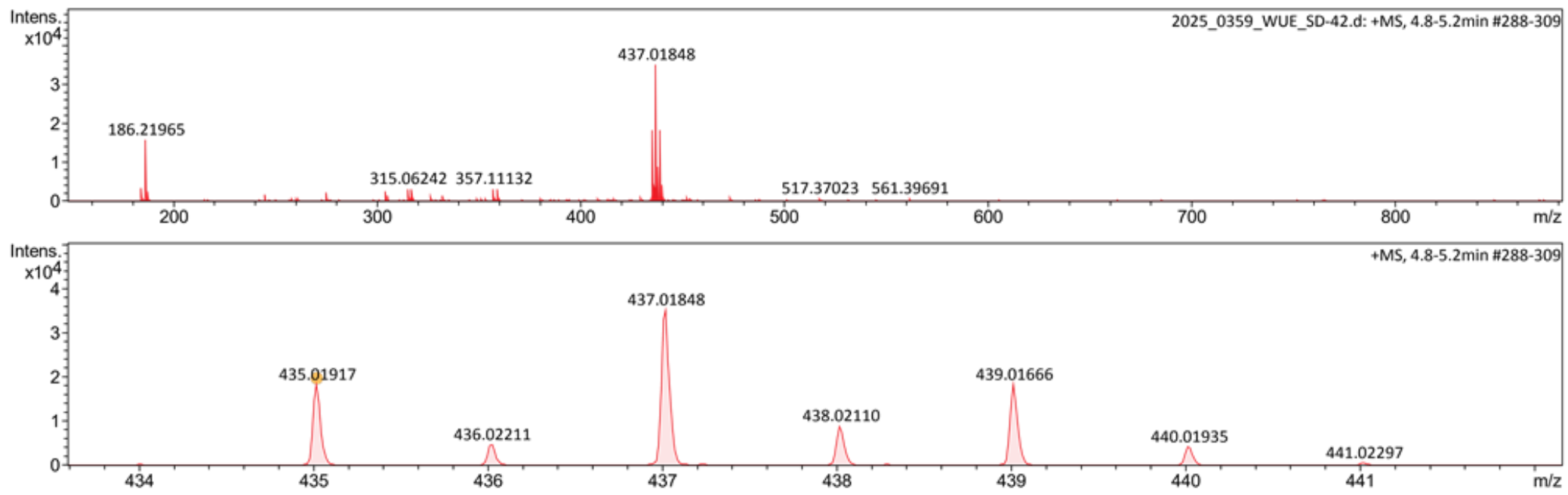

Meas. m/z	#	Ion Formula	m/z	err [ppm]	mSigma	# mSigma	Score	rdb	e⁻ Conf	N-Rule
435.01917	1	C20H23Br2N	435.01972	1.26	17.3	1	100.00	9.0	odd	ok

Figure S62. HRMS-APCI data of compound **9**.

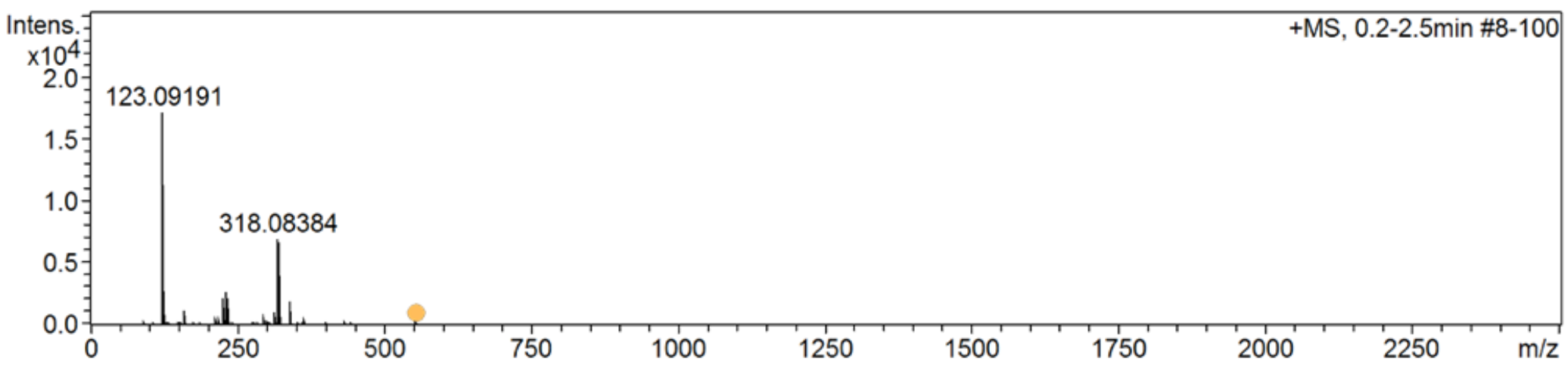

Meas. m/z	#	Ion Formula	m/z	err [ppm]	mSigma	# mSigma	Score	rdb	e⁻ Conf	N-Rule
554.35951	1	C32H47B2NNaO4	554.35834	-0.14	160.2	1	100.00	10.5	even	ok

Figure S63. HRMS-ESI data of compound **10**.

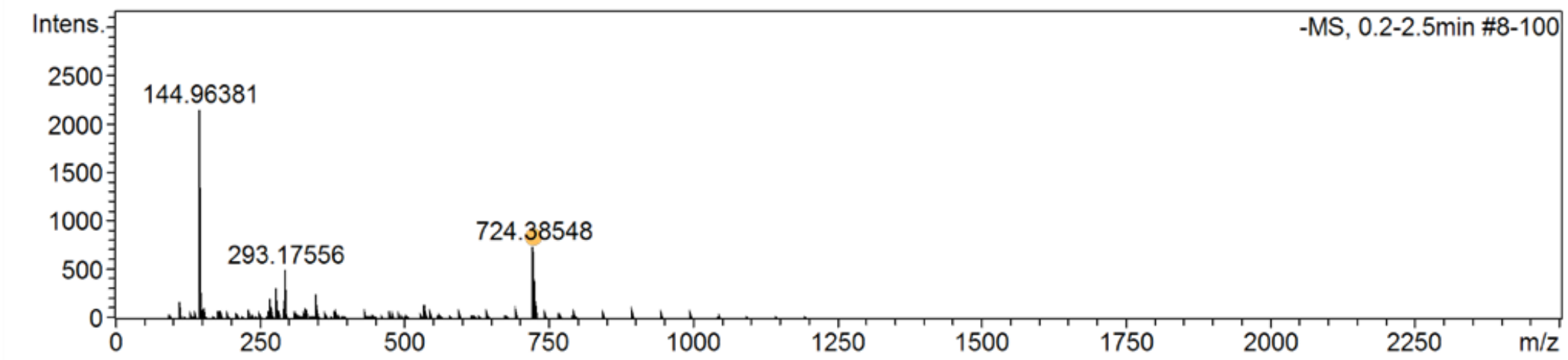

Meas. m/z	#	Ion Formula	m/z	err [ppm]	mSigma	# mSigma	Score	rdb	e¯ Conf	N-Rule
724.38548	1	C44H55ClN3O4	724.38866	4.39	32.9	1	100.00	18.5	even	ok

Figure S64. HRMS-ESI data of compound **11**.

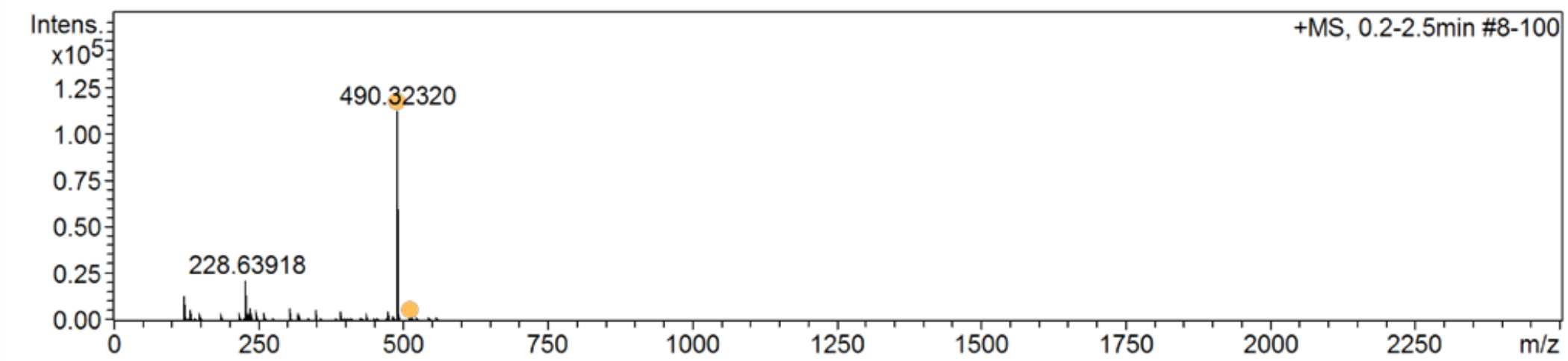

Meas. m/z	#	Ion Formula	m/z	err [ppm]	mSigma	# mSigma	Score	rdb	e¯ Conf	N-Rule
490.32320	1	C34H40N3	490.32167	-3.11	69.3	1	100.00	16.5	even	ok
512.30446	1	C34H39N3Na	512.30362	-1.64	43.1	1	100.00	16.5	even	ok

Figure S65. HRMS-ESI data of compound **12**.

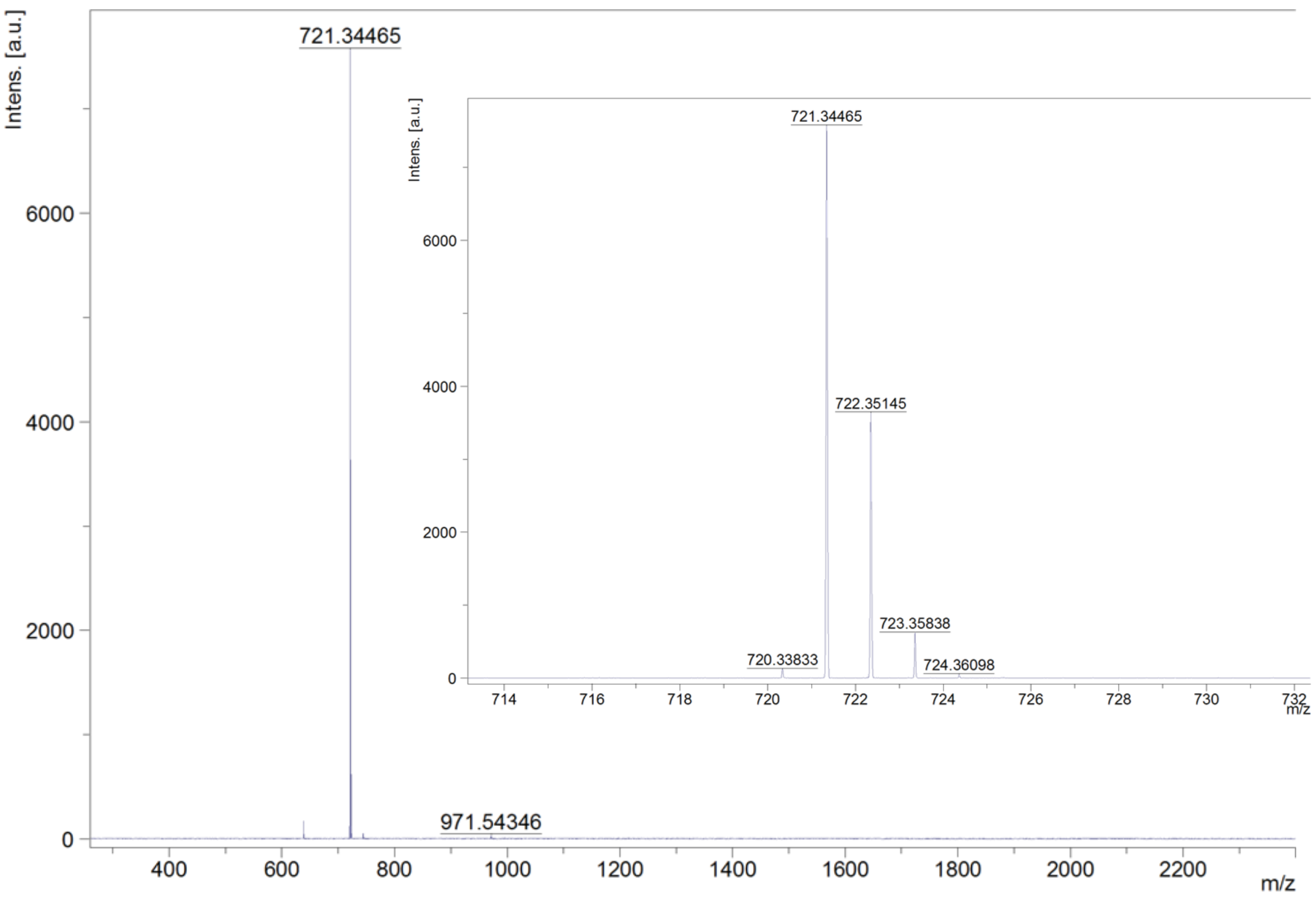

Figure S66. MALDI-TOF spectrum (positive mode, DCTB in chloroform) of racemic **NBICz–Cy**.

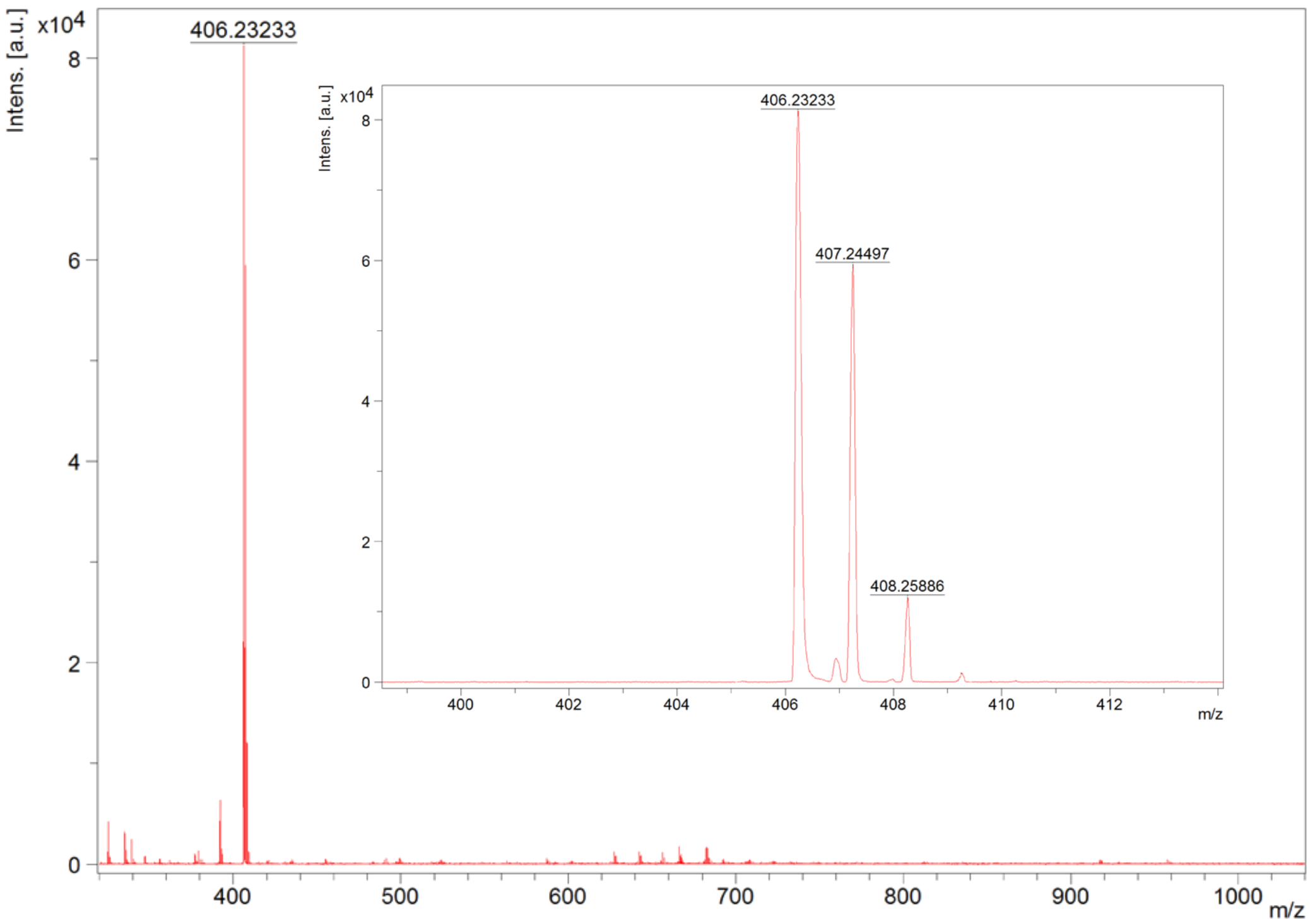

Figure S67. MALDI-TOF spectrum (negative mode, DCTB in chloroform) of **1,2:5,6–NBI-Ref**.

9. References:

[S1] J. R. Lakowicz, “Principles of Fluorescence Spectroscopy,” Kluwer Academic/Plenum Publishers, New York, 1999.

[S2] W. Kabsch, *XDS*. Acta Crystallographica Section D: Structural Biology **66**, (2010): 125–132.

[S3] G. M. Sheldrick, “SHELXT–integrated space-group and crystal-structure determination,” Acta Crystallogr. *A: Found. Adv.* **71**, (2015): 3–8.

[S4] G. M. Sheldrick, “Crystal structure refinement with SHELXL,” *Acta Crystallographica Section C: Structural Chemistry* **71**, (2015): 3–8.

[S5] Bruker, xprep Version 2014/2.

[S6] P. Pengo, G. D. Pantos, S. Otto, J. K. M. Sanders, “Efficient and Mild Microwave-Assisted Stepwise Functionalization of Naphthalenediimide with r-Amino Acids,” *The Journal of Organic Chemistry* **71**, (2006): 7063.

[S7] Y. Vonhausen, F. Würthner, “Concentration-, Temperature- and Solvent-Dependent Self-Assembly: Merocyanine Dimerization as a Showcase Example for Obtaining Reliable Thermodynamic Data,” *Chemistry–A European Journal* **29**, (2023): e202300359.

[S8] B. Teichmann, A. Krause, M.-J. Lin, F. Würthner, “Enantioselective Recognition of Helicenes by a Tailored Chiral Benzo[ghi]perylene Trisimide π-Scaffold,” *Angew. Chem. Int. Ed.* **61**, (2022): e202117625.
